# Indole-2-Carboxamide as an Effective Scaffold for the Design of New TRPV1 Agonists

**DOI:** 10.3390/molecules30030721

**Published:** 2025-02-05

**Authors:** Samuele Maramai, Claudia Mugnaini, Marco Paolino, Aniello Schiano Moriello, Luciano De Petrocellis, Federico Corelli, Francesca Aiello, Antonella Brizzi

**Affiliations:** 1 Department of Biotechnology, Chemistry and Pharmacy, University of Siena, Via Aldo Moro 2, 53100 Siena, Italy; claudia.mugnaini@unisi.it (C.M.); paolino3@unisi.it (M.P.); federico.corelli@unisi.it (F.C.); 2 Endocannabinoid Research Group, Istituto di Chimica Biomolecolare, Consiglio Nazionale delle Ricerche, Via Campi Flegrei 34, 80078 Pozzuoli, Italy; aniello.schianomoriello@icb.cnr.it (A.S.M.); luciano.depetrocellis@icb.cnr.it (L.D.P.); 3 Epitech Group SpA, 35030 Saccolongo, Italy; 4 Department of Pharmacy, Health and Nutritional Sciences, University of Calabria, 87036 Arcavacata di Rende, Italy; francesca.aiello@unical.it

**Keywords:** TRPV1 agonist, indole-2-carboxamides, pain, capsaicin

## Abstract

Due to its central role in pain, inflammation, and related disorders, the Transient Receptor Potential (TPR) Vanilloid Type-1 (TRPV1) ion channel represents an attractive target for the development of novel antinociceptive and anti-inflammatory agents. Capsaicin, the natural component of chili peppers, is one of the most investigated agonists of this receptor. Several modifications of its structure have been attempted, aiming at finding TRPV1 agonists with improved characteristics, but, to date, no capsaicin-derived agents have reached the market. Based on our previous knowledge of the design and synthesis of TRPV1 agonists, in this paper we propose two small series of indole-2-carboxamides as novel and selective agonists for this ion channel. The newly developed compounds have been structurally characterized and tested in vitro for their ability to modulate TRPV1, in terms of efficacy, potency (EC_50_), and desensitization (IC_50_) properties. For the most promising derivatives, selectivity over the TRP ankyrin-1 (TRPA1) channel has been reported. From our study, compound **6g** arose as a promising candidate for further evaluation, also in correlation with its in silico-predicted drug-like properties.

## 1. Introduction

**Figure 1:**
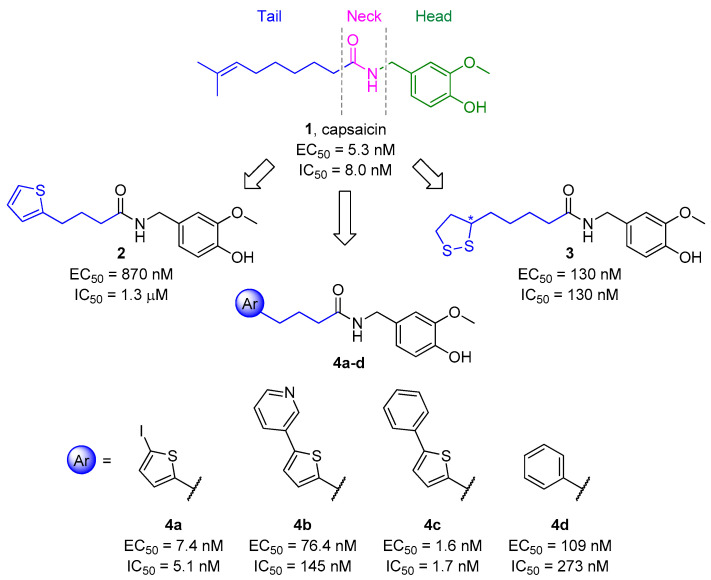
Structure of capsaicin (1) and previously reported TRPV1 agonists exemplified by lead compounds **2**, **3**, and **4a**–**d**. EC_50_/IC_50_ values refer to the potency and desensitization of the compounds on TRPV1, respectively.

The Transient Receptor Potential (TPR) Vanilloid Type-1 (TRPV1) ion channel is a polymodal nocitransducer member of the “thermo” TRP receptor family, densely expressed by spinal and cranial sensory neurons [1]. It has also been identified in neuron cell bodies and dendrites of higher brain areas [2] as well as in non-neuronal cells, such as astrocytes, immune and muscle cells, and adipocytes [3]. Structurally, TRPV1 is a homo- or hetero-tetramer, with each of the four subunits possessing 6-trans-membrane helices (S1–S6) and forming a cation-permeable pore between helices 5 and 6 [4]. Upon the activation of the receptor, the pore allows the influx of Ca^2+^ and, to a limited extent, of Na^+^, thus inducing cell membrane depolarization and Ca^2+^ release from the endoplasmic reticulum [5]. These Ca^2+^-dependent events finally lead to the desensitization of the receptor and the entire nerve, producing a paradoxical analgesic effect. TRPV1 reacts to several stimuli, including endogenous and exogenous agonists, toxins, and naturally occurring compounds, along with noxious heat or pH variations, therefore acting as a principal transducer of thermal, chemical, and mechanical stimuli [6,7,8]. The importance of TRPV1 in different physiological and pathological processes is demonstrated by its wide expression in various tissues and body compartments (e.g., gastrointestinal tract, skin, airways, etc.). Most importantly, TRPV1 is also upregulated in chronic pain conditions [9]. Therefore, this receptor represents a highly validated target [10] when it comes to developing novel therapeutic agents for pain [11] and inflammation [12]. Additionally, the modulation of TRPV1 activity has also been documented to have a central role in neuroprotection [13]. One of the most studied TRPV1 agonists is capsaicin [13,14] (**1**, Figure 1), the main pungent component of hot green and red peppers belonging to the genus Capsicum. Capsaicin, as well as other agonists like resiniferatoxin [15], activates TRPV1 and, following a prolonged application, induces the desensitization of the receptor. This results in pain relief due to a decrease in the TRPV1-mediated release of inflammatory molecules in response to noxious stimuli. Accordingly, the capsaicin-mediated activation of TRPV1 displayed strong anti-inflammatory and anti-cancer activities [16,17], directly influencing cellular crosstalk and fate [18]. To date, no drugs other than capsaicin have reached the market, but vivid interest is still paid to capsaicin-inspired analogs in the search for novel anti-inflammatory and antinociceptive agents. In fact, TRPV1 modulators have regained attention in line with the increased availability of detailed structural information [4] that guided the synthesis and investigation of a larger panel of molecules acting either as agonists or antagonists of the receptor [19]. Even if both these classes of agents are characterized by analgesic and anti-inflammatory actions, they have different mechanisms of action and are therefore not interchangeable in the clinic, where they tend to represent complementary pharmacological approaches [20]. 

**Figure 2:**
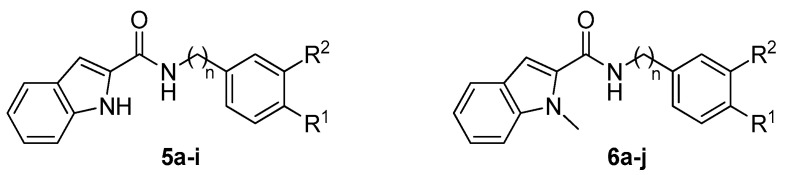
General structures of the title compounds **5a**–**i** and **6a**–**j**.

Our interest in TRPV1 prompted us to develop novel and selective agonists aiming at targeting this receptor over other isoforms or families (e.g., TRP-Ankyrin 1, TRPA1) and overcoming the pungency and systemic side effects of classical agonists. To this aim, rational modifications of the “tail” and the “head” portions of capsaicin have been proposed. We initially investigated the effect of replacing the aliphatic nonenyl chain of capsaicin (tail side) with the lipophilic 4-(thiophen-2-yl)butanoic acid, then changing the substituents on the vanillyl amine (head side) or the length of the carbon tether to build up different amides [21]. Several compounds behaved as activators of TRPV1, and the most promising analog of the series, bearing the vanillyl amine moiety (**2**, Figure 1), was initially evaluated for its biological properties, showing a higher neuroprotective effect than capsaicin in vitro and a slight but significant antinociceptive effect in vivo. With a similar perspective, we also explored the possibility of synthesizing different amides using α-lipoic acid, an organosulfur nutrient endowed with pleiotropic actions and a safe biological profile, as the tail portion [22]. Although this new series of compounds resulted in TRPV1 agonists that were slightly less potent than capsaicin, compound **3** (Figure 1), the lipoic analog of capsaicin, and a few other analogs were endowed with interesting radical-scavenger activity and protective properties against oxidative stress. More recently, in silico studies guided the rational design of novel TRPV1 modulators based on the structure of reference compound **2**. We investigated the possibility of adding an extra lipophilic moiety at position 5 of the thiophene ring, including an iodine atom and bulkier groups, such as phenyl and pyridine rings (**4a**–**c**, Figure 1). The replacement of the thiophene with a phenyl ring was also taken into consideration (**4d**, Figure 1). Following our novel approach, each of the new classes of compounds was expanded by synthesizing different amides with variously substituted benzyl- and phenethyl-amines and anilines [23]. Worth of note, compounds **4a** and **4c** displayed activation (EC_50_) and desensitization (IC_50_) potencies in the low nanomolar range, while the pyridine analog **4b** retained a nice profile with roughly an order of magnitude decrease in activity. Compound **4d** showed a significant loss of activity, in the sub-micromolar range, and was also characterized by a lower, although significant, activation of TRPA1. Accordingly, compound **4c** also suffered from a partial lack of selectivity, thus interacting with TRPA1 in the micromolar range. Therefore, **4a** and **4b** were selected for further evaluation, highlighting their protective properties against oxidative stress-induced ROS formation, with compound **4b** also exhibiting a potent antinociceptive effect in vivo. In the attempt to find additional scaffolds for the identification of novel TRPV1 agonists, in this paper, we report two novel series of compounds based on the indole and *N*-methylindole heterocycles (**5a**–**i** and **6a**–**j**, Figure 2). For this purpose, 1*H*-indole-2-carboxylic acid was selected because it provides derivatives with a less flexible structure, having the carboxylic group directly linked to the heteroaromatic nucleus, and a broad π-π system. Indoles are an important class of compounds frequently occurring in bioactive natural products [24] and in several approved drugs, making these heterocycles privileged scaffolds for the identification of novel biologically active substances [25]. Among the natural ligands of TRPV1, evodiamine, the major active component of the *Evodiae fructus*, is an indole alkaloid endowed with analgesic activity thanks to its ability to activate and desensitize TRPV1 channels in sensory neurons [26,27]. However, the literature has reported few examples of TRPV1 modulators based on the indole nucleus [28,29]. Therefore, we decided to embark with the synthesis and preliminary in vitro characterization of indole-2-carboxamides, taking inspiration from the “head” portion of capsaicin and using other substitution patterns as already reported in our previous works [21,22,23].

## 2. Results

### 2.1. Chemistry

**Scheme 1:**
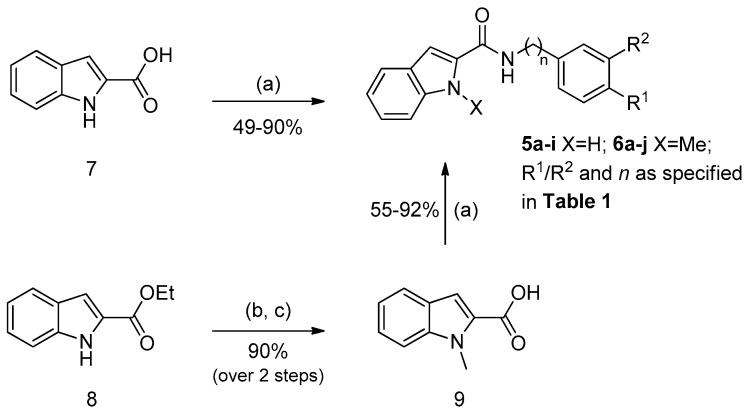
The synthetic procedure leading to the title compounds **5a**–**i** and **6a**–**j**. Reagents and conditions: (**a**) Method A: suitably substituted amine or aniline, EDCI, HOBt, dry DCM (**6b**–**f**, **6h**–**i**) or HPLC-grade ACN (**5a**–**e**, **5g**–**h**, **6a**), rt, 12 h; Method B: suitable amine hydrochloride, HBTU, HOBt, DIPEA, dry DMF, rt, 12 h (**5f**, **5i**, **6g**, **6j**); (**b**) DMC, K_2_CO_3_, dry DMF, 150 °C, 6 h; (**c**) EtOH/aq. NaOH, reflux, 4 h.

The synthesis of compounds **5a**–**i** and **6a**–**j** was relatively straightforward and followed the procedure reported in Scheme 1. The 1*H*-indole-2-carboxylic acid (**7**) was commercially available and was directly used in the coupling reaction with the different amines using two different condensing systems, a mixture of EDCI/HOBt, in dry dichloromethane (DCM) or acetonitrile (ACN), method A, or HBTU/HOBt and DIPEA as the base in dry *N,N*-dimethylformamide (DMF), method B. To obtain *N*-methylindole carboxamides, the appropriate carboxylic acid was synthesized starting from ethyl 1*H*-indole-2-carboxylate (**8**), which was first converted to the corresponding *N*-methyl derivative, using dimethyl carbonate (DMC) as the methylating agent in dry DMF [30], and then hydrolyzed to the free acid (**9**) by means of a refluxing ethanolic solution of NaOH. The latter was then coupled with the suitable amines following the above-mentioned procedures. The title compounds **5a**–**i** and **6a**–**j** were obtained with acceptable to good yields as light-colored or white solids after chromatographic purification.

### 2.2. In Vitro Characterization

Compounds **5a**–**i** and **6a**–**j** were progressed into TRPV1 functional evaluation, using human recombinant TRPV1 receptors stably overexpressed in human embryonic kidney (HEK-293) cells. The same in vitro model was used with rat recombinant TRPA1 receptors, this latter representing the other principal transducer of painful stimuli, to assess the compounds’ selectivity for TRPV1 [31]. In the functional assay, the effect of the tested compounds on the intracellular Ca^2+^ concentration was measured and correlated to their agonism (EC_50_) or antagonism/desensitization (IC_50_, determined against the effect of 0.1 µM capsaicin for TRPV1 or against the effect of 100 µM allyl isothiocyanate (AITC) for TRPA1) on the two ion channels. For the efficacy at TRPV1 and TRPA1, % values were normalized to the maximum Ca^2+^ influx effect on intracellular Ca^2+^ concentration observed with the application of 4 μM ionomycin or 100 μM AITC, respectively. The results are summarized in Table 1. Interestingly, most of the final amides, with the exception of a few derivatives, were found to be able to interact with at least one of the two TRP channels behaving as agonists with varying efficacy and potency.

## 3. Discussion

Two small series of indole-based amides were synthesized and tested in vitro for their effect on TRPV1 in the search for novel agonists that are potentially useful in pain and inflammatory disorders. At first glance, it is clear that the *N*-methylated analogs performed definitely better than the -NH counterparts. This result is not entirely unexpected, since the lipophilicity of the tail portion is reported to be essential to cross the cell membrane and, therefore, to reach the channel binding site [32]. Indeed, in the -NH series of amides, only compound **5f**, characterized by the vanillyl moiety in the amide head as in the natural agonist, showed a slight although significant agonist activity on TRPV1 (efficacy 66.8%, EC_50_ = 0.56 μM; IC_50_ = 1.03 μM). Derivatives **5a** and **5i**, both with the hydroxyl substituent in the *para* position of the aromatic head and an *n* value equal to 0 and 2, respectively, also behaved as TRPV1 ligands. However, while **5a** maintained moderate efficacy (54.0%) and acceptable potency (EC_50_ = 2.3 µM), **5i** turned out to be a very weak agonist. Therefore, we shifted our attention to the series of compounds **6a**–**j**. Amides **6a**, **6g,** and **6j** are the most interesting compounds, showing efficacy values higher than 60% and good potency, with their EC_50_ values ranging from high (0.34 µM and 0.18 µM for **6a** and **6j**, respectively) to low nanomolar (0.0365 µM for **6g**). In particular, compound **6g**, bearing once again the vanillic residue, was the best-performing analog of the series, displaying EC_50_ and IC_50_ values against TRPV1 in the nanomolar range (EC_50_ = 36.5 nM; IC_50_ = 53.5 nM) and efficacy similar to that of capsaicin (73.2% and 78.6%, respectively). Moreover, this compound failed in activating the TRPA1 receptor, therefore showing a nice and selective profile. Amides **6b** and **6d** still showed acceptable potency (EC_50_ = 3.5 and 1.1 µM, respectively) despite having poor efficacy (34.4% and 27.6%, respectively). Interestingly, compound **6b** behaved as a very good TRPA1 agonist, endowed with potency similar to that of AITC (EC_50_ = 2.0 and 1.41 µM, respectively) and even higher efficacy (78.6% and 65.9%, respectively). Chemically, the head portion of **6b** is characterized by a fluorine atom replacing the hydroxyl group. This substitution pattern seemed to shift the interaction from the TRPV1 to TRPA1 channel, since virtually all the other fluorinated amides, i.e., **5h**, **6f,** and **6i**, turned out to be effective TRPA1 ligands (efficacy values ranging from 68.4% to 100.6%), albeit less potent (EC_50_ = 19.1, 20.4 and 10.9 µM, respectively).

**Figure 3:**
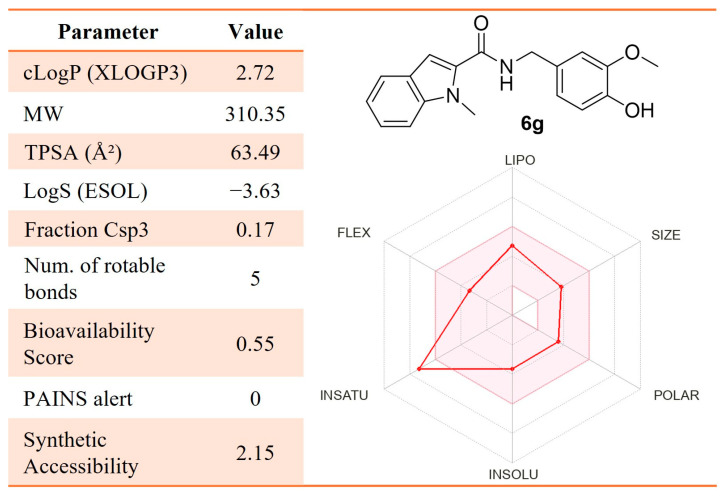
Structure and SwissADME property prediction [33] for compound **6g**. The red-colored zone in the spider web diagram is the suitable space for oral bioavailability. LIPO (lipophilicity): −0.7 < XLOGP3 < +5.0; SIZE: 150 g/mol < MW < 500 g/mol; POLAR (polarity): 20 Å^2^ < TPSA < 130 Å^2^; INSOLU (insolubility): −6 < Log S (ESOL) < 0; INSATU (insaturation): 0.25 < Fraction Csp3 < 1; FLEX (flexibility): 0 < num. Rotable bonds < 9.

Being the most interesting analog of the series, we decided to further evaluate in silico drug-like properties and brain permeability for compound **6g**. The calculations performed using SwissADME online tools revealed the potential for **6g** to be a brain-penetrant agent, although retaining a balanced water solubility, with a calculated LogP value of 2.93 (Figure 3). Additionally, it showed promising drug-like properties, a bioavailability score of 0.55, no PAINS alerts, and the synthetic accessibility score was 2.15 (on a scale from 1 to 10, where 10 is the least accessible value).

## 4. Experimental Section

### 4.1. Materials and Methods

All starting materials, reagents, and solvents were purchased from common commercial suppliers and were used as received without further purification. Anhydrous reactions were performed under a positive pressure flow of dry and inert gas (nitrogen or argon), and dried solvents were freshly prepared using the proper drying agents. Organic solutions were dried over anhydrous sodium sulfate and concentrated with a Bűchi rotary evaporator R-110 (R-110, Milan, Italy) equipped with a KNF N 820 FT 18 vacuum pump. Melting points were determined on a Kofler hot stage apparatus (K) or using a Gallenkamp melting point apparatus (G) and are uncorrected. Merck 60 silica gel, 230–400 mesh, was used for flash column chromatography (Milan, Italy). The purity of all compounds was checked by TLC on Merck 60 F254 silica gel on aluminum foils (Milan, Italy). The identity of the final compounds was unambiguously assessed by nuclear magnetic resonance, mass spectroscopy, and elemental analysis. ^1^H NMR and ^13^C NMR spectra were recorded on a Bruker Advance DPX400 or DPX600 MHz, in the indicated solvent at 25 °C, and chemical shifts were expressed as δ (ppm) relative to the residual solvent peak (Milan, Italy). The LC-MS analyses were performed on an Agilent 1100 series liquid chromatograph system, including a 1100 MSD model VL benchtop mass spectrometer with ESI source, a binary high-pressure gradient pump (0.4 mL/min low flow rate, employing a binary solvent system of 95/5 methanol/water), and a solvent degassing unit. Nitrogen (purity 99.995%) was used as nebulizer and drying gas. UV detection was monitored at 254 nm (Milan, Italy). Mass spectra were acquired in the positive or negative mode scanning over the mass range *m*/*z* of 150–1500. Elemental analyses were performed on a PerkinElmer elemental apparatus model 240 for C, H, N, and the data are within ±0.4% of the theoretical values. The chemical purity of most of the tested compounds was determined using an Acquity Waters UPLC-MS system equipped with Waters BEH C18 (2.1 mm × 50 mm, 1.7 µm) reversed-phase column and UV detector (254 nm). Analyses were carried out with a gradient elution, solvent A (0.1% formic acid in water), solvent B (0.1% formic acid in ACN) 90:10 to 0:100 over 2.9 min, at the flow rate of 0.5 mL/min, and UV detector at 254 nm (Milan, Italy). The chemical purities of compounds **5e**, **5g**–**i**, **6b**, **6f**, and **6i**–**j** were determined using a Bruker Elute HPLC with autosampler and UV-DAD, coupled with a Bruker TimsTOF spectrometer and API source. The chromatographic separation was performed with Phenomenex Kinetex EVO column (C18, 2.6 µm, 100 × 4.6 mm), injecting 5 μL of the sample. Analyses were carried out with a gradient elution, solvent A (water) and solvent B (ACN), 95:5 to 5:95 over 6 min followed by 4 min plateau at 5:95, at the flow rate of 2 mL/min and UV detector at 254 nm (Milan, Italy). All the tested compounds possessed a purity ≥ 95.0%.

Synthesis of 1-methyl-1*H*-indole-2-carboxylic acid (**9**).

To a stirred solution of ethyl 1*H*-indole-2-carboxylate **8** (1.0 eq.) in dry DMF (10 mL), kept under a positive pressure of dry nitrogen, were added, in the following order, K_2_CO_3_ (1.6 mol) and DMC (2.9 eq.). The reaction mixture was heated at 150 °C and maintained at this temperature for 6 h, changing its color from light yellow to dark green. After cooling, the reaction mixture was diluted with water forming a milky white solution, which was extracted several times with chloroform (×4). The collected organic layer was finally washed with a saturated NH_4_Cl solution (×2) and brine and dried over anhydrous Na_2_SO_4_. The filtration and evaporation of the solvent provided a crude material from which, after chromatographic purification, the desired pure *N*-methyl ester was obtained as a white solid (yield 85.0%, mp 39–42 °C (K)). The compound was then solubilized in ethanol and added with a measured excess of aqueous NaOH (3.0 eq., EtOH/H_2_O 3/1), refluxing the reaction mixture for 3–4 h and monitoring the disappearance of the ester. After cooling, the reaction mixture was acidified with diluted HCl to pH 2-3 and extracted with ethyl acetate (×4). After drying and the filtration of the organic phase, the solvent was concentrated to yield the pure acid **9** as a white solid (yield 95.0%, mp 196–198 °C (K). ^1^H NMR (400 MHz, Methanol-*d_4_*) δ 7.60 (d, 1H, *J* = 8.3 Hz), 7.41 (d, 1H, *J* = 8.3 Hz), 7.29 (t, 1H, *J* = 7.6 Hz), 7.23 (s, 1H), 7.07 (t, 1H, *J* = 7.6 Hz), 4.02 (s, 3H). MS (ESI) *m*/*z*: 174 [M-H]^−^ (100).

General procedure for the synthesis of indole-2-carboxamides **5a**–**i** and **6a**–**j**.

Method A. To a solution of the acid (1.0 eq., **7** or **9**) in dry DCM or HPLC-grade ACN (40 mL) was added the appropriate free amine (1.5 eq., 4-aminophenol, 4-fluoroaniline, benzylamine, 4-hydroxybenzylamine, 4-methoxybenzylamine, 4-fluorobenzylamine, 3,4-dichlorobenzylamine, and 3,4-difluorobenzylamine), keeping the reaction under a positive dry nitrogen atmosphere and at room temperature (rt). Afterwards, HOBt (1.2 eq.) and EDCI (1.5 eq.) were added in that order, leaving the reaction under stirring overnight. The next day, the reaction mixture was diluted with DCM, or the solvent (ACN) was removed under reduced pressure and the residue taken up with EtOAc. The organic phase was washed twice, first with a saturated NH_4_Cl solution and then with brine, and finally dried over anhydrous Na_2_SO_4_. Filtration and the evaporation of the solvent provided a crude product that was purified by flash chromatography on silica gel with the appropriate eluent.

Method B. To a solution of the acid (1.0 eq., **7** or **9**) in dry DMF (10 mL), kept under a positive pressure of dry nitrogen and at rt, were added in, the following order, HBTU (2.0 eq.), HOBt (1.0 eq.), DIPEA (1.5 eq.), and the amine hydrochloride (1.2 eq., 4-hydroxy-3-methoxybenzylamine hydrochloride or 3,4-dihydroxyphenethylamine hydrochloride), and the reaction was stirred for 40 min; then, DIPEA (1.5 eq.) was added again, leaving the reaction under stirring overnight. The day after, the reaction mixture was diluted with chloroform and the organic layer washed twice with a saturated NH_4_Cl solution, and finally once with brine. After drying over anhydrous Na_2_SO_4_, the solvent was filtered and then concentrated to obtain a crude product that was purified by flash chromatography on silica gel with the appropriate eluent.

*N*-(4-Hydroxyphenyl)-1*H*-indole-2-carboxamide (**5a**) [34]: prepared from 1*H*-indole-2-carboxylic acid **7** and 4-aminophenol, following method A (ACN as the solvent); eluent *n*-hexane/EtOAc 2/5; yield 85.0%; pale yellow crystals (from DCM); mp 141–144 °C (K). ^1^H NMR (400 MHz, Dimethyl sulfoxide-*d_6_*) δ 9.31 (s, 1H), 7.61 (d, *J* = 7.8 Hz, 1H), 7.51 (d, *J* = 8.8 Hz, 2H), 7.43 (d, *J* = 7.8 Hz, 1H), 7.30 (s, 1H), 7.17 (t, *J* = 7.8 Hz, 1H), 7.02 (t, *J* = 7.3 Hz, 1H), 6.73 (d, *J* = 8.8 Hz, 2H). ^13^C NMR (101 MHz, Dimethyl sulfoxide-*d_6_*) δ 159.8 (C=O), 154.1, 137.1, 132.2, 130.9, 127.6, 124.1, 122.6 (×2), 122.1, 120.4, 115.6 (×2), 112.8, 103.8. MS (ESI) *m*/*z*: 253 [M+H]^+^ (100). Elemental analysis for C_15_H_12_N_2_O_2_ found C, 71.29; H, 4.80; N, 11.13; calculated C, 71.42; H, 4.79; N, 11.10. Purity (UPLC-MS): 100.0%.

*N*-Benzyl-1*H*-indole-2-carboxamide (**5b**) [35]: prepared from 1*H*-indole-2-carboxylic acid **7** and benzylamine, following method A (ACN as the solvent); eluent CHCl_3_/MeOH 49/1; yield 90.0%; white solid; mp 96–98 °C (K). ^1^H NMR (400 MHz, Chloroform-*d*) δ 9.24 (br s, 1H), 7.62 (d, *J* = 7.8 Hz, 1H), 7.42 (d, *J* = 8.3 Hz, 1H), 7.36 (d, *J* = 4.4 Hz, 2H), 7.33–7.24 (m, 4H), 7.13 (t, *J* = 7.6 Hz, 1H), 6.82 (s, 1H), 6.41 (br s, 1H), 4.68 (d, *J* = 5.9 Hz, 2H). ^13^C NMR (101 MHz, Chloroform-*d*) δ 161.4 (C=O), 139.7, 136.9, 131.7, 128.3 (×2), 127.9, 127.5 (×2), 126.9, 123.6, 121.6, 119.8, 112.2, 102.0, 42.7. MS (ESI) *m*/*z*: 251 [M+H]^+^ (40), 273 [M+Na]^+^ (100). Elemental analysis for C_16_H_14_N_2_O found C, 76.65; H, 5.66; N, 11.16; calculated C, 76.78; H, 5.64; N, 11.19. Purity (UPLC-MS): 100.0%.

*N*-(4-Hydroxybenzyl)-1*H*-indole-2-carboxamide (**5c**) [36]: prepared from 1*H*-indole-2-carboxylic acid **7** and 4-hydroxybenzylamine, following method A (ACN as the solvent); eluent *n*-hexane/EtOAc 2/5; yield 88.0%; cream solid; mp 237–240 °C (K). ^1^H NMR (400 MHz, Acetone-*d_6_*) δ 8.19 (s, 1H), 8.11 (br s, 1H), 7.57 (d, 1H, *J* = 8.3 Hz), 7.51 (d, 1H, *J* = 8.3 Hz), 7.20–7.16 (m, 3H), 7.11 (s, 1H), 7.02 (t, 1H, *J* = 7.3 Hz), 6.76 (d, 2H, *J* = 8.8 Hz), 4.49 (d, 2H, *J* = 4.9 Hz). ^13^C NMR (101 MHz, Methanol-*d_4_*) δ 162.6 (C=O), 156.3, 136.9, 130.9, 129.6, 128.6 (×2), 127.6, 123.6, 121.3, 119.7, 114.9 (×2), 111.6, 103.1, 42.3. MS (ESI) *m*/*z*: 267 [M+H]^+^ (100). Elemental analysis for C_16_H_14_N_2_O_2_ found C, 72.34; H, 5.31; N, 10.50; calculated C, 72.17; H, 5.30; N, 10.52. Purity (UPLC-MS): 100.0%.

*N*-(4-Methoxybenzyl)-1*H*-indole-2-carboxamide (**5d**) [36]: prepared from 1*H*-indole-2-carboxylic acid **7** and 4-methoxybenzylamine, following method A (ACN as the solvent); eluent *n*-hexane/EtOAc 3/5; yield 70.0%; white needles (from DCM); mp 224–226 °C (K). ^1^H NMR (600 MHz, Acetone-*d_6_*) δ 7.47 (d, *J* = 8.0 Hz, 1H), 7.40 (d, *J* = 8.3 Hz, 1H), 7.18 (d, *J* = 8.6 Hz, 2H), 7.08 (t, *J* = 7.6 Hz, 1H), 7.01 (s, 1H), 6.92 (t, *J* = 7.5 Hz, 1H), 6.75 (d, *J* = 8.6 Hz, 2H), 4.41 (s, 2H), 3.63 (s, 3H). ^13^C NMR (151 MHz, Acetone-*d_6_*) δ 161.4 (C=O), 158.9, 136.8, 131.7, 131.5, 128.9 (×2), 127.8, 123.6, 121.6, 119.5, 113.1 (×2), 112.2, 102.3, 54.6, 42.1. MS (ESI) *m*/*z*: 281 [M+H]^+^ (100), 303 [M+Na]^+^ (45). Elemental analysis for C_17_H_16_N_2_O_2_ found C, 72.71; H, 5.74; N, 9.97; calculated C, 72.84; H, 5.75; N, 9.99. Purity (UPLC-MS): 100.0%.

*N*-(4-Fluorobenzyl)-1*H*-indole-2-carboxamide (**5e**) [37]: prepared from 1*H*-indole-2-carboxylic acid **7** and 4-fluorobenzylamine, following method A (ACN as the solvent); eluent CH_2_Cl_2_/MeOH 50/1; yield 68.0%; white solid; mp 196–197 °C (K). ^1^H NMR (600 MHz, Acetone-*d_6_*) δ 8.66 (s, 1H), 7.62 (d, *J* = 8.0 Hz, 1H), 7.53 (d, *J* = 8.3 Hz, 1H), 7.45 (dd, *J* = 7.8, 5.7 Hz, 2H), 7.25–7.20 (m, 2H), 7.11–7.05 (m, 3H), 4.65 (d, *J* = 6.0 Hz, 2H). ^13^C NMR (151 MHz, Acetone-*d_6_*) δ 162.0 (C=O), 161.9 (d, *J* = 242.8 Hz), 137.0, 135.7 (d, *J* = 3.1 Hz), 131.5, 129.5 (d, *J* = 8.1 Hz) (×2), 127.8, 123.8, 121.7, 120.1, 115.0 (d, *J* = 21.5 Hz) (×2), 112.3, 102.8, 42.1. MS (ESI) *m*/*z*: 269 [M+H]^+^ (100). Elemental analysis for C_16_H_13_FN_2_O found C, 71.76; H, 4.89; N, 10.41; calculated C, 71.63; H, 4.88; N, 10.44. Purity (HPLC-MS): 100.0%.

*N*-(4-Hydroxy-3-methoxybenzyl)-1*H*-indole-2-carboxamide (**5f**) [38]: prepared from 1*H*-indole-2-carboxylic acid **7** and 4-hydroxy-3-methoxybenzylamine hydrochloride, following method B; eluent *n*-hexane/EtOAc 2/5; yield 60.0%; pale pink solid; mp 178.8 °C (G). ^1^H NMR (600 MHz, Acetone-*d_6_*) δ 8.26 (br t, *J* = 5.0 Hz, 1H), 7.62 (d, *J* = 8.0 Hz, 1H), 7.56 (d, *J* = 8.3 Hz, 1H), 7.50 (br s, 1H), 7.23 (t, *J* = 8.2 Hz, 1H), 7.19 (d, *J* = 2.1 Hz, 1H), 7.07 (t, *J* = 7.5 Hz, 1H), 7.04 (d, *J* = 1.9 Hz, 1H), 6.87 (dd, *J* = 8.1, 1.9 Hz, 1H), 6.80 (d, *J* = 8.1 Hz, 1H), 4.58 (d, *J* = 6.0 Hz, 2H), 3.80 (s, 3H). ^13^C NMR (151 MHz, Acetone-*d_6_*) δ 161.4 (C=O), 147.4, 145.8, 136.9, 131.8, 130.8, 127.9, 123.6, 121.6, 120.4, 119.9, 114.8, 112.3, 111.5, 102.3, 55.4, 42.7. MS (ESI) *m*/*z*: 297 [M+H]^+^ (100). Elemental analysis for C_17_H_16_N_2_O_3_ found C, 68.67; H, 5.46; N, 9.48; calculated C, 68.91; H, 5.44; N, 9.45. Purity (UPLC-MS): 96.0%.

*N*-(3,4-Dichlorobenzyl)-1*H*-indole-2-carboxamide (**5g**) [38]: prepared from 1*H*-indole-2-carboxylic acid **7** and 3,4-dichlorobenzylamine, following method A (ACN as the solvent); eluent DCM/MeOH 49/1; yield 87.0%; white solid; mp 174.1 °C (G). ^1^H NMR (600 MHz, Acetone-*d_6_*) δ 8.50 (br s, 1H), 7.66–7.60 (m, 2H), 7.52 (d, *J* = 8.3 Hz, 2H), 7.39 (dd, *J* = 8.3, 1.6 Hz, 1H), 7.24 (t, *J* = 7.6 Hz, 1H), 7.20 (d, *J* = 1.9 Hz, 1H), 7.09 (t, *J* = 7.5 Hz, 1H), 4.68 (d, *J* = 6.1 Hz, 2H). ^13^C NMR (151 MHz, Acetone-*d_6_*) δ 161.8 (C=O), 140.8, 137.0, 131.7, 131.3, 130.5, 130.2, 129.5, 127.8, 127.6, 123.9, 121.7, 120.1, 112.3, 102.7, 41.8. MS (ESI) *m*/*z*: 320 [M+H]^+^ (100). Elemental analysis for C_16_H_12_Cl_2_N_2_O found C, 60.14; H, 3.80; N, 8.76; calculated C, 60.21; H, 3.79; N, 8.78. Purity (HPLC-MS): 100.0%.

*N*-(3,4-Difluorobenzyl)-1*H*-indole-2-carboxamide (**5h**): prepared from 1*H*-indole-2-carboxylic acid **7** and 3,4-difluorobenzylamine, following method A (ACN as the solvent); eluent DCM/MeOH 49/1; yield 78.0%; pale yellow solid; mp 212.8 °C (G). ^1^H NMR (600 MHz, Acetone-*d_6_*) δ 10.94 (s, 1H), 8.54 (s, 1H), 7.46 (d, *J* = 8.0 Hz, 1H), 7.36 (dd, *J* = 8.3, 0.7 Hz, 1H), 7.24–7.19 (m, 1H), 7.14–7.04 (m, 4H), 6.91 (t, *J* = 7.1 Hz, 1H), 4.48 (d, *J* = 6.2 Hz, 2H). ^13^C NMR (151 MHz, Acetone-*d_6_*) δ 161.9 (C=O), 149.9 (dd, *J* = 245.8, 12.8 Hz), 149.1 (dd, *J* = 244.7, 12.7 Hz), 137.4 (dd, *J* = 5.3, 3.8 Hz), 137.0, 131.3, 127.8, 124.0 (dd, *J* = 6.4, 3.5 Hz), 123.8, 121.7, 120.1, 117.1 (d, *J* = 17.2 Hz), 116.4 (d, *J* = 17.5 Hz), 112.3, 102.9, 41.8. MS (ESI) *m*/*z*: 287 [M+H]^+^ (100). Elemental analysis for C_16_H_12_F_2_N_2_O found C, 67.30; H, 4.22; N, 9.82; calculated C, 67.13; H, 4.23; N, 9.79. Purity (HPLC-MS): 98.2%.

*N*-(3,4-Dihydroxyphenethyl)-1*H*-indole-2-carboxamide (**5i**) [38]: prepared from 1*H*-indole-2-carboxylic acid **7** and 3,4-dihydroxyphenethylamine hydrochloride, following method B; eluent *n*-hexane/EtOAc 2/5; yield 49.0%; white solid; mp 221.5 °C (G). ^1^H NMR (600 MHz, Acetone-*d_6_*) δ 10.89 (s, 1H), 7.85 (s, 1H), 7.76 (br s, 1H), 7.45 (d, *J* = 8.0 Hz, 1H), 7.42 (d, *J* = 8.3 Hz, 1H), 7.07 (t, *J* = 7.6 Hz, 1H), 6.96 (s, 1H), 6.91 (t, *J* = 7.5 Hz, 1H), 6.65 (d, *J* = 1.8 Hz, 1H), 6.61 (d, *J* = 8.0 Hz, 1H), 6.45 (dd, *J* = 8.0, 1.8 Hz, 1H), 3.48 (dd, *J* = 13.9, 6.7 Hz, 2H), 2.66 (t, *J* = 7.5 Hz, 2H). ^13^C NMR (151 MHz, Acetone-*d_6_*) δ 161.7 (C=O), 145.0, 143.5, 136.9, 131.8, 131.1, 127.9, 123.6, 121.6, 120.0, 119.9, 115.8, 115.3, 112.3, 102.3, 41.3, 35.1. MS (ESI) *m*/*z*: 297 [M+H]^+^ (100). Elemental analysis for C_17_H_16_N_2_O_3_ found C, 68.74; H, 5.45; N, 9.47; calculated C, 68.91; H, 5.44; N, 9.45. Purity (HPLC-MS): 100.0%.

*N*-(4-Hydroxyphenyl)-1-methyl-1*H*-indole-2-carboxamide (**6a**): prepared from 1-methyl-1*H*-indole-2-carboxylic acid **9** and 4-aminophenol, following method A (ACN as the solvent); eluent CHCl_3_/MeOH 99/1; yield 55.0%; cream solid; mp 226–230 °C (K). ^1^H NMR (400 MHz, Methanol-*d_4_*) δ 7.60 (d, *J* = 8.3 Hz, 1H), 7.44–7.40 (m, 3H), 7.27 (t, *J* = 7.6 Hz, 1H), 7.11 (s, 1H), 7.08 (t, *J* = 7.6 Hz, 1H), 6.67 (d, *J* = 8.8 Hz, 2H), 3.98 (s, 3H). ^13^C NMR (101 MHz, Methanol-*d_4_*) δ 161.8 (C=O), 154.2, 139.3, 132.2, 130.0, 126.3, 123.8, 122.9 (×2), 121.5, 120.0, 114.9 (×2), 109.7, 105.1, 30.5. MS (ESI) *m*/*z*: 289 [M+Na]^+^ (100). Elemental analysis for C_16_H_14_N_2_O_2_ found C, 71.95; H, 5.32; N, 10.48; calculated C, 72.17; H, 5.30; N, 10.52. Purity (UPLC-MS): 96.0%.

*N*-(4-Fluorophenyl)-1-methyl-1*H*-indole-2-carboxamide (**6b**): prepared from 1-methyl-1*H*-indole-2-carboxylic acid **9** and 4-fluoroaniline, following method A (DCM as the solvent); eluent CHCl_3_/MeOH 99/1; yield 82.0%; pale yellow solid; mp 177–180 °C (K). ^1^H NMR (600 MHz, Acetone-*d_6_*) δ 9.81 (s, 1H), 7.91–7.84 (m, 2H), 7.65 (d, *J* = 8.0 Hz, 1H), 7.51 (d, *J* = 8.4 Hz, 1H), 7.33 (dt, *J* = 8.3, 1.1 Hz, 1H), 7.29 (s, 1H), 7.16–7.11 (m, 3H), 4.08 (s, 3H). ^13^C NMR (151 MHz, Acetone-*d_6_*) δ 160.8 (C=O), 159.0 (d, *J* = 240.8 Hz), 139.3, 135.50 (d, *J* = 2.7 Hz), 132.1, 126.2, 124.1, 122.03 (d, *J* = 7.8 Hz) (×2), 121.9, 120.3, 115.09 (d, *J* = 22.4 Hz) (×2), 110.3, 105.3, 31.0. MS (ESI) *m*/*z*: 269 [M+H]^+^ (100), 291 [M+Na]^+^ (20). Elemental analysis for C_16_H_13_FN_2_O found C, 71.72; H, 4.89; N, 10.42; calculated C, 71.63; H, 4.88; N, 10.44. Purity (HPLC-MS): 100.0%.

*N*-Benzyl-1-methyl-1*H*-indole-2-carboxamide (**6c**) [39]: prepared from 1-methyl-1*H*-indole-2-carboxylic acid **9** and benzylamine, following method A (DCM as the solvent); eluent CHCl_3_/MeOH 99/1; yield 72.0%; white solid; mp 157–159 °C (K). ^1^H NMR (400 MHz, Chloroform-*d*) δ 7.59 (d, *J* = 7.8 Hz, 1H), 7.38–7.30 (m, 7H), 7.14 (t, *J* = 7.3 Hz, 1H), 6.83 (s, 1H), 6.59 (br s, 1H), 4.62 (d, *J* = 5.9 Hz, 2H), 4.05 (s, 3H). ^13^C NMR (101 MHz, Chloroform-*d*) δ 162.6 (C=O), 139.1, 138.2, 131.9, 128.8 (×2), 127.8 (×2), 127.6, 126.1, 124.1, 121.8, 120.5, 110.2, 103.8, 43.6, 31.6. MS (ESI) *m*/*z*: 265 [M+H]^+^ (40), 287 [M+Na]^+^ (100). Elemental analysis for C_17_H_16_N_2_O found C, 77.35; H, 6.09; N, 10.62; calculated C, 77.25; H, 6.10; N, 10.60. Purity (UPLC-MS): 100.0%.

*N*-(4-hydroxybenzyl)-1-methyl-1*H*-indole-2-carboxamide (**6d**): prepared from 1-methyl-1*H*-indole-2-carboxylic acid **9** and 4-hydroxybenzylamine, following method A (DCM as the solvent); eluent *n*-hexane/EtOAc 2/3; yield 70.0%; pale pink solid; mp 164–168 °C (K). ^1^H NMR (600 MHz, Acetone-*d_6_*) δ 8.24 (s, 1H), 8.00 (br s, 1H), 7.42 (d, *J* = 8.0 Hz, 1H), 7.29 (d, *J* = 8.4 Hz, 1H), 7.11 (t, *J* = 7.7 Hz, 1H), 7.08 (d, *J* = 8.4 Hz, 2H), 6.95–6.90 (m, 2H), 6.65 (d, *J* = 8.5 Hz, 2H), 4.35 (br d, *J* = 5.6 Hz, 2H), 3.89 (s, 3H). ^13^C NMR (151 MHz, Acetone-*d_6_*) δ 162.2 (C=O), 156.6, 139.0, 132.5, 130.3, 129.0 (×2), 126.3, 123.6, 121.6, 120.1, 115.2 (×2), 110.2, 104.0, 42.3, 30.9. MS (ESI) *m*/*z*: 281 [M+H]^+^ (100). Elemental analysis for C_17_H_16_N_2_O_2_ found C, 72.75; H, 5.76; N, 10.00; calculated C, 72.84; H, 5.75; N, 9.99. Purity (UPLC-MS): 100.0%.

*N*-(4-methoxybenzyl)-1-methyl-1*H*-indole-2-carboxamide (**6e**): prepared from 1-methyl-1*H*-indole-2-carboxylic acid **9** and 4-methoxybenzylamine, following method A (DCM as the solvent); eluent CHCl_3_; yield 62.0%; white solid; mp 113–115 °C (K). ^1^H NMR (400 MHz, Chloroform-*d*) δ 7.57 (d, *J* = 7.8 Hz, 1H), 7.37–7.23 (m, 4H), 7.12 (t, *J* = 7.3 Hz, 1H), 6.87 (d, *J* = 7.8 Hz, 2H), 6.80 (s, 1H), 6.53 (br s, 1H), 4.54 (d, *J* = 5.3 Hz, 2H), 4.04 (s, 3H), 3.77 (s, 3H). ^13^C NMR (101 MHz, Chloroform-*d*) δ 162.5 (C=O), 159.2, 139.1, 132.0, 130.3, 129.2 (×2), 126.0, 124.1, 121.8, 120.7, 114.2 (×2), 110.2, 103.8, 55.3, 43.1, 31.6. MS (ESI) *m*/*z*: 295 [M+H]^+^ (10), 317 [M+Na]^+^ (100). Elemental analysis for C_18_H_18_N_2_O_2_ found C, 73.30; H, 6.17; N, 9.50; calculated C, 73.45; H, 6.16; N, 9.52. Purity (UPLC-MS): 100.0%.

*N*-(4-fluorobenzyl)-1-methyl-1*H-*indole-2-carboxamide (**6f**): prepared from 1-methyl-1*H*-indole-2-carboxylic acid **9** and 4-fluorobenzylamine, following method A (DCM as the solvent); eluent CHCl_3_/MeOH 49/1; yield 86.0%; white solid; mp 176.9 °C (G). ^1^H NMR (600 MHz, Acetone-*d_6_*) δ 8.27 (br s, 1H), 7.62 (d, *J* = 8.0 Hz, 1H), 7.50 (d, *J* = 8.4 Hz, 1H), 7.46 (dd, *J* = 8.8, 5.5 Hz, 2H), 7.30 (dd, *J* = 8.3, 1.1 Hz, 1H), 7.14–7.08 (m, 3H), 4.61 (d, *J* = 6.1 Hz, 2H), 4.09 (s, 3H). ^13^C NMR (151 MHz, Acetone-*d_6_*) δ 162.2 (C=O), 161.9 (d, *J* = 242.7 Hz), 139.0, 135.9 (d, *J* = 3.1 Hz), 132.2, 129.5 (d, *J* = 8.1 Hz) (×2), 126.3, 123.7, 121.6, 120.1, 114.9 (d, *J* = 21.5 Hz) (×2), 110.2, 104.0, 41.9, 30.9. MS (ESI) *m*/*z*: 283 [M+H]^+^ (35), 305 [M+Na]^+^ (100). Elemental analysis for C_17_H_15_FN_2_O found C, 72.41; H, 5.35; N, 9.90; calculated C, 72.33; H, 5.36; N, 9.92. Purity (HPLC-MS): 100.0%.

*N*-(4-hydroxy-3-methoxybenzyl)-1-methyl-1*H*-indole-2-carboxamide (**6g**): prepared from 1-methyl-1*H*-indole-2-carboxylic acid **9** and 4-hydroxy-3-methoxybenzylamine hydrochloride, following method B; eluent CHCl_3_/MeOH 47/3; yield 50.0%; pale pink solid; mp 135–138 °C (G). ^1^H NMR (400 MHz, Chloroform-*d*) δ 7.57 (d, *J* = 7.8 Hz, 1H), 7.36 (d, *J* = 8.3 Hz, 1H), 7.30 (t, *J* = 7.6 Hz, 1H), 7.12 (t, *J* = 7.6 Hz, 1H), 6.88–6.83 (m, 3H), 6.81 (s, 1H), 6.55 br (s, 1H), 4.52 (d, *J* = 5.4 Hz, 2H), 4.05 (s, 3H), 3.83 (s, 3H). ^13^C NMR (101 MHz, Chloroform-*d*) δ 162.6 (C=O), 146.9, 145.3, 139.1, 131.9, 130.1, 126.0, 124.1, 121.8, 120.8, 120.5, 114.6, 110.7, 110.2, 103.9, 56.0, 43.6, 31.6. MS (ESI) *m*/*z*: 311 [M+H]^+^ (10), 333 [M+Na]^+^ (100). Elemental analysis for C_18_H_18_N_2_O_3_ found C, 69.57; H, 5.84; N, 9.01; calculated C, 69.66; H, 5.85; N, 9.03. Purity (UPLC-MS): 100.0%.

*N*-(3,4-Dichlorobenzyl)-1-methyl-1*H*-indole-2-carboxamide (**6h**): Prepared from 1-methyl-1*H*-indole-2-carboxylic acid **9** and 3,4-dichlorobenzylamine, following method A (DCM as the solvent); eluent DCM; yield 82,0%; white solid; mp 147–150 °C (K). ^1^H NMR (600 MHz, Acetone-*d_6_*) δ 8.26 (br s, 1H), 7.49–7.44 (m, 2H), 7.37 (d, *J* = 8.3 Hz, 1H), 7.33 (d, *J* = 8.4 Hz, 1H), 7.24 (dd, *J* = 8.3, 1.8 Hz, 1H), 7.15 (t, *J* = 7.7 Hz, 1H), 6.99 (s, 1H), 6.95 (t, *J* = 7.5 Hz, 1H), 4.45 (d, *J* = 6.0 Hz, 2H), 3.92 (s, 3H). ^13^C NMR (151 MHz, Acetone-*d_6_*) δ 162.4 (C=O), 141.0, 139.1, 131.9, 131.6, 130.5, 130.12, 129.6, 127.6, 126.2, 123.8, 121.7, 120.2, 110.2, 104.3, 41.7, 30.9. MS (ESI) *m*/*z*: 334 [M+H]^+^ (15), 356 [M+Na]^+^ (100). Elemental analysis for C_17_H_14_Cl_2_N_2_O found C, 61.15; H, 4.25; N, 8.43; calculated C, 61.28; H, 4.24; N, 8.41. Purity (UPLC-MS): 100.0%.

*N*-(3,4-Difluorobenzyl)-1-methyl-1*H*-indole-2-carboxamide (**6i**): Prepared from 1-methyl-1*H*-indole-2-carboxylic acid **9** and 3,4-difluorobenzylamine, following method A (DCM as the solvent); eluent DCM; yield 90.0%; white solid; mp 113–115 °C (K). ^1^H NMR (600 MHz, Acetone-*d_6_*) δ 8.35 (br s, 1H), 7.62 (d, *J* = 8.0 Hz, 1H), 7.50 (d, *J* = 8.4 Hz, 1H), 7.42–7.36 (m, 1H), 7.31 (t, *J* = 8.2 Hz, 1H), 7.29–7.22 (m, 2H), 7.15 (s, 1H), 7.12 (t, *J* = 7.5 Hz, 1H), 4.61 (d, *J* = 6.2 Hz, 2H), 4.09 (s, 3H). ^13^C NMR (151 MHz, Acetone-*d_6_*) δ 162.3 (C=O), 150.0 (dd, *J* = 245.8, 12.8 Hz), 149.1 (dd, *J* = 244.7, 12.7 Hz), 139.1, 137.6 (dd, *J* = 5.4, 3.8 Hz), 132.0, 126.3, 124.0 (dd, *J* = 6.4, 3.5 Hz), 123.8, 121.6, 120.2, 117.1 (d, *J* = 17.3 Hz), 116.4 (d, *J* = 17.5 Hz), 110.2, 104.2, 41.7, 30.9. MS (ESI) *m*/*z*: 301 [M+H]^+^ (100). Elemental analysis for C_17_H_14_F_2_N_2_O found C, 68.19; H, 4.71; N, 9.31; calculated C, 67.99; H, 4.70; N, 9.33. Purity (HPLC-MS): 100.0%.

*N*-(3,4-Dihydroxyphenethyl)-1-methyl-1*H*-indole-2-carboxamide (**6j**): Prepared from 1-methyl-1*H*-indole-2-carboxylic acid **9** and 3,4-dihydroxyphenethylamine hydrochloride, following method B; eluent DCM; yield %; white solid; mp °C (G). ^1^H NMR (600 MHz, Acetone-*d_6_*) δ 7.74 (br s, 1H), 7.65 (br s, 1H), 7.44 (d, *J* = 7.9 Hz, 1H), 7.32 (d, *J* = 8.3 Hz, 1H), 7.13 (t, *J* = 7.6 Hz, 1H), 6.94 (t, *J* = 7.4 Hz, 1H), 6.85 (s, 1H), 6.64 (s, 1H), 6.61 (d, *J* = 7.9 Hz, 1H), 6.46 (d, *J* = 7.8 Hz, 1H), 3.90 (s, 3H), 3.42 (t, *J* = 6.8 Hz, 2H), 2.64 (t, *J* = 7.2 Hz, 2H). ^13^C NMR (151 MHz, Acetone-*d_6_*) δ 162.2 (C=O), 145.0, 143.5, 138.9, 132.7, 131.2, 126.3, 123.5, 121.5, 120.1, 120.0, 115.8, 115.2, 110.1, 103.7, 41.1, 35.1, 30.8. MS (ESI) *m*/*z*: 311 [M+H]^+^ (25), 333 [M+Na]^+^ (100). Elemental analysis for C_18_H_18_N_2_O_3_ found C, 69.72; H, 5.84; N, 9.04; calculated C, 69.66; H, 5.85; N, 9.03. Purity (HPLC-MS): 100.0%

### 4.2. TRPV1 and TRPA1 Channel Assay

The effect of tested compounds was determined by measuring the intracellular calcium ion concentration ([Ca^2+^]_i_) in human embryonic kidney (HEK) cells stably overexpressing recombinant human TRPV1 (*h*TRPV1) or rat TRPA1 (*r*TRPA1) loaded with the methyl ester of Fluo-4-AM (4 µM, Invitrogen), as previously described [40]. [Ca^2+^]_i_ was evaluated before and after the addition of various concentrations of the test compounds by measuring cell fluorescence at room temperature (*λ*_EX_ = 488 nm, *λ*_EM_ = 516 nm). The values of the effect on [Ca^2+^]_i_ in wild-type HEK293 cells were taken as baseline and subtracted from the values obtained from transfected cells. The efficacy of TRPV1/TRPA1 agonists was determined by normalizing their effect to the maximum Ca^2+^ influx effect on [Ca^2+^]_i_ observed with the application of 4 μM ionomycin, for TRPV1, or as a percentage of the effect obtained with 100 μM AITC, for TRPA1. Potency was expressed as the concentration of the test compounds exerting a half-maximal agonist effect (i.e., half-maximal increases [Ca^2+^]_i_) (EC_50_). The antagonist/desensitizing behavior was evaluated against capsaicin (0.1 μM) for TRPV1 and AITC (100 μM) for TRPA1, by adding each test compound in the quartz cuvette 5 min before the stimulation of the cells with agonists. The effect on [Ca^2+^]_i_ exerted by the agonist alone was taken as 100%. Data are expressed as the concentration exerting a half-maximal inhibition of agonist-induced [Ca^2+^]_i_ elevation (IC_50_). Curve fitting (sigmoidal dose−response variable slope) and parameter estimation were performed with GraphPad Prism (version 9.2.0, GraphPad Software Inc., San Diego, CA, USA). All determinations were performed at least in triplicate.

## 5. Conclusions

In conclusion, in this paper, we reported two small libraries of compounds based on the indole-2-carboxamide scaffold, possibly useful as TRPV1 modulators. These compounds were tested in vitro for their efficacy, potency, and activity on both TRPV1 and TRPA1 channels, highlighting their potential for building up potent and selective TRPV1 agonists. In fact, amongst all the synthesized analogs, compound **6g** displayed a nice efficacy profile, similar to that of the natural agonist capsaicin, accompanied by potency and desensitization values against TRPV1 in the nanomolar range. Additionally, **6g** showed high selectivity over the TRPA1 channel. Nevertheless, compound **6g** has not yet been investigated for its irritant properties and effects on body temperature, possibly impacting on its therapeutic value. However, considering the promising calculated drug-like properties and the high potential to be a brain penetrant agent, **6g** represents a valuable TRPV1 agonist deserving further in vitro and in vivo evaluation in the search for novel antinociceptive and anti-inflammatory agents.

## 6. Patents

Brizzi, A.; Aiello, F.; Corelli, F.; Ligandi TRPV1. 2014, N° 0001424275 (FI 2014 A000096).

## Figures and Tables

**Table 1:** Efficacy, potency (EC_50_), and antagonism/desensitization (IC_50_) values ^a^ of amides **5a**–**i** and **6a**–**j**, capsaicin, and AITC on TRPV1 and TRPA1 channels.

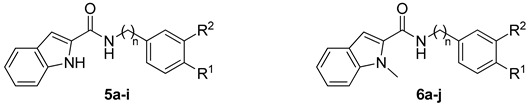									
Nr	n	R^1^	R^2^	TRPV1 Eff. ^b^ %	TRPV1 EC_50_ (μM)	TRPV1 IC_50_ (μM) ^c^	TRPA1 Eff. ^d^ %	TRPA1 EC_50_ (μM)	TRPA1 IC_50_ (μM) ^e^
**5a**	0	-OH	-H	54.1 ± 0.1	2.3 ± 0.1	10.0 ± 3.9	<10	n.a.	>100
**5b**	1	-H	-H	<10	n.a. ^f^	>100	<10	n.a.	>100
**5c**	1	-OH	-H	47.2 ± 0.3	>10	>50	<10	n.a.	>100
**5d**	1	-OCH_3_	-H	<10	n.a.	>50	39.1 ± 3.5	>50	>100
**5e**	1	-F	-H	<10	n.a.	>100	42.8 ± 1.8	23.7 ± 2.1	>50
**5f**	1	-OH	-OCH_3_	66.8 ± 1.3	0.56 ± 0.08	1.03 ± 0.09	n.t. ^g^	n.t.	n.t.
**5g**	1	-Cl	-Cl	<10	n.a.	>100	n.t.	n.t.	n.t.
**5h**	1	-F	-F	<10	n.a.	>100	68.4 ± 5.1	19.1 ± 3.1	>50
**5i**	2	-OH	-OH	37.3 ± 0.1	8.1 ± 0.10	2.1 ± 0.10	n.t	n.t	n.t
**6a**	0	-OH	-H	64.9 ± 0.6	0.34 ± 0.01	0.72 ± 0.08	42.9 ± 0.5	22.7 ± 1.7	>50
**6b**	0	-F	-H	34.4 ± 4.5	3.5 ± 2.1	6.9 ± 2.6	78.6 ± 9.7	2.0 ± 1.1	5.2 ± 0.4
**6c**	1	-H	-H	<10	n.a.	>50	<10	n.a.	>50
**6d**	1	-OH	-H	27.6 ± 0.3	1.1 ± 0.1	19.2 ± 6.7	<10	n.a.	>100
**6e**	1	-OCH_3_	-H	<10	n.a.	>10	81.3 ± 1.8	20.3 ± 1.1	>50
**6f**	1	-F	-H	<10	n.a.	>100	100.6 ± 11.1	20.4 ± 6.9	33.5 ± 3.2
**6g**	1	-OH	-OCH_3_	**73.2 ± 0.4**	**0.0365** **± 0.0011**	**0.0535** **± 0.048**	59.8 ± 1.6	>50	>100
**6h**	1	-Cl	-Cl	<10	n.a.	16.4 ± 0.5	n.t	n.t	n.t
**6i**	1	-F	-F	<10	n.a.	>100	76.1 ± 2.6	10.9 ± 0.9	32.2 ± 3.2
**6j**	2	-OH	-OH	70.8 ± 0.6	0.18 ± 0.05	0.26 ± 0.02	n.t	n.t	n.t
capsaicin				78.6 ± 0.6	0.0053 ± 0.0004	0.0080 ± 0.0003			
AITC							65.9 ± 0.5 ^b^	1.41 ± 0.04	1.71 ± 0.06

^a^ Data are means ± SEM of N = 3 determinations; ^b^ % determined as a percent of 4 μM ionomycin; ^c^ determined against the effect of capsaicin 0.1 μM; ^d^ % determined as a percent of 100 µM AITC; ^e^ determined against the effect of 100 µM AITC; ^f^ n.a. = not active. ^g^ n.t. = not tested.

## Data Availability

Data are contained within the article or Supplementary Materials.

## Supplementary Materials

The following supporting information can be downloaded at: https://www.mdpi.com/article/10.3390/molecules30030721/s1. ^1^H and ^13^C NMR analyses, and UPLC-MS and LC-MS traces.

## References

[1] Zhang M., Ma Y., Ye X., Zhang N., Pan L., Wang B. (2023). TRP (Transient Receptor Potential) Ion Channel Family: Structures, Biological Functions and Therapeutic Interventions for Diseases. Signal Transduct. Target. Ther..

[2] Menigoz A., Boudes M. (2011). The Expression Pattern of TRPV1 in Brain. J. Neurosci..

[3] Maximiano T.K.E., Carneiro J.A., Fattori V., Verri W.A. (2024). TRPV1: Receptor Structure, Activation, Modulation and Role in Neuro-Immune Interactions and Pain. Cell Calcium.

[4] Liao M., Cao E., Julius D., Cheng Y. (2013). Structure of the TRPV1 Ion Channel Determined by Electron Cryo-Microscopy. Nature.

[5] Yang F., Zheng J. (2017). Understand Spiciness: Mechanism of TRPV1 Channel Activation by Capsaicin. Protein Cell.

[6] Saito S., Tominaga M. (2017). Evolutionary Tuning of TRPA1 and TRPV1 Thermal and Chemical Sensitivity in Vertebrates. Temperature.

[7] Nozadze I., Tsiklauri N., Gurtskaia G., Tsagareli M.G. (2016). Role of Thermo TRPA1 and TRPV1 Channels in Heat, Cold, and Mechanical Nociception of Rats. Behav. Pharmacol..

[8] Willis W.D. (2009). The Role of TRPV1 Receptors in Pain Evoked by Noxious Thermal and Chemical Stimuli. Exp. Brain Res..

[9] González-Ramírez R., Chen Y., Liedtke W.B., Morales-Lázaro S.L. (2017). TRP Channels and Pain. Neurobiology of TRP Channels.

[10] Chahl L.A. (2024). TRPV1 Channels in the Central Nervous System as Drug Targets. Pharmaceuticals.

[11] Marrone M.C., Morabito A., Giustizieri M., Chiurchiù V., Leuti A., Mattioli M., Marinelli S., Riganti L., Lombardi M., Murana E. (2017). TRPV1 Channels Are Critical Brain Inflammation Detectors and Neuropathic Pain Biomarkers in Mice. Nat. Commun..

[12] Alawi K., Keeble J. (2010). The Paradoxical Role of the Transient Receptor Potential Vanilloid 1 Receptor in Inflammation. Pharmacol. Ther..

[13] Abdel-Salam O.M.E., Mózsik G. (2023). Capsaicin, The Vanilloid Receptor TRPV1 Agonist in Neuroprotection: Mechanisms Involved and Significance. Neurochem. Res..

[14] Maharjan A., Vasamsetti B.M.K., Park J.-H. (2024). A Comprehensive Review of Capsaicin: Biosynthesis, Industrial Productions, Processing to Applications, and Clinical Uses. Heliyon.

[15] Raisinghani M., Pabbidi R.M., Premkumar L.S. (2005). Activation of Transient Receptor Potential Vanilloid 1 (TRPV1) by Resiniferatoxin. J. Physiol..

[16] Adetunji T.L., Olawale F., Olisah C., Adetunji A.E., Aremu A.O. (2022). Capsaicin: A Two-Decade Systematic Review of Global Research Output and Recent Advances Against Human Cancer. Front. Oncol..

[17] Chapa-Oliver A., Mejía-Teniente L. (2016). Capsaicin: From Plants to a Cancer-Suppressing Agent. Molecules.

[18] Bujak J.K., Kosmala D., Szopa I.M., Majchrzak K., Bednarczyk P. (2019). Inflammation, Cancer and Immunity—Implication of TRPV1 Channel. Front. Oncol..

[19] Caballero J. (2022). A New Era for the Design of TRPV1 Antagonists and Agonists with the Use of Structural Information and Molecular Docking of Capsaicin-like Compounds. J. Enzyme Inhib. Med. Chem..

[20] Knotkova H., Pappagallo M., Szallasi A. (2008). Capsaicin (TRPV1 Agonist) Therapy for Pain Relief. Clin. J. Pain..

[21] Aiello F., Badolato M., Pessina F., Sticozzi C., Maestrini V., Aldinucci C., Luongo L., Guida F., Ligresti A., Artese A. (2016). Design and Synthesis of New Transient Receptor Potential Vanilloid Type-1 (TRPV1) Channel Modulators: Identification, Molecular Modeling Analysis, and Pharmacological Characterization of the N-(4-Hydroxy-3-Methoxybenzyl)-4-(Thiophen-2-yl)Butanamide, a Small Molecule Endowed with Agonist TRPV1 Activity and Protective Effects against Oxidative Stress. ACS Chem. Neurosci..

[22] Brizzi A., Maramai S., Aiello F., Baratto M.C., Corelli F., Mugnaini C., Paolino M., Scorzelli F., Aldinucci C., De Petrocellis L. (2022). Lipoic/Capsaicin-Related Amides: Synthesis and Biological Characterization of New TRPV1 Agonists Endowed with Protective Properties against Oxidative Stress. Int. J. Mol. Sci..

[23] Romeo I., Brizzi A., Pessina F., Ambrosio F.A., Aiello F., Belardo C., Carullo G., Costa G., De Petrocellis L., Frosini M. (2023). In Silico -Guided Rational Drug Design and Synthesis of Novel 4-(Thiophen-2-yl)Butanamides as Potent and Selective TRPV1 Agonists. J. Med. Chem..

[24] Kaushik N., Kaushik N., Attri P., Kumar N., Kim C., Verma A., Choi E. (2013). Biomedical Importance of Indoles. Molecules.

[25] Liu F., Su M. (2023). Indole and Indoline Scaffolds in Drug Discovery. Privileged Scaffolds in Drug Discovery.

[26] De Petrocellis L., Schiano Moriello A., Fontana G., Sacchetti A., Passarella D., Appendino G., Di Marzo V. (2014). Effect of chirality and lipophilicity in the functional activity of evodiamine and its analogues at TRPV1 channels. Br. J. Pharmacol..

[27] Iwaoka E., Wang S., Matsuyoshi N., Kogure Y., Aoki S., Yamamoto S., Noguchi K., Dai Y. (2016). Evodiamine suppresses capsaicin-induced thermal hyperalgesia through activation and subsequent desensitization of the transient receptor potential V1 channels. J. Nat. Med..

[28] Dewaker V., Sharma A.R., Debnath U., Park S.T., Kim H.S. (2023). Insights from molecular dynamics simulations of TRPV1 channel modulators in pain. Drug Discov. Today.

[29] Qiao Z., Liu S., Zhai W., Jiang L., Ma Y., Zhang Z., Wang B., Shao I., Qian H., Zhao F. (2024). Novel dual-target FAAH and TRPV1 ligands as potential pharmacotherapeutics for pain management. Eur. J. Med. Chem..

[30] Jiang X., Tiwari A., Thompson M., Chen Z., Cleary T.P., Lee T.B.K. (2001). A Practical Method for N-Methylation of Indoles Using Dimethyl Carbonate. Org. Process. Res. Dev..

[31] Brizzi A., Aiello F., Marini P., Cascio M.G., Corelli F., Brizzi V., De Petrocellis L., Ligresti A., Luongo L., Lamponi S. (2014). Structure–Affinity Relationships and Pharmacological Characterization of New Alkyl-Resorcinol Cannabinoid Receptor Ligands: Identification of a Dual Cannabinoid Receptor/TRPA1 Channel Agonist. Bioorg. Med. Chem..

[32] Ursu D., Knopp K., Beattie R.E., Liu B., Sher E. (2010). Pungency of TRPV1 agonists is directly correlated with kinetics of receptor activation and lipophilicity. Eur. J. Pharmacol..

[33] Daina A., Michielin O., Zoete V. (2017). SwissADME: A Free Web Tool to Evaluate Pharmacokinetics, Drug-Likeness and Medicinal Chemistry Friendliness of Small Molecules. Sci. Rep..

[34] Milkiewicz K.L., Marsilje T.H., Woodworth R.P., Bifulco N., Hangauer M.J., Hangauer D.G. (2000). The Design, Synthesis and Activity of Non-ATP Competitive Inhibitors of Pp60c-Src Tyrosine Kinase. Part 2: Hydroxyindole Derivatives. Bioorg. Med. Chem. Lett..

[35] La Regina G., Silvestri R., Gatti V., Lavecchia A., Novellino E., Befani O., Turini P., Agostinelli E. (2008). Synthesis, Structure–Activity Relationships and Molecular Modeling Studies of New Indole Inhibitors of Monoamine Oxidases A and B. Bioorg. Med. Chem..

[36] Hangauer D.G. (2008). Bicyclic Compositions and Methods for Modulating a Kinase Cascade.

[37] Ölgen S., Çoban T. (2002). Synthesis and Antioxidant Properties of Novel N-Substituted Indole-2-Carboxamide and Indole-3-Acetamide Derivatives. Arch. Pharm..

[38] Brizzi A., Aiello F., Corelli F. (2014). Ligandi TRPV1.

[39] Li W., Ma Y., Ju H., Huang W. (2016). Amine Compound Containing Aromatic Ring, and Preparation Method and Application Thereof.

[40] Schiano Moriello A., De Petrocellis L., Vitale R.M. (2023). Fluorescence-Based Assay for TRPV1 Channels. Methods Mol. Biol..

