# Supplementary material for: Indole-2-Carboxamide as an Effective Scaffold for the Design of New TRPV1 Agonists
Source: Molecules. 2025 Feb 5;30(3):721. doi: 10.3390/molecules30030721 (PMC11820618; doi:10.3390/molecules30030721)
Supplement: Supplementary file 1 [file molecules-30-00721-s001.zip › molecules-3379590-supplementary.pdf]

"Supplementary Materials"

Indole-2-carboxamide as an effective scaffold for the design of new TRPV1 agonists

Samuele Maramai [1,*], Claudia Mugnaini [1], Marco Paolino [1], Aniello Schiano Moriello [2], Luciano De Petrocellis [2], Federico Corelli [1], Francesca Aiello [3,†] and Antonella Brizzi [1,†,*]

Table of contents

**S2**	^{1}H-NMR of ethyl 1-methyl-1*H*-indole-2-carboxylate.
**S3**	^{1}H-NMR of 1-methyl-1*H*-indole-2-carboxylic acid **9**.
**S4-36**	^{1}H-NMR, ^{13}C-NMR and UPLC- or HPLC-MS of 1*H*-indole-2-carboxyamides **5a-i**.
**S37-73**	^{1}H-NMR, ^{13}C-NMR and UPLC- or HPLC-MS of 1*H*-indole-2-carboxyamides **6a-j**.

.

Ethyl 1-methyl-1*H*-indole-2-carboxylate

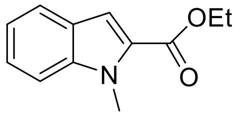

$C_{12}H_{13}NO_2$ **MW 203.24**

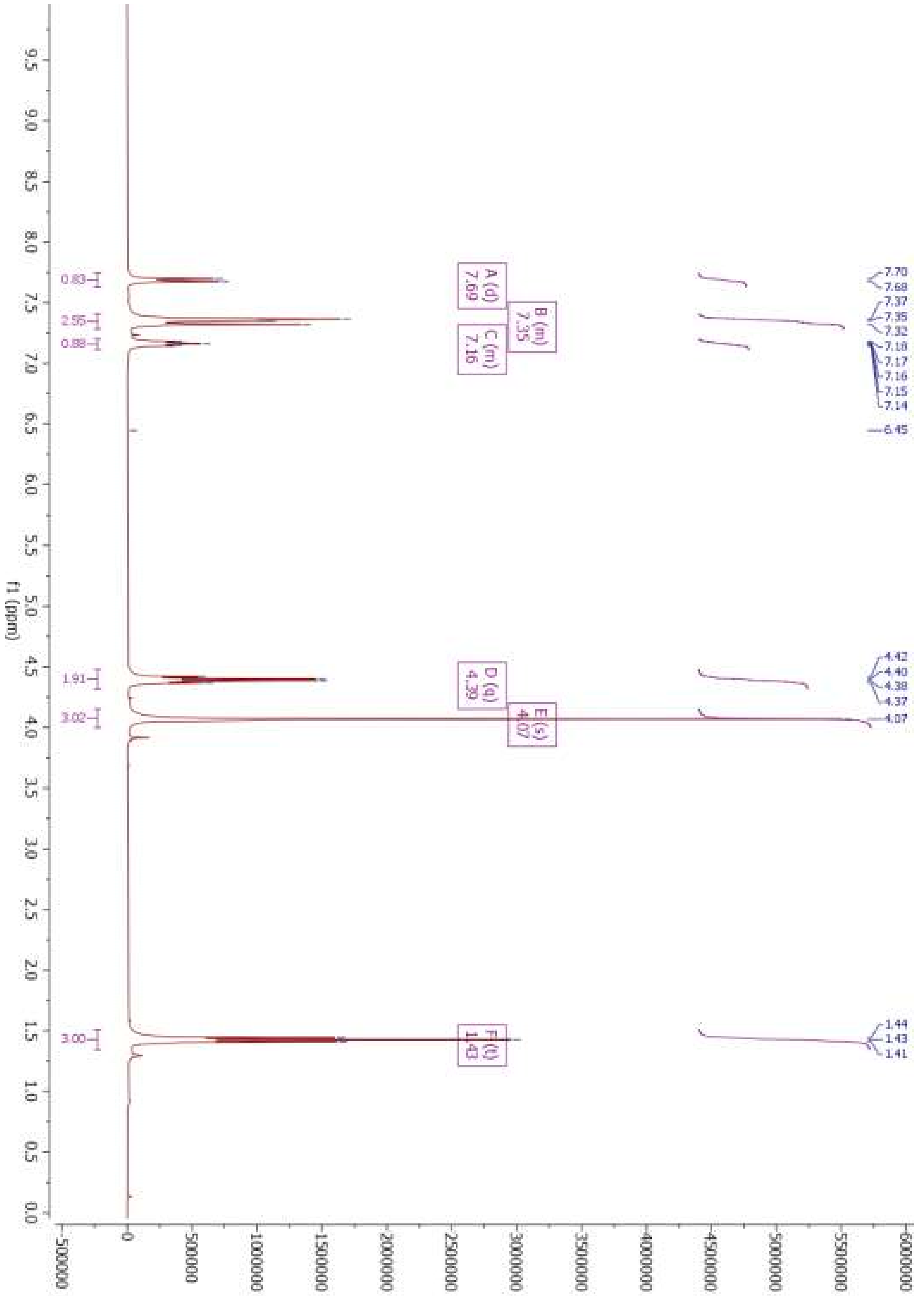

1-Methyl-1H-indole-2-carboxylic acid (9)

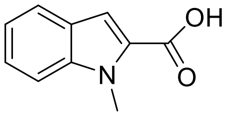

$C_{10}H_9NO_2$ **MW 175.19**

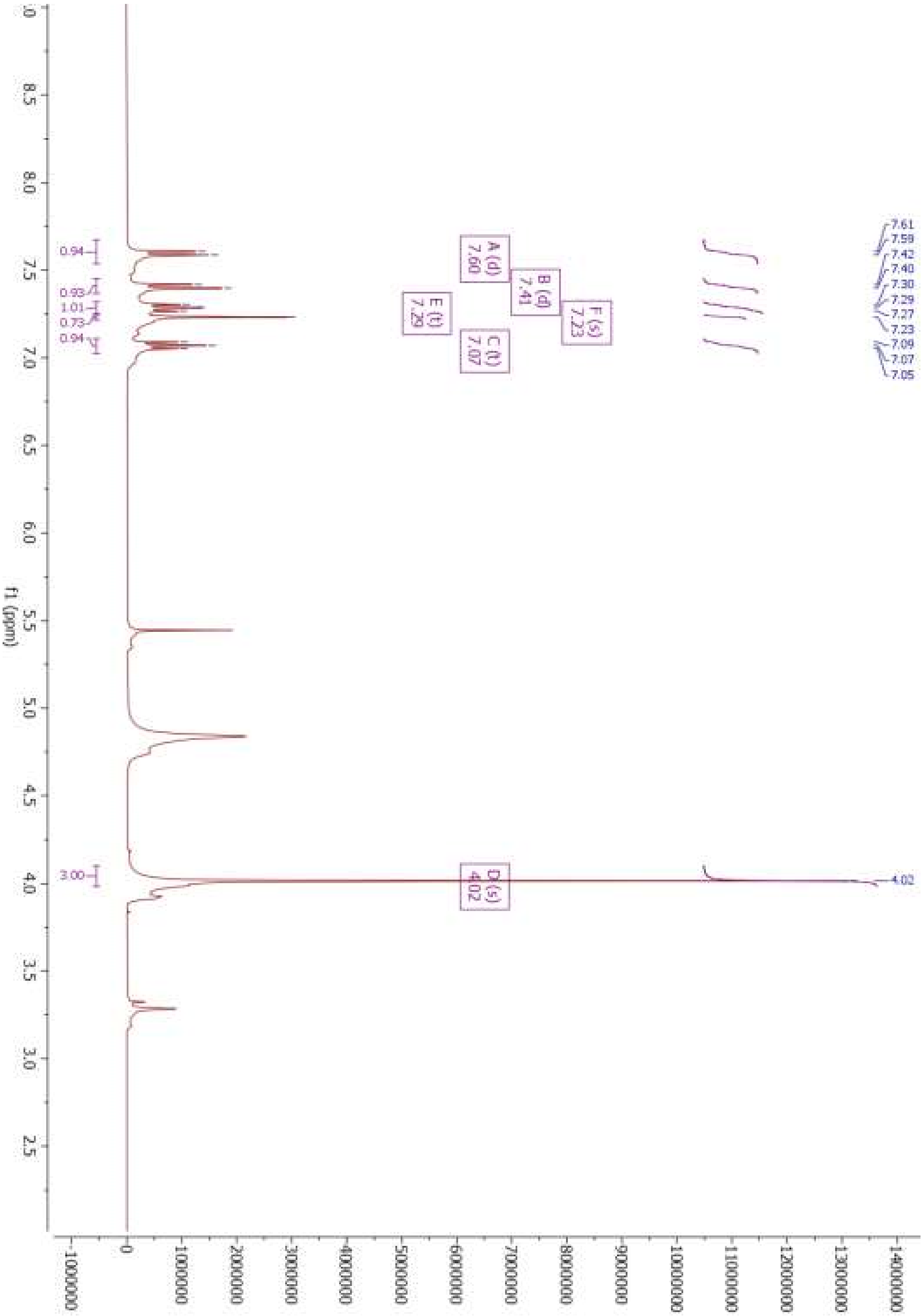

N-(4-Hydroxyphenyl)-1H-indole-2-carboxamide (5a)

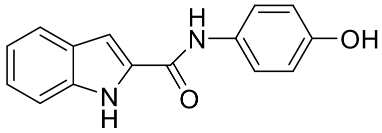

$C_{15}H_{12}N_2O_2$ **MW 252.27**

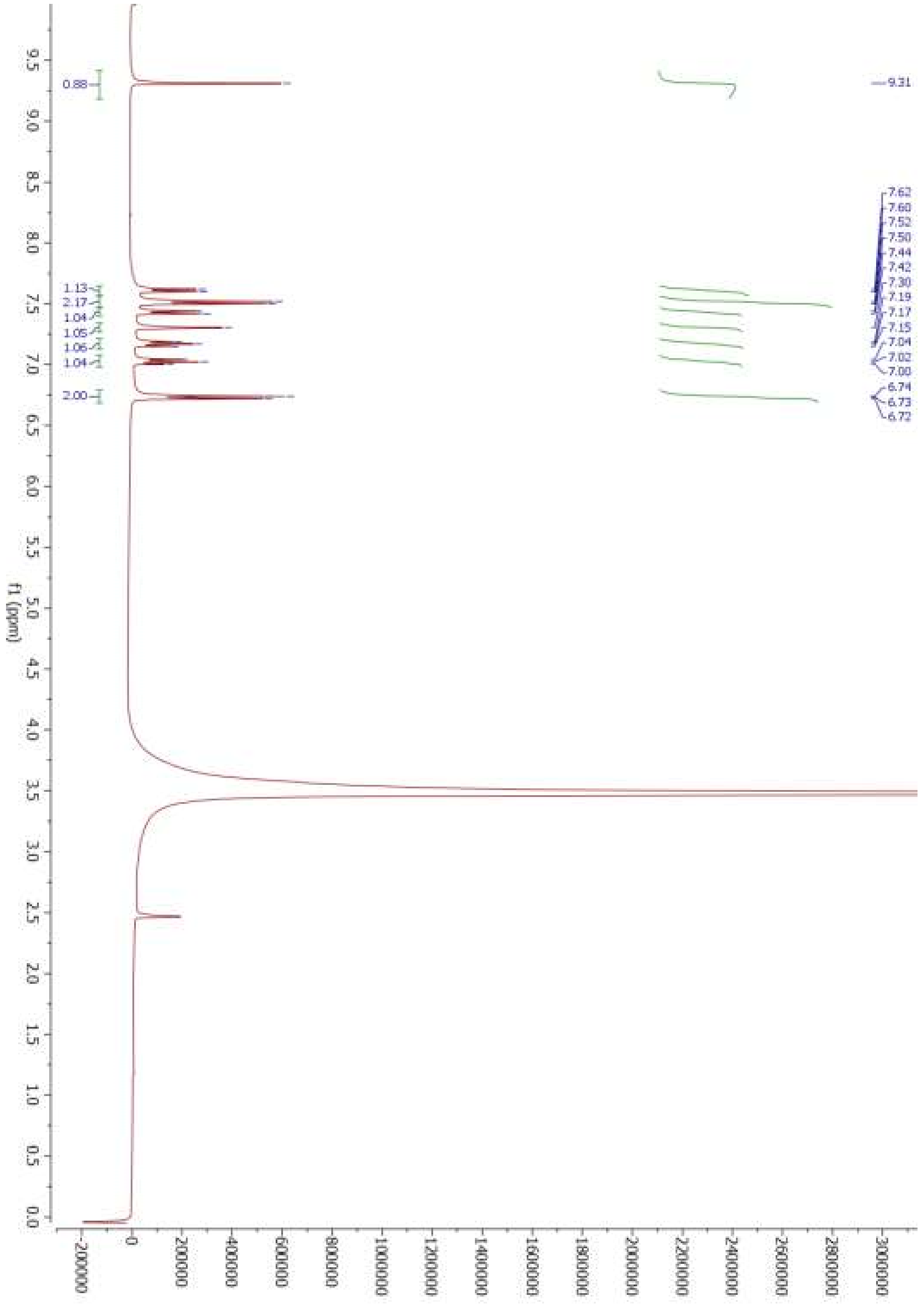

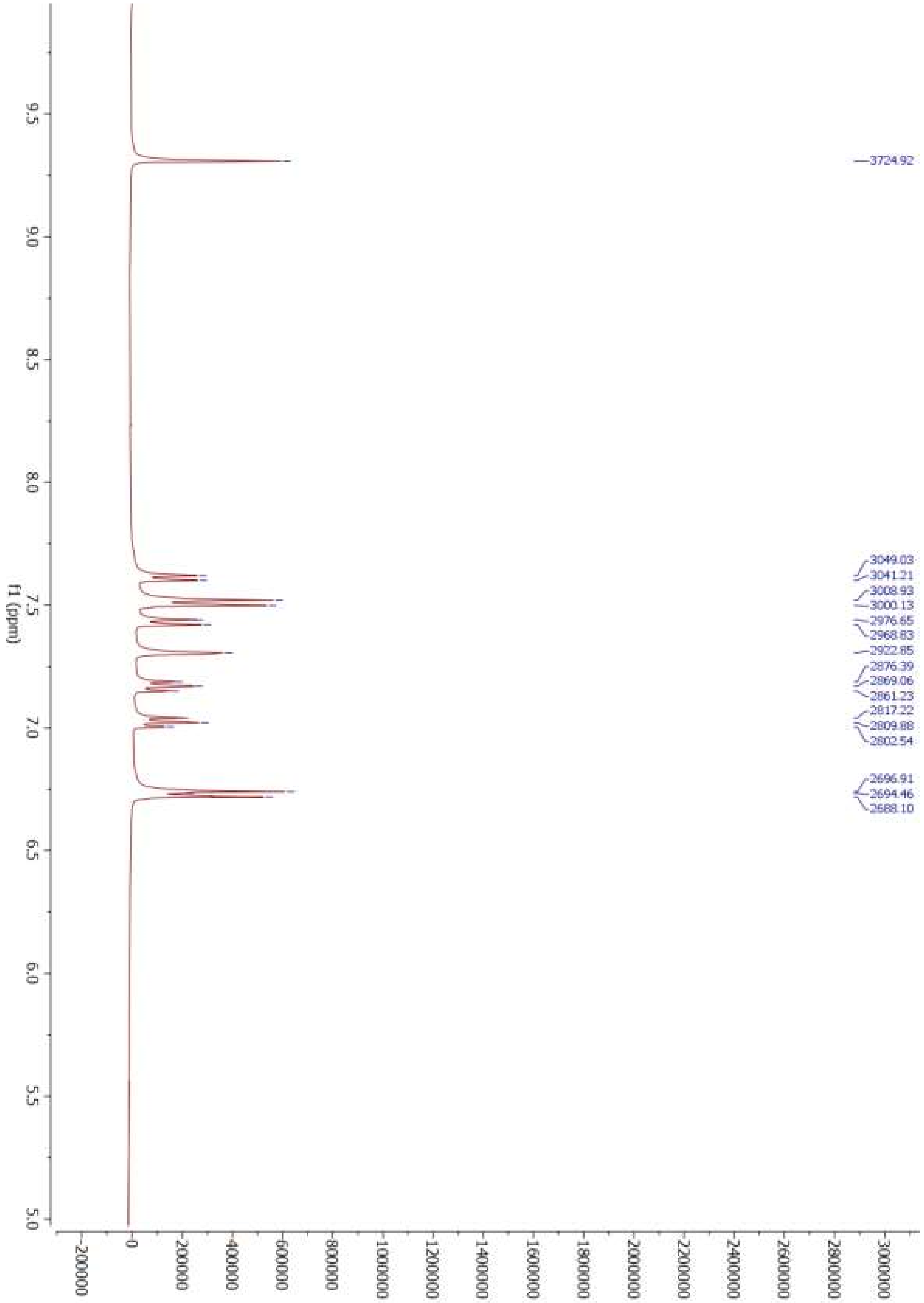

f1 (ppm)
3724.92
3049.03
3041.21
3008.93
3000.13
2976.65
2968.83
2922.85
2876.39
2869.06
2861.23
2817.22
2809.88
2802.54
2696.91
2694.46
2688.10

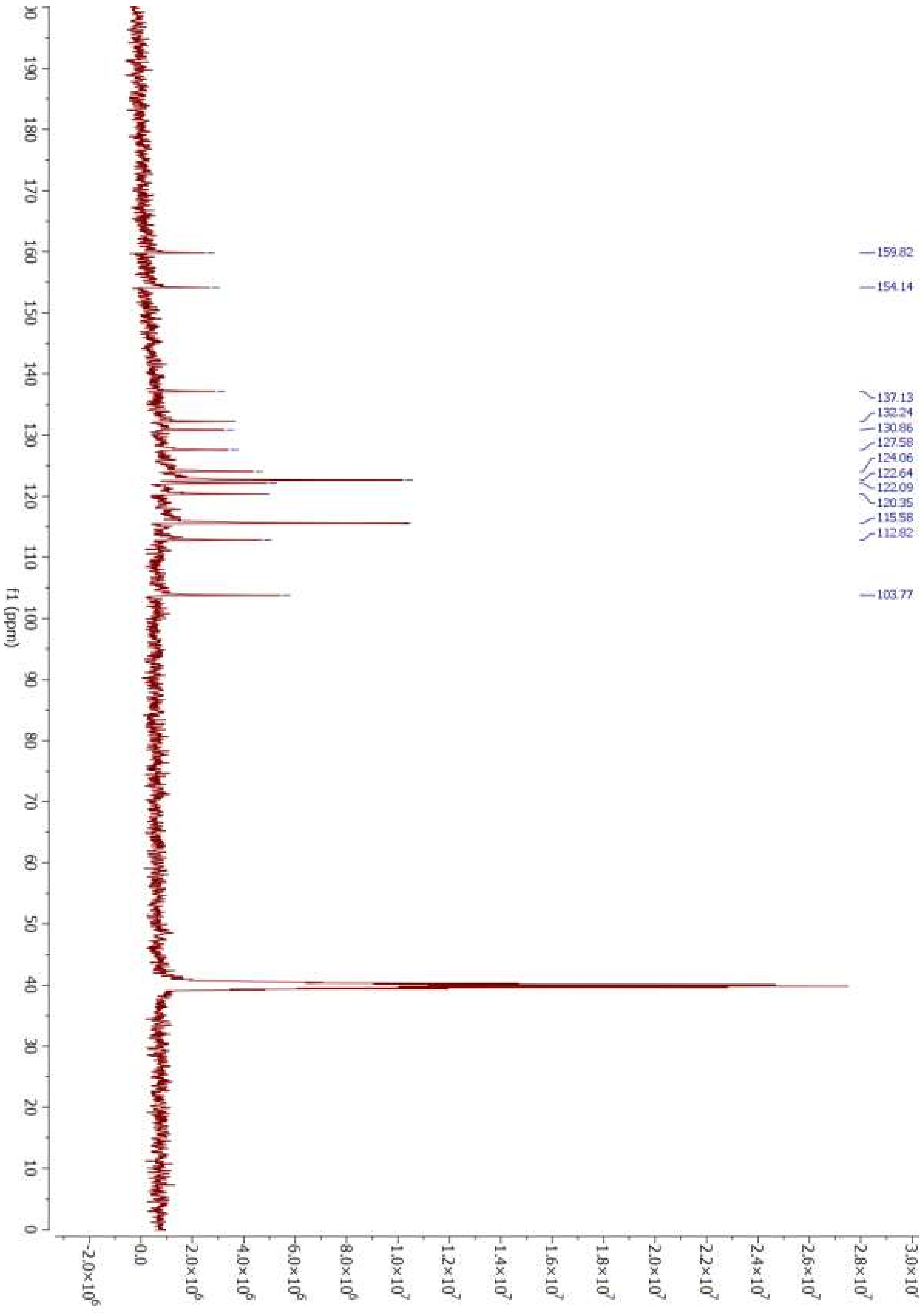
159.82
154.14
137.13
132.24
130.86
127.58
124.06
122.64
122.09
120.35
115.58
112.82
103.77
f1 (ppm)

Sample Report (continued):

Sample 52 Vial 1:F,3 ID File I-0416615-001 Date 29-Aug-2018 Time 16:45:42 Description Met Gen

3: UV Detector: TIC 2.922e+2
Range: 2.922e+2

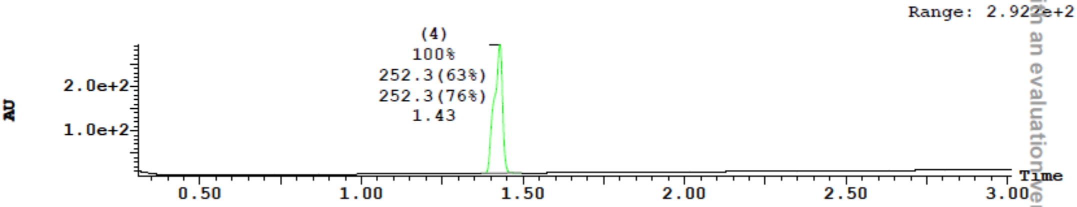

Peak Number	Time	Area %Total	Mass Found
4	1.43	100.00	252.27, 252.27

1: MS ES+ :TIC 6.3e008

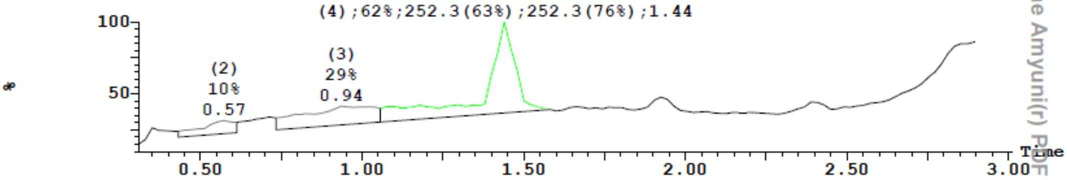

2: MS ES- :TIC 3.3e008

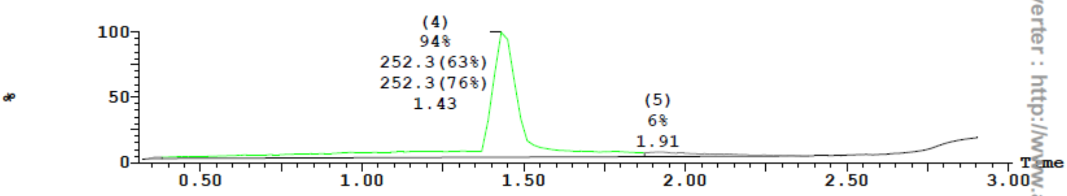

Peak ID	Time	Mass Found
4	1.44	253.27

4:(Time: 1.43) 1:MS ES+
1.0e007

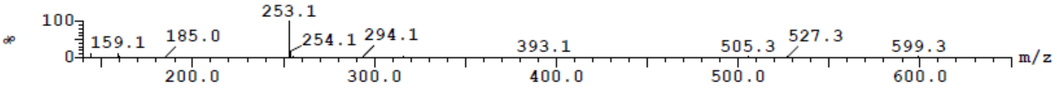

Sample Report (continued):

Peak ID	Time	Mass Found
4	1.44	251.27

4:(Time: 1.43) 2:MS ES-
8.7e006

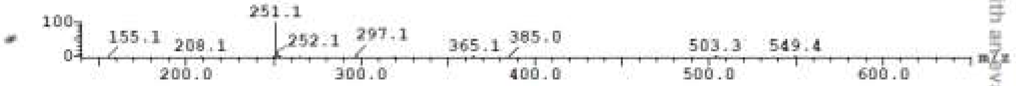

This file was generated with an evaluation version of the Amyuni(r) PDF Converter : http://www.amyuni.com

N-Benzyl-1H-indole-2-carboxamide (5b)

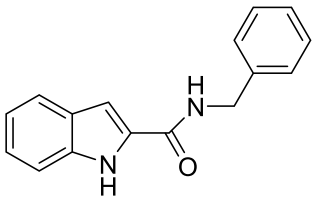

$C_{16}H_{14}N_2O$ **MW 250.30**

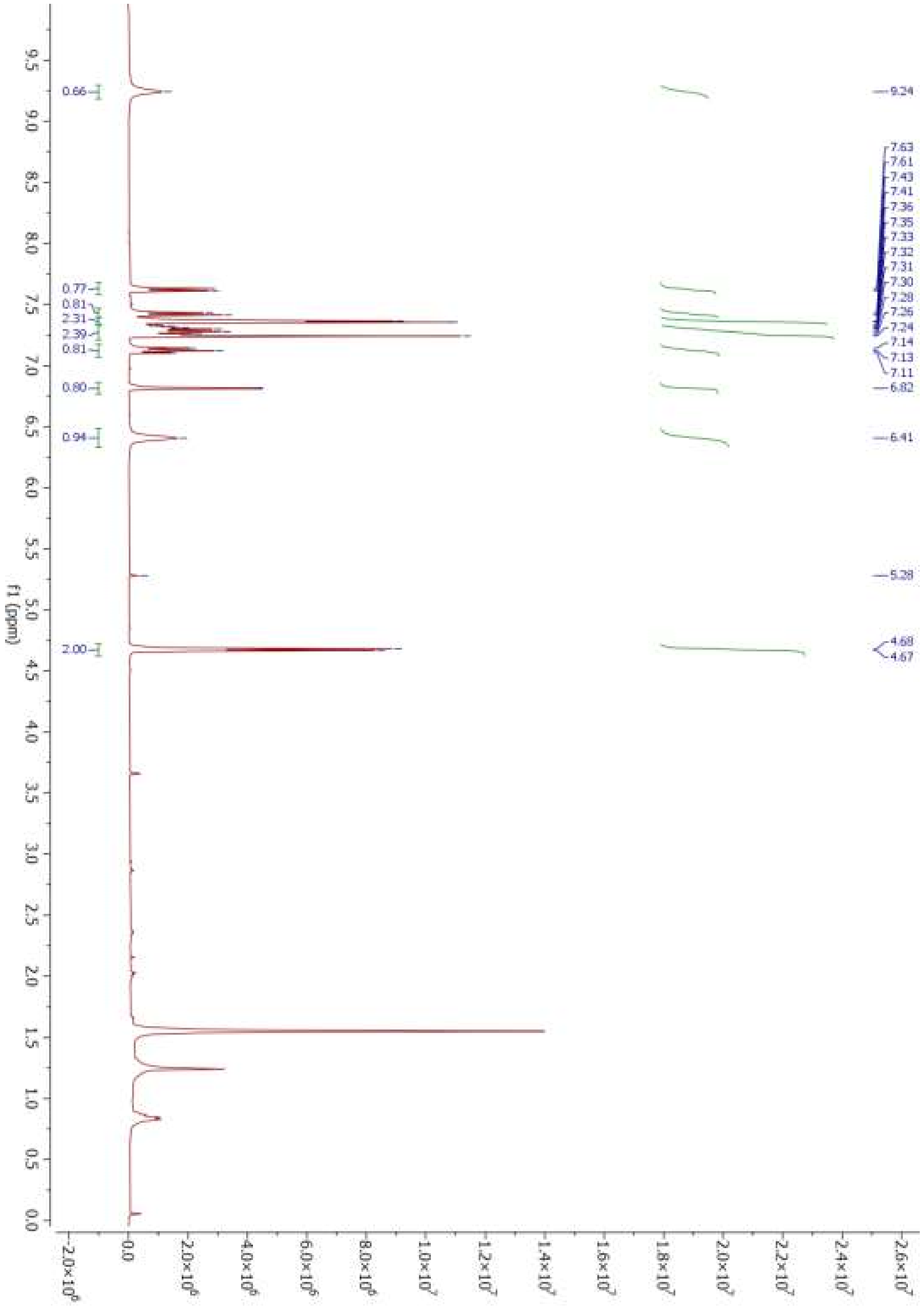

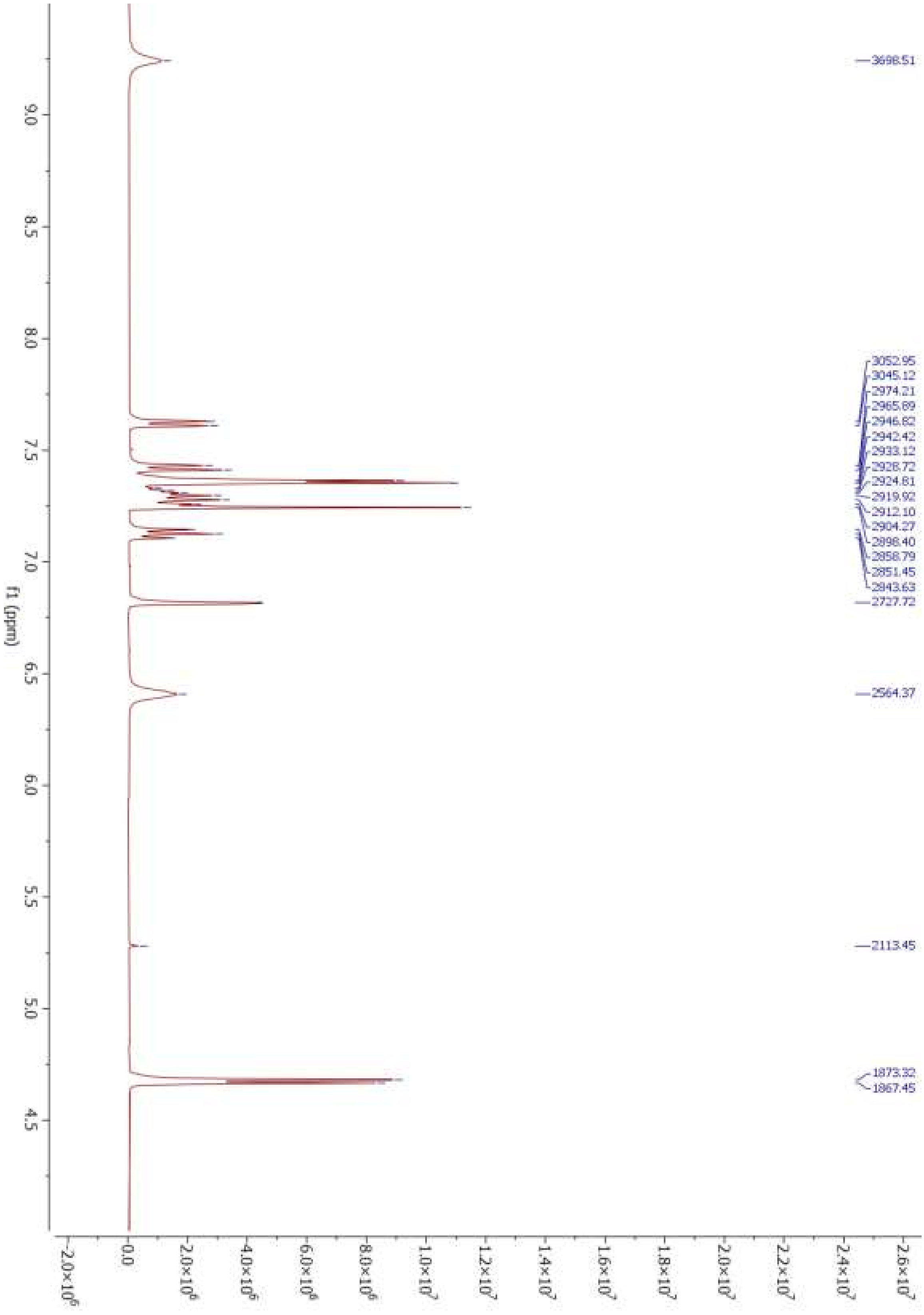
3698.51
3052.95
3045.12
2974.21
2965.89
2946.82
2942.42
2933.12
2928.72
2924.81
2919.92
2912.10
2904.27
2898.40
2858.79
2851.45
2843.63
2727.72
2564.37
2113.45
1873.32
1867.45
9.0
8.5
8.0
7.5
7.0
6.5
6.0
5.5
5.0
4.5
f1 (ppm)
-2.0×10^6
0.0
2.0×10^6
4.0×10^6
6.0×10^6
8.0×10^6
1.0×10^7
1.2×10^7
1.4×10^7
1.6×10^7
1.8×10^7
2.0×10^7
2.2×10^7
2.4×10^7
2.6×10^7

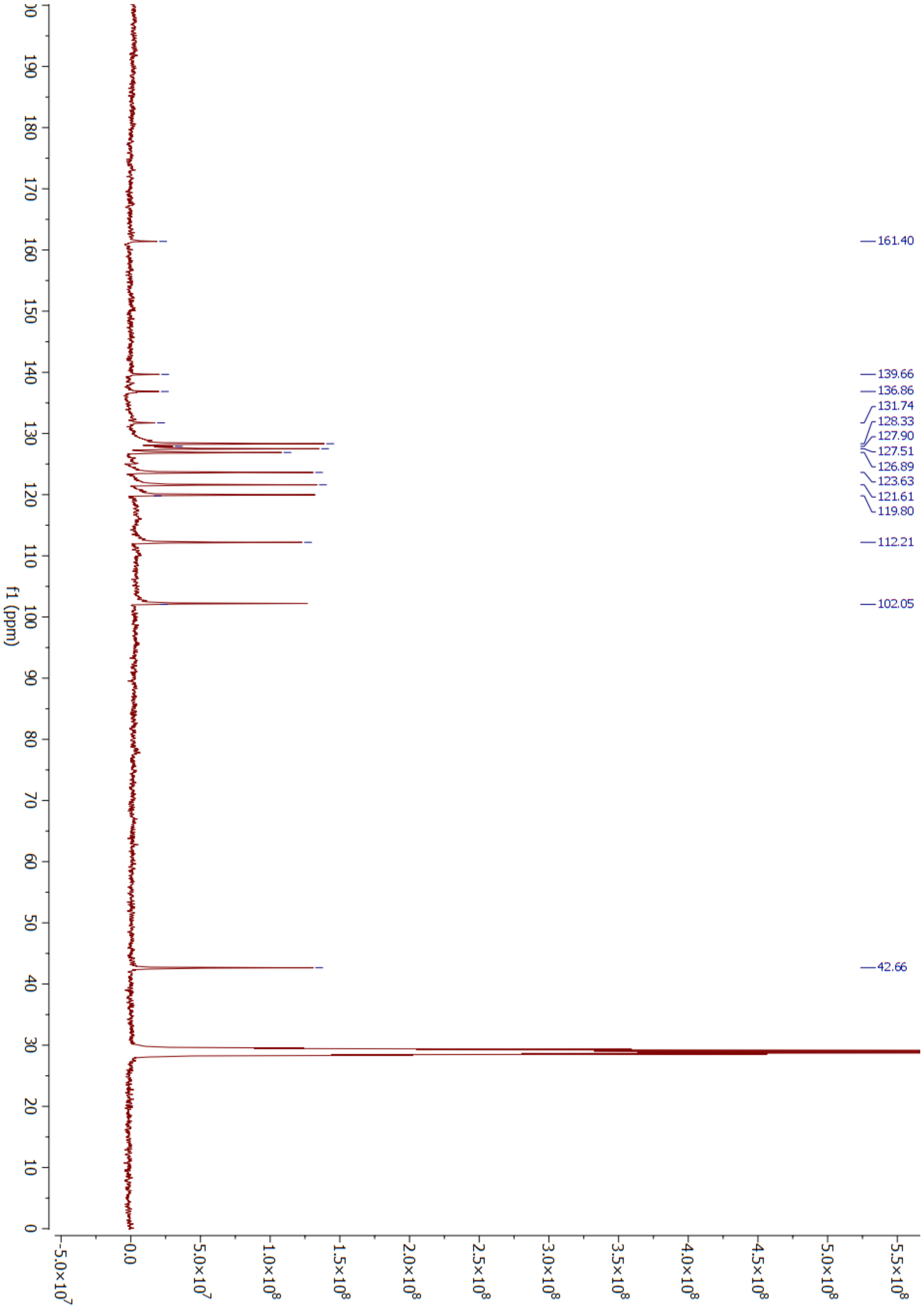
161.40
139.66
136.86
131.74
128.33
127.90
127.51
126.89
123.63
121.61
119.80
112.21
102.05
42.66
f1 (ppm)

Sample Report (continued):

Sample 62 Vial 1:G,3 ID File I-0254678-002 Date 29-Aug-2018 Time 17:30:00 Description Met Gen

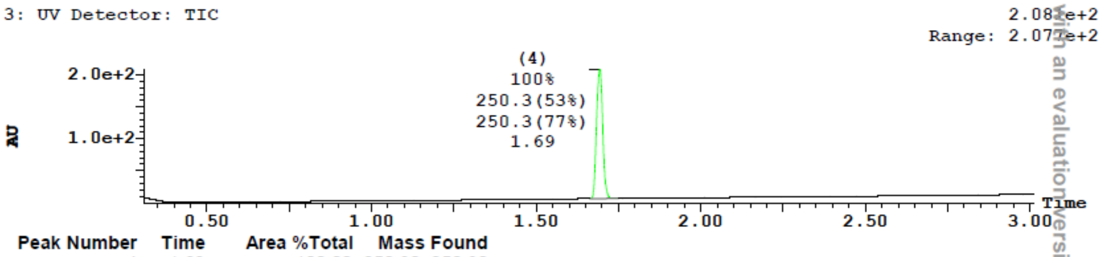

Peak Number	Time	Area %Total	Mass Found
4	1.69	100.00	250.30, 250.30

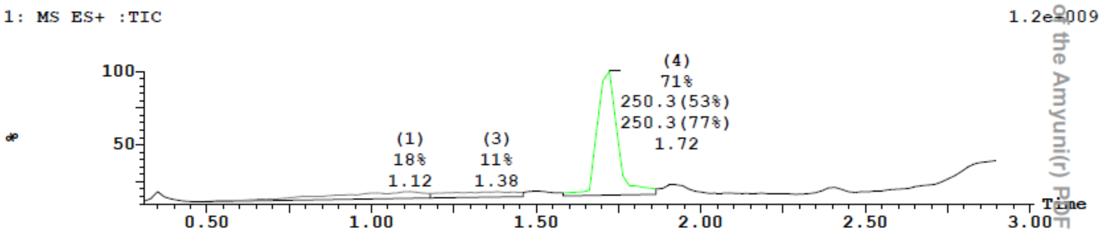

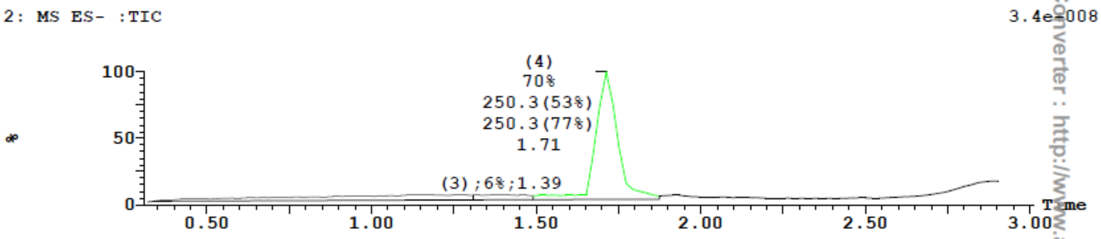

Peak ID	Time	Mass Found
4	1.72	251.30

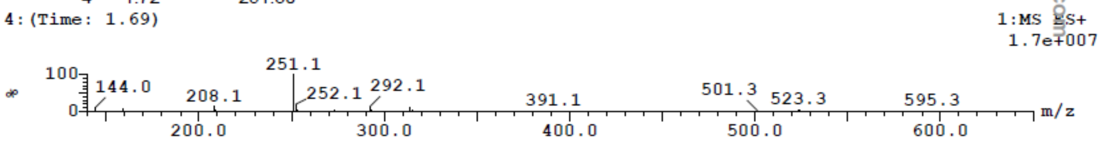

Sample Report (continued):

Peak ID	Time	Mass Found
4	1.72	249.30

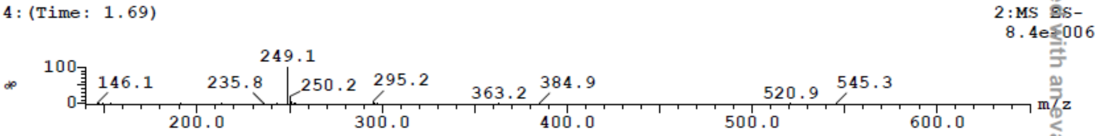

This file was generated with an evaluation version of the Amyuni(r) PDF Converter : http://www.amyuni.com
This file was generated with an eval

_N_-(4-Hydroxybenzyl)-1_H_-indole-2-carboxamide (5c)

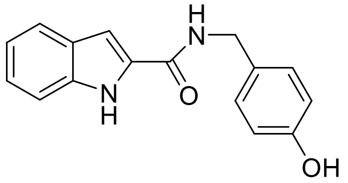

$C_{16}H_{14}N_2O_2$ **MW 266.30**

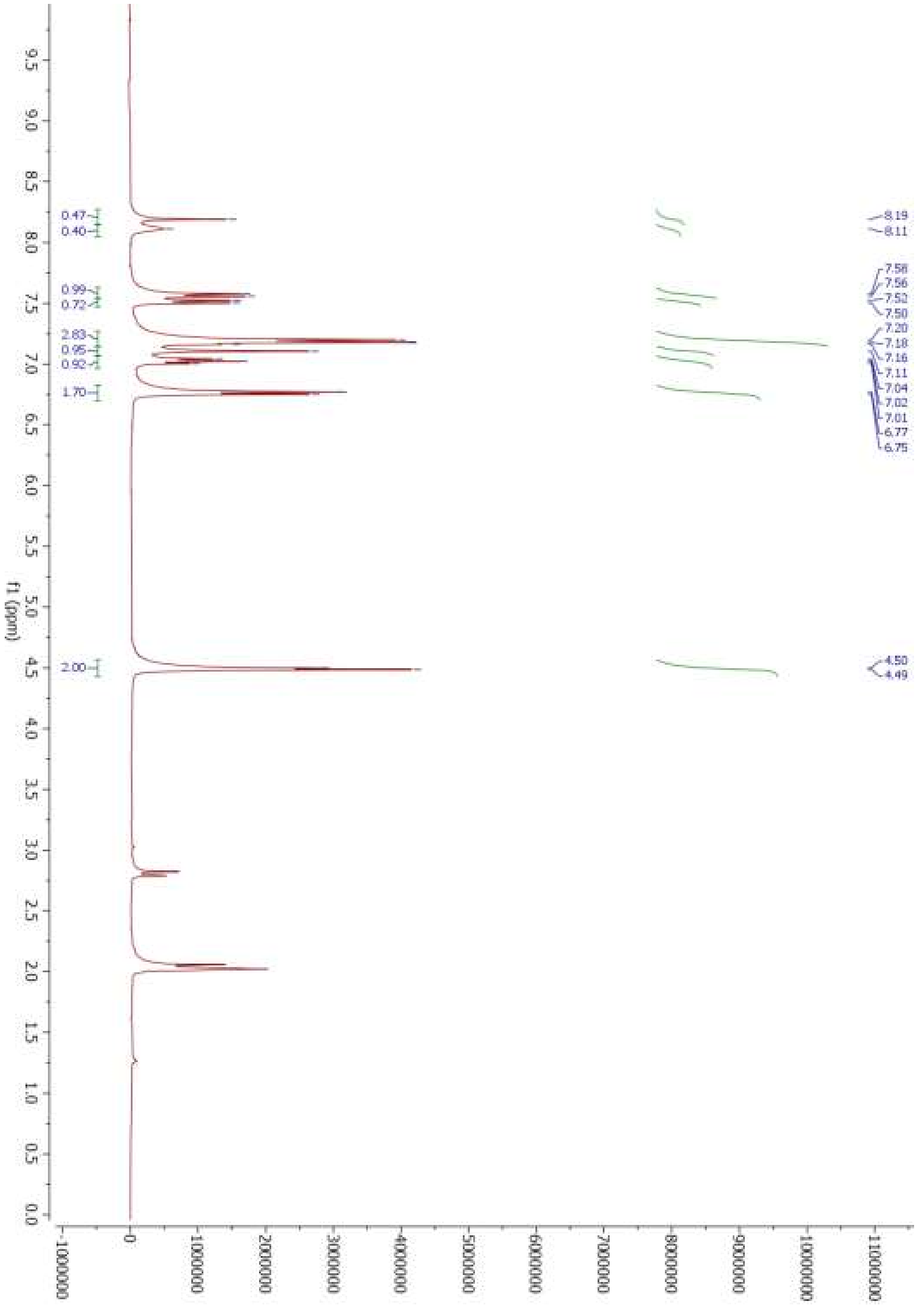

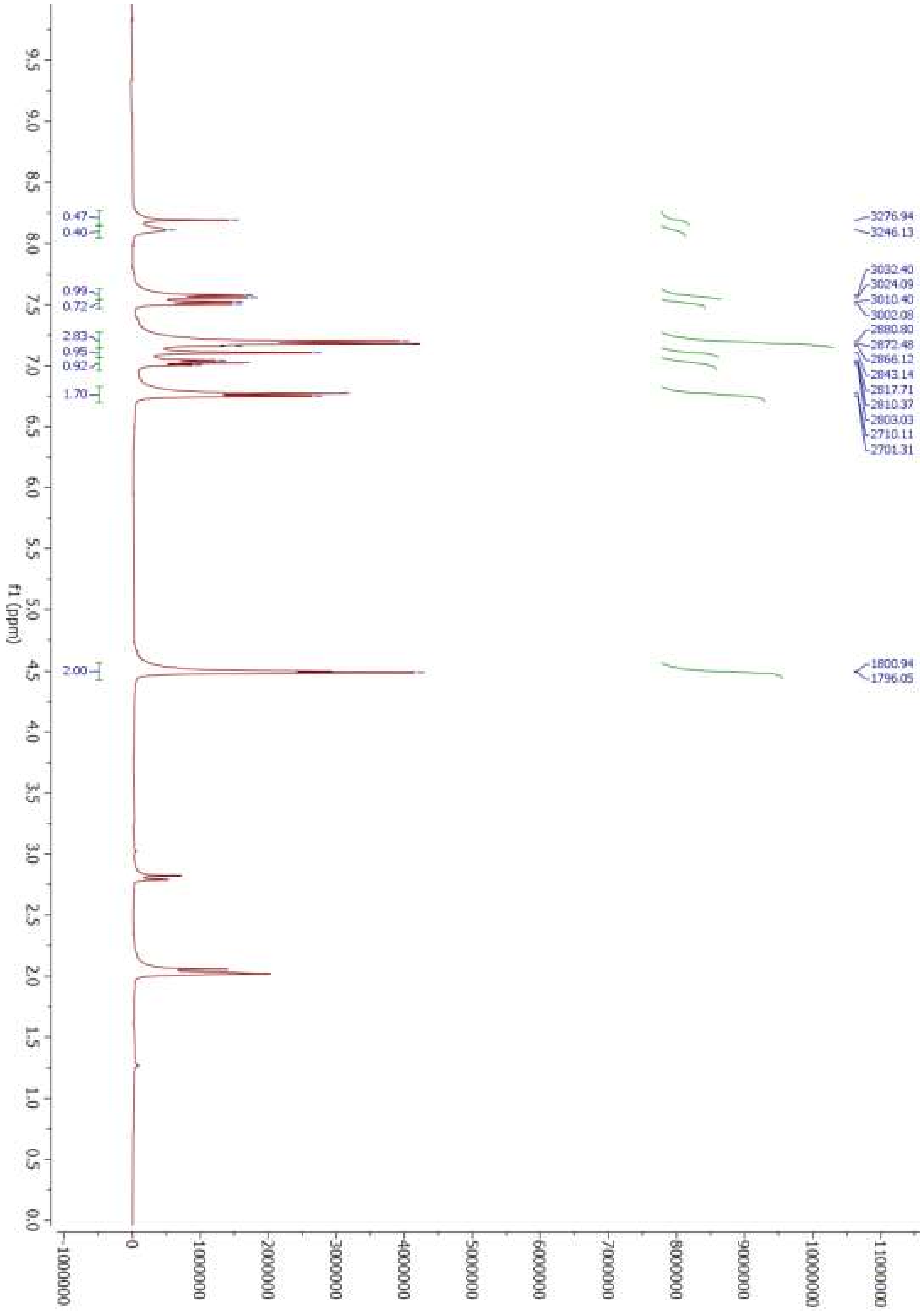
3276.94
3246.13
3032.40
3024.09
3010.40
3002.08
2880.80
2872.48
2866.12
2843.14
2817.71
2810.37
2803.03
2710.11
2701.31
1800.94
1796.05
0.47
0.40
0.99
0.72
2.83
0.95
0.92
1.70
2.00
f1 (ppm)

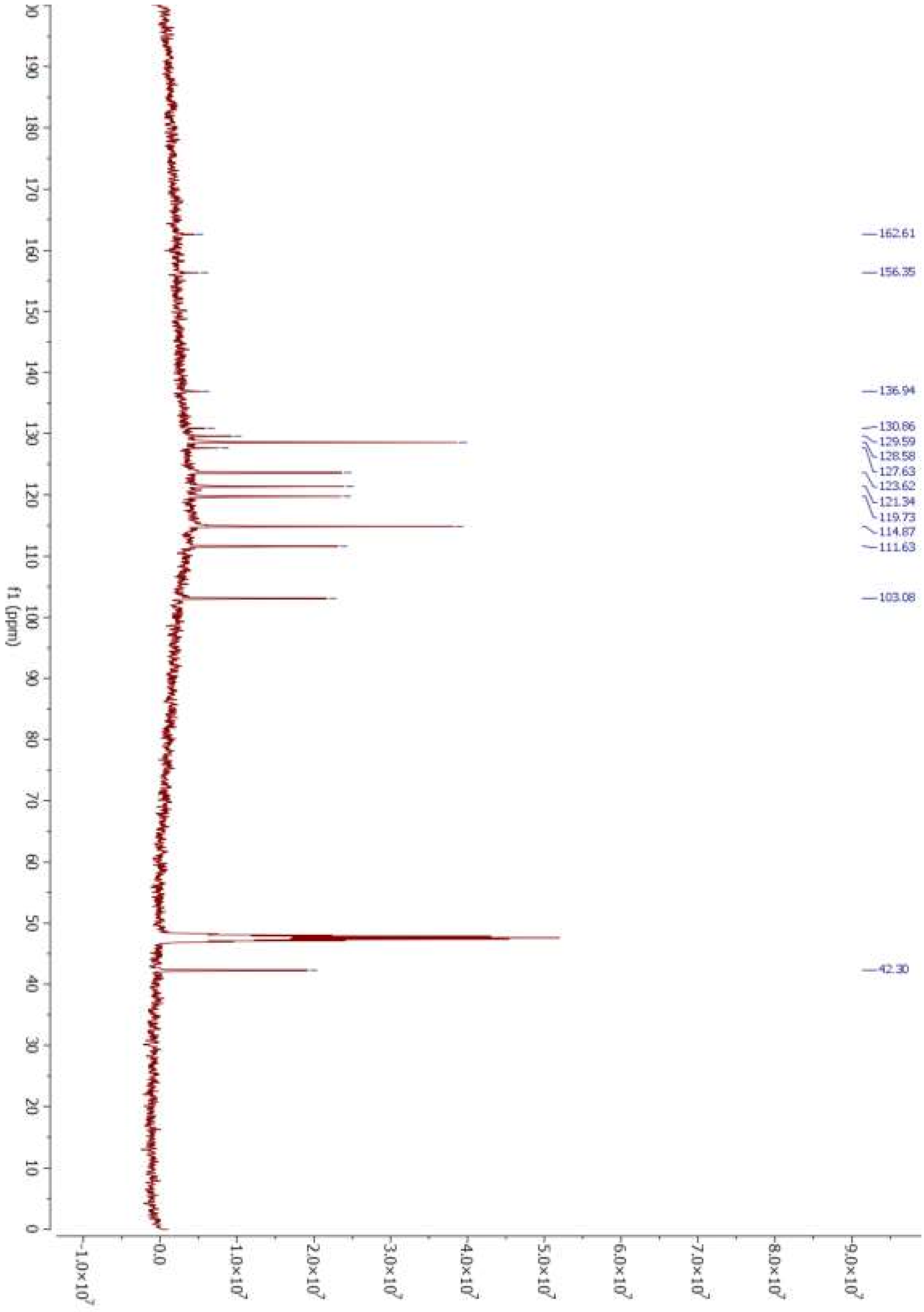

162.61
156.35
136.94
130.86
129.59
128.58
127.63
123.62
121.34
119.73
114.87
111.63
103.08
42.30
f1 (ppm)

This file was generated with an evaluation version of the Amyuni(r) PDF Converter : http://www.amyuni.com

Sample Report (continued):

Sample 50 Vial 1:E,11 ID File I-0416614-001 Date 29-Aug-2018 Time 16:36:51 Description Met Gen

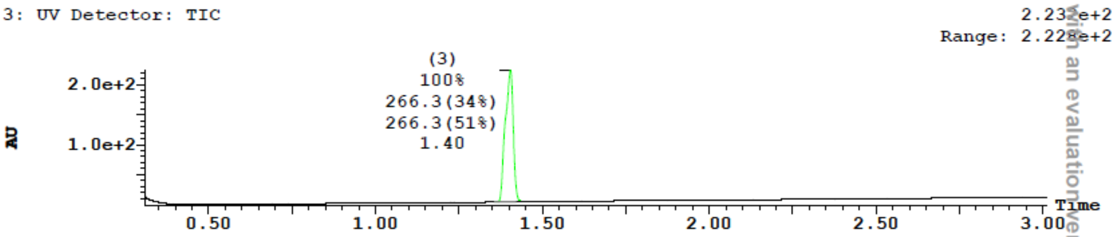

Peak Number	Time	Area %Total	Mass Found
3	1.40	100.00	266.29, 266.29

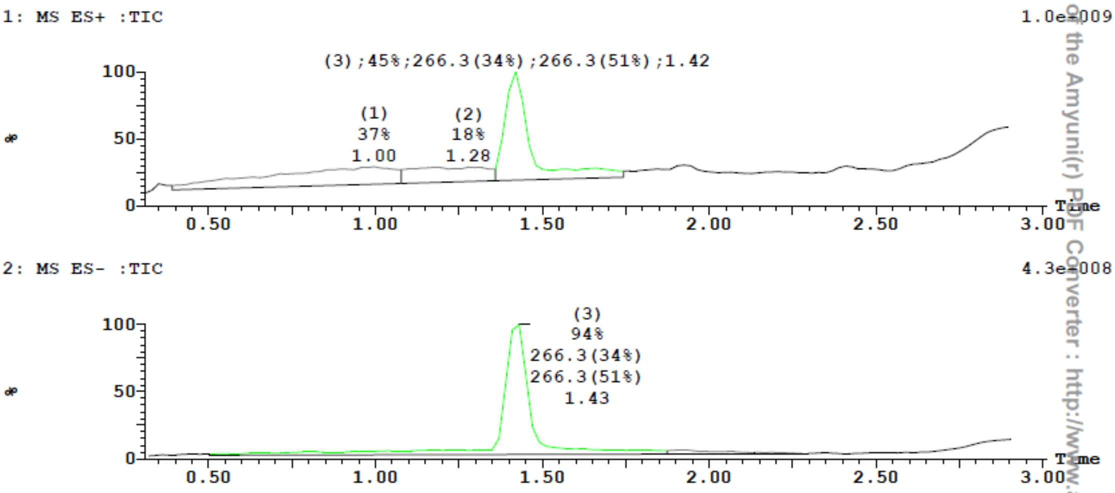

Peak ID	Time	Mass Found
3	1.42	267.29

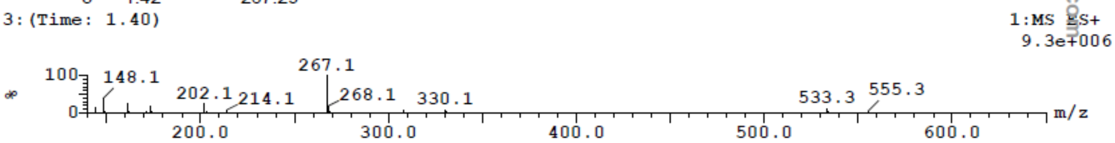

Sample Report (continued):

Peak ID	Time	Mass Found
3	1.42	265.29

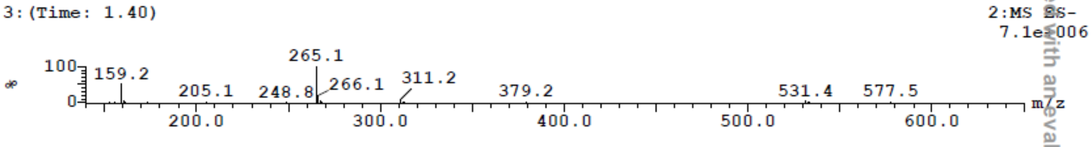

N-(4-Methoxybenzyl)-1H-indole-2-carboxamide (5d)

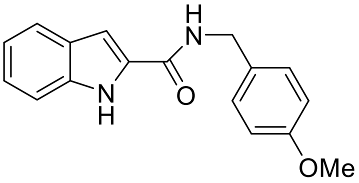

$C_{17}H_{16}N_2O_2$ **MW 280.33**

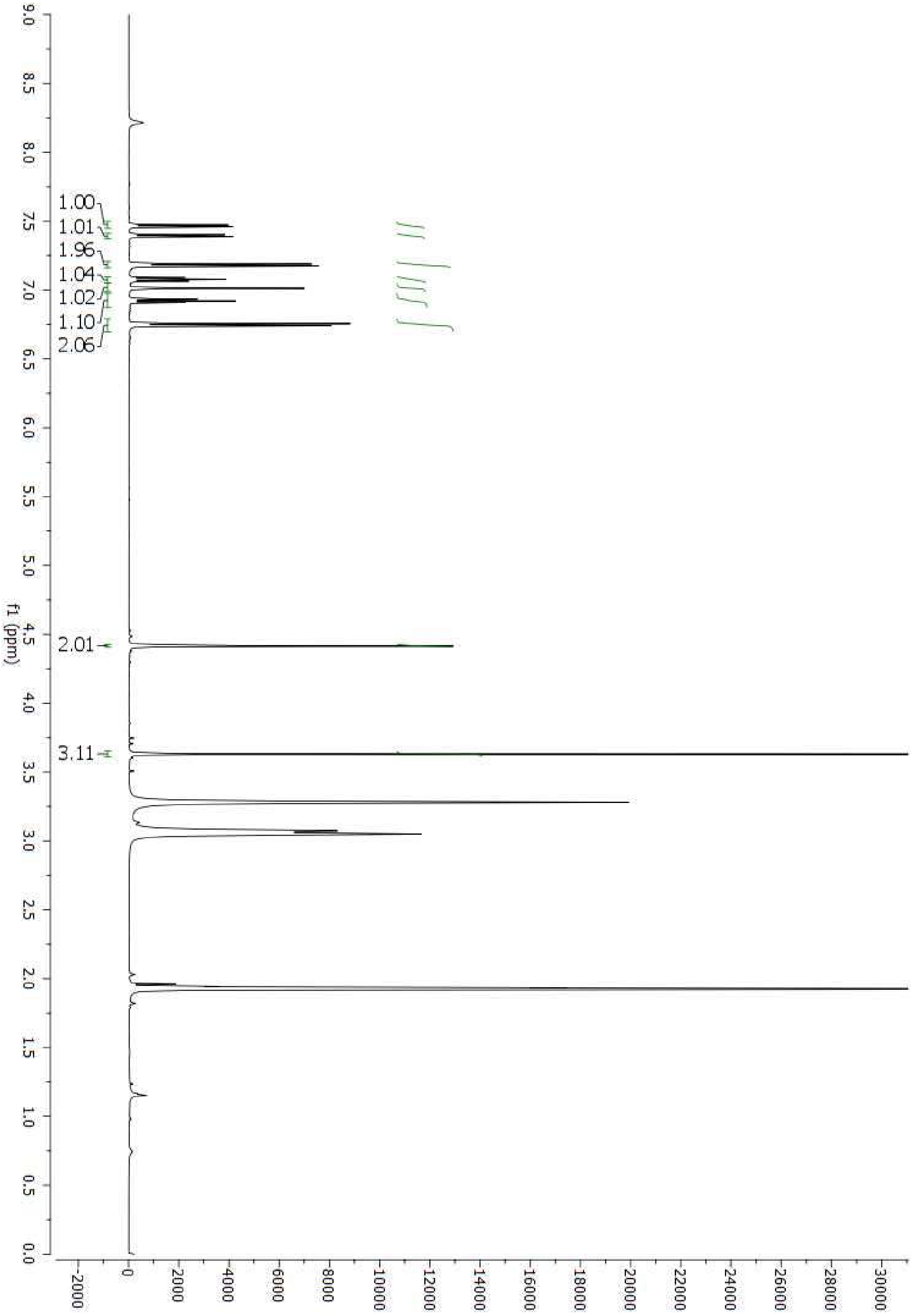

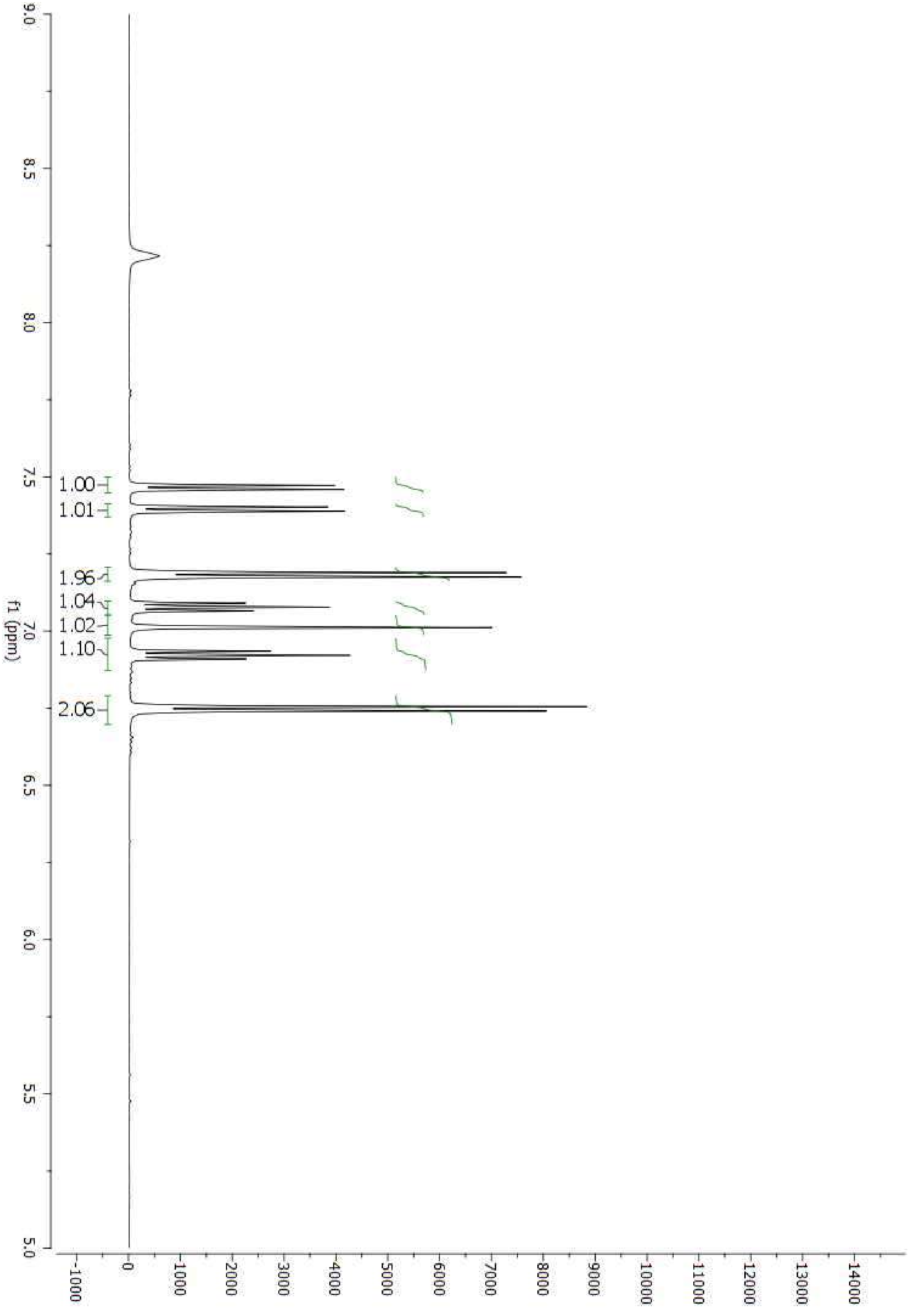
f1 (ppm)
1.00
1.01
1.96
1.04
1.02
1.10
2.06

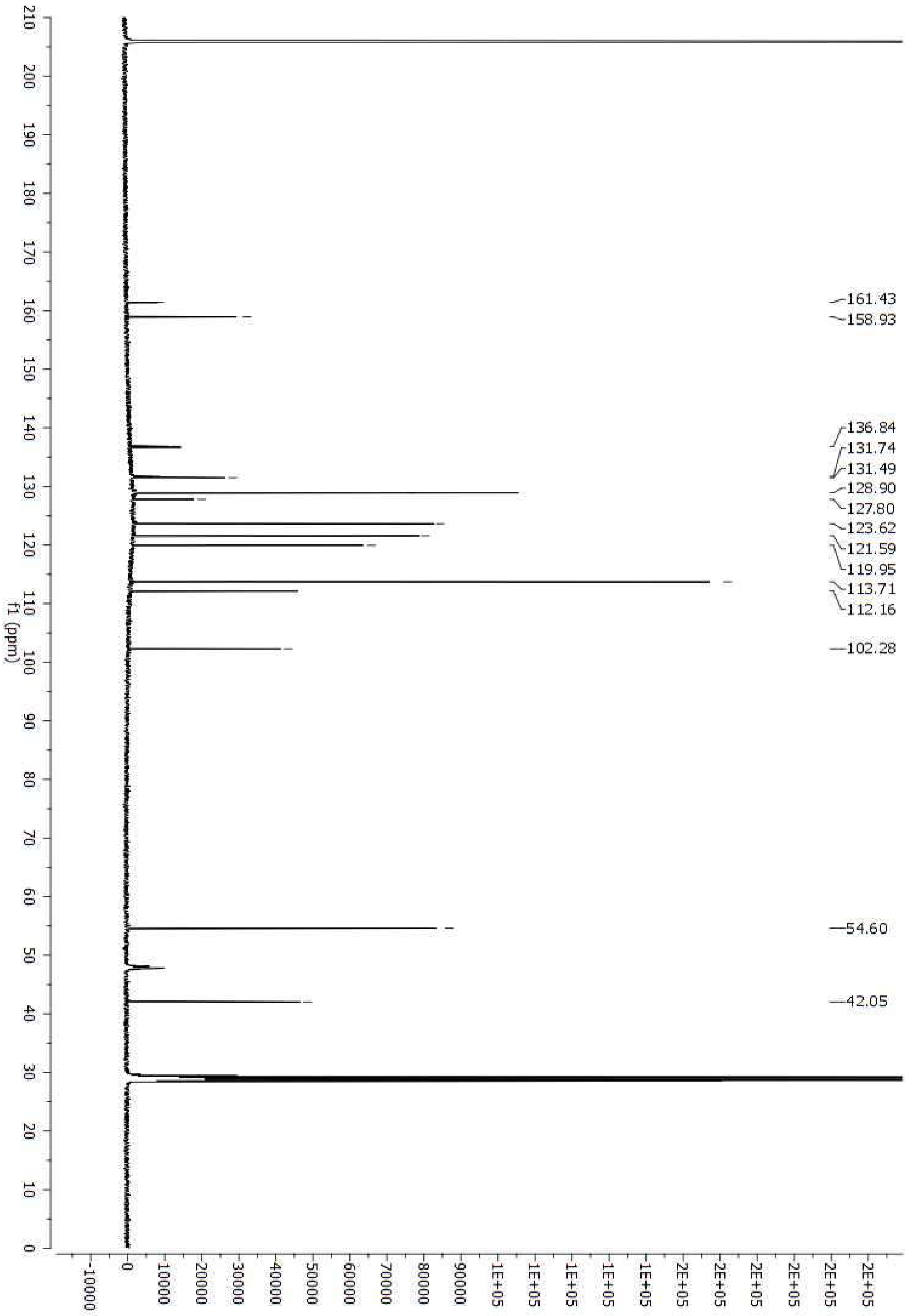

161.43
158.93
136.84
131.74
131.49
128.90
127.80
123.62
121.59
119.95
113.71
112.16
102.28
54.60
42.05
f1 (ppm)

Openlynx Report - Page 103

Sample: 51 Vial:1:F,2 File:I-0254881-002

Date:29-Aug-2018 Time:16:41:18

Printed: Thu Aug 30 10:30:57 2018

Sample Report (continued):

Sample 51 Vial 1:F,2 ID File I-0254881-002 Date 29-Aug-2018 Time 16:41:18 Description Met Gen

3: UV Detector: TIC 2.343e+2 Range: 2.338e+2

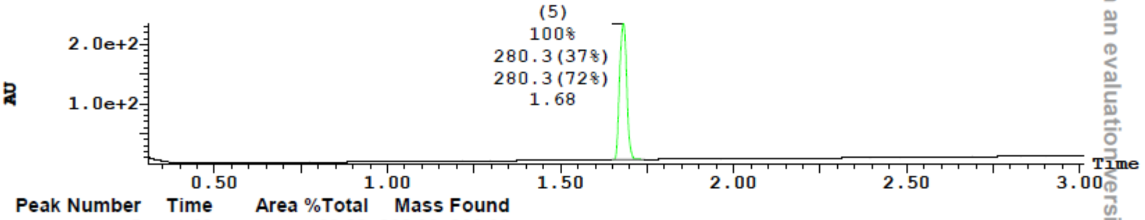

Peak Number	Time	Area %Total	Mass Found
5	1.68	100.00	280.32, 280.32

1: MS ES+ :TIC 9.9e008

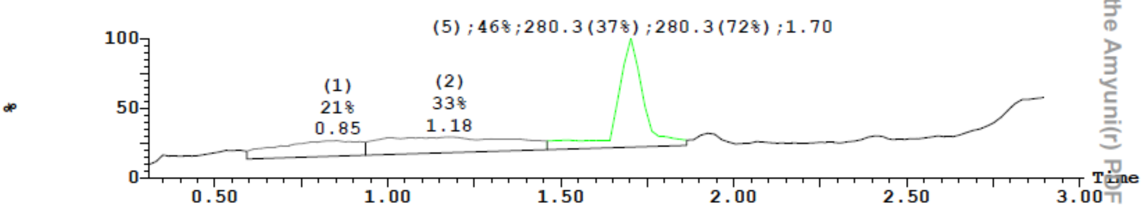

2: MS ES- :TIC 2.4e008

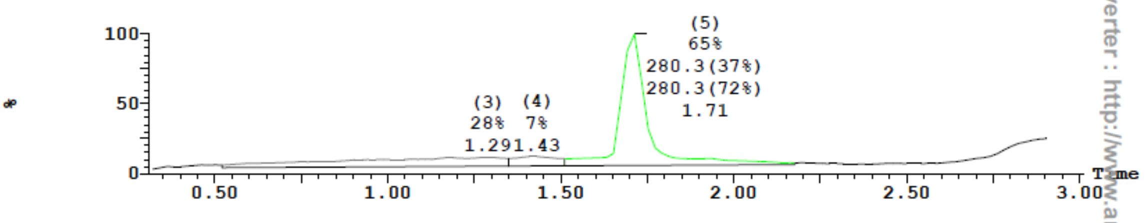

Peak ID	Time	Mass Found
5	1.70	281.32

5: (Time: 1.68) 1:MS ES+ 1.2e+007

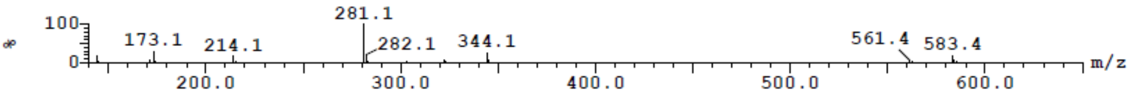

Openlynx Report - Page 104

Sample: 51 Vial:1:F,2 File:I-0254881-002

Date:29-Aug-2018 Time:16:41:18

Printed: Thu Aug 30 10:30:57 2018

Sample Report (continued):

Peak ID	Time	Mass Found
5	1.70	279.32

5: (Time: 1.68) 2:MS ES- 5.2e006

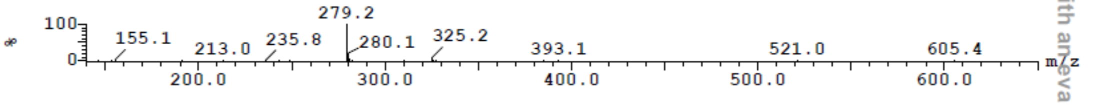

This file was generated with an evaluation version of the Amyuni(r) PDF Converter : http://www.amyuni.com

This file was generated with an eva

N-(4-Fluorobenzyl)-1*H*-indole-2-carboxamide (5e)

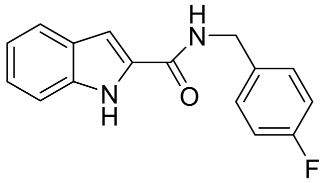

$C_{16}H_{13}FN_2O$ **MW 268.29**

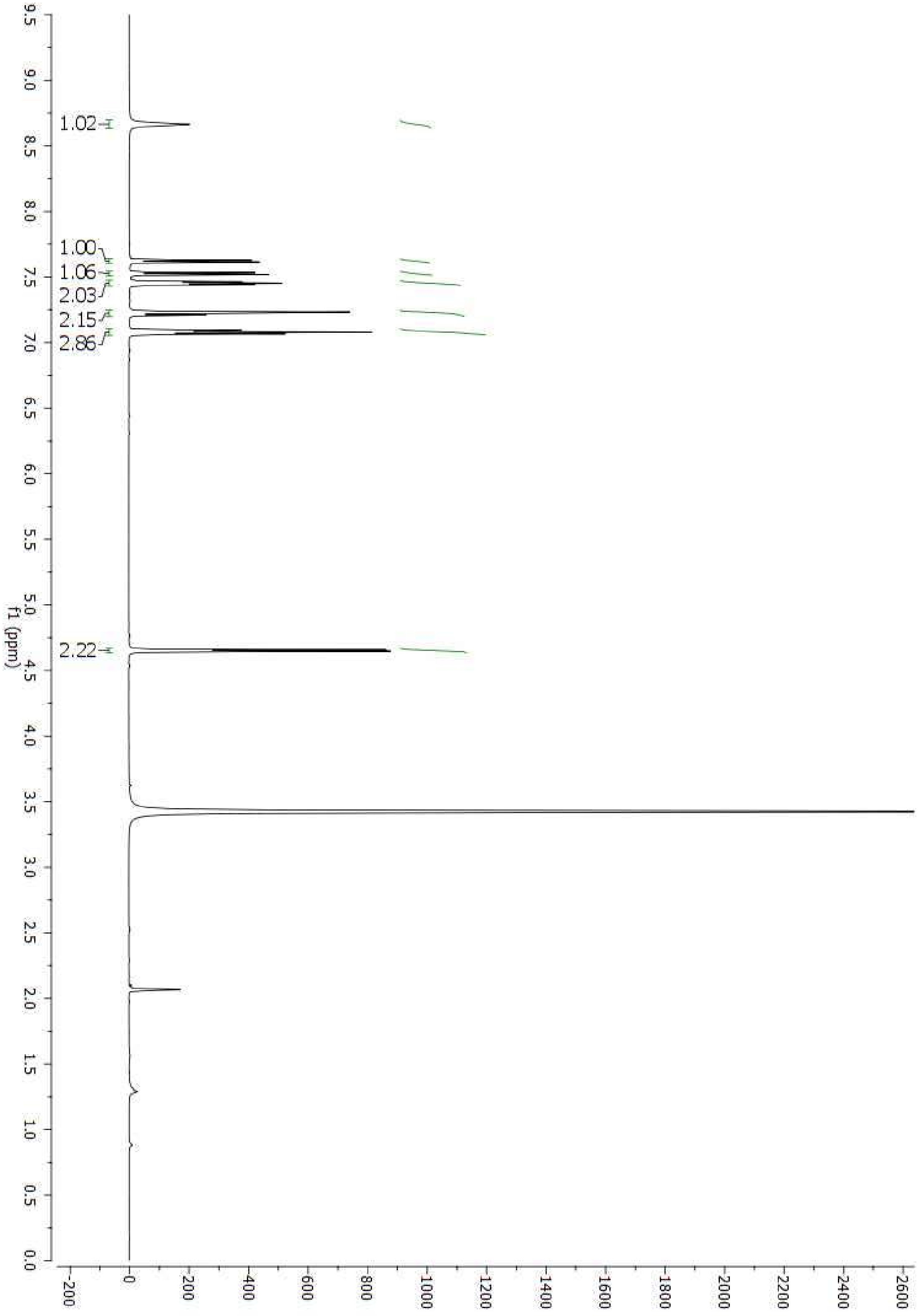

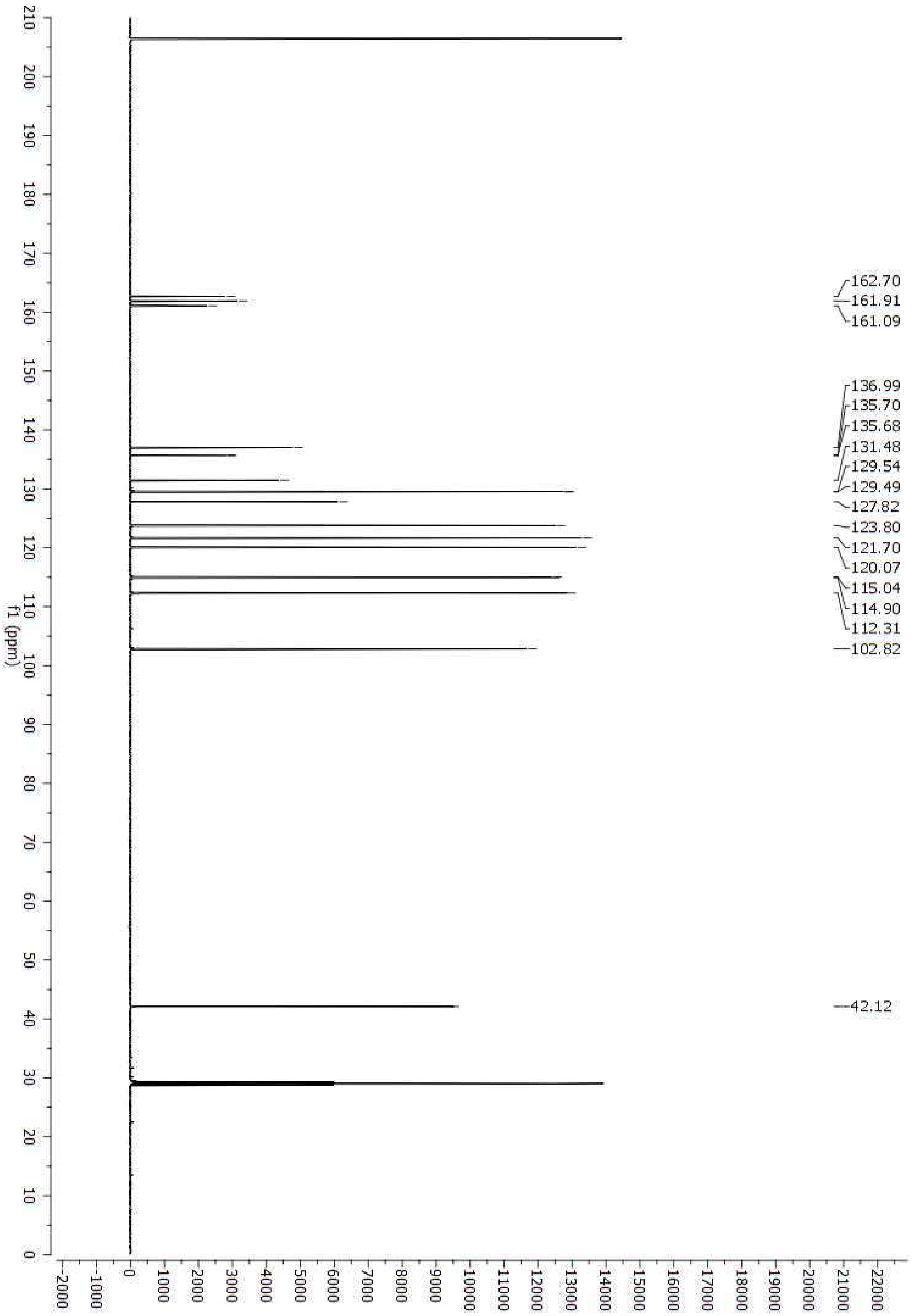

162.70
161.91
161.09
136.99
135.70
135.68
131.48
129.54
129.49
127.82
123.80
121.70
120.07
115.04
114.90
112.31
102.82
42.12
f1 (ppm)

Analysis Info

Method	MS_LC_POS_TimsOFF.m
Sample Name	MSP137

Acquisition Parameter

Source Type	ESI	Ion Polarity	Positive	Set Nebulizer	2.2 Bar
Focus	Active	Set Capillary	4000 V	Set Dry Heater	220 °C
Scan Begin	50 m/z	Set End Plate Offset	-500 V	Set Dry Gas	10.0 l/min
Scan End	2500 m/z	Set Collision Cell RF	1800.0 Vpp	Set Divert Valve	Waste

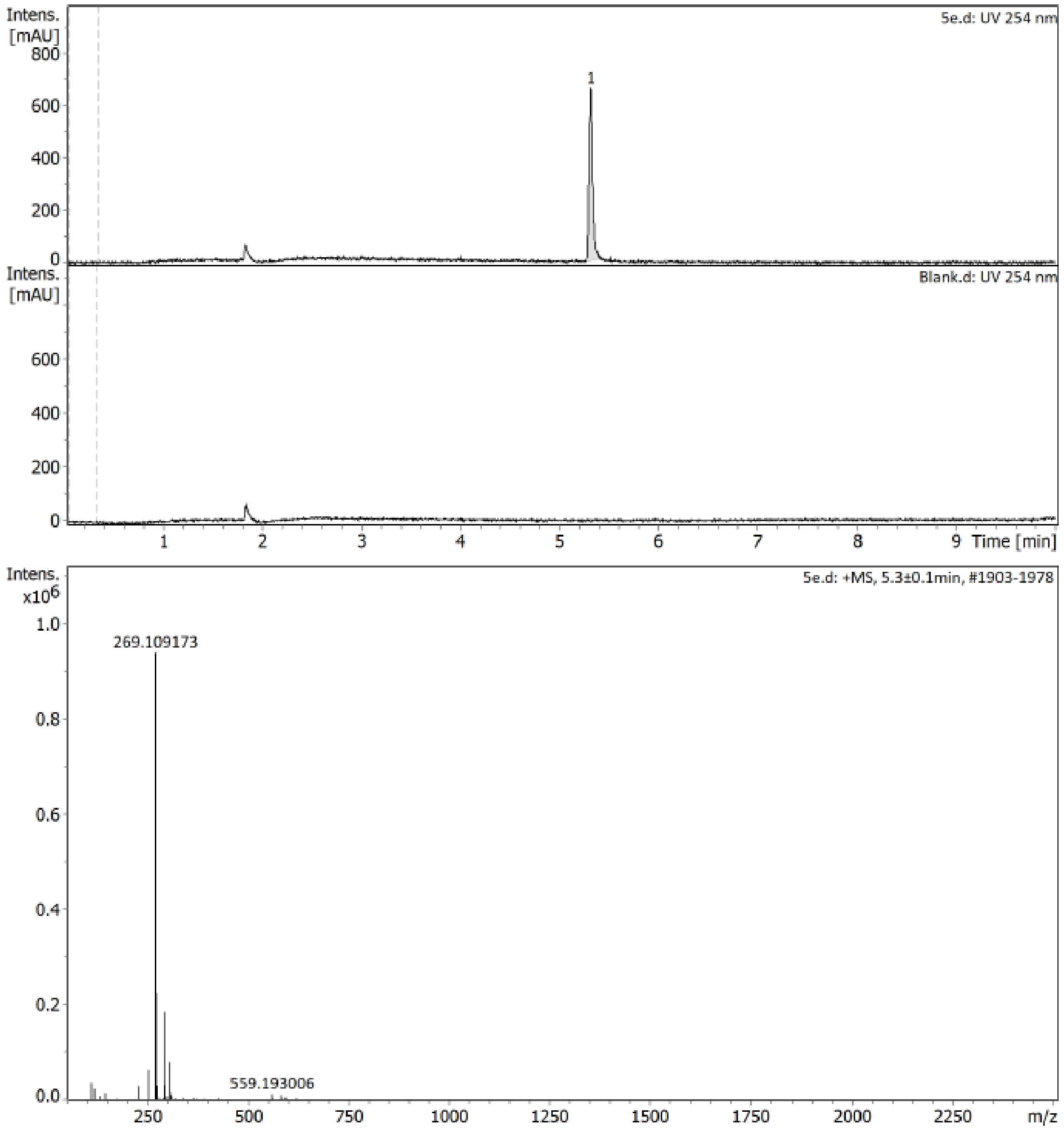

#	RT [min]	Area	Area Frac. %	I	S/N
1	5.3	1719.6	100.00	666	148.0

N-(4-Hydroxy-3-methoxybenzyl)-1H-indole-2-carboxamide (5f)

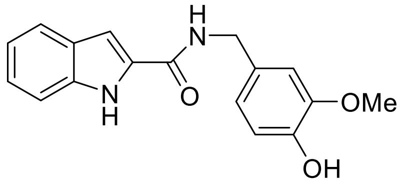

$C_{17}H_{16}N_2O_3$ **MW 296.33**

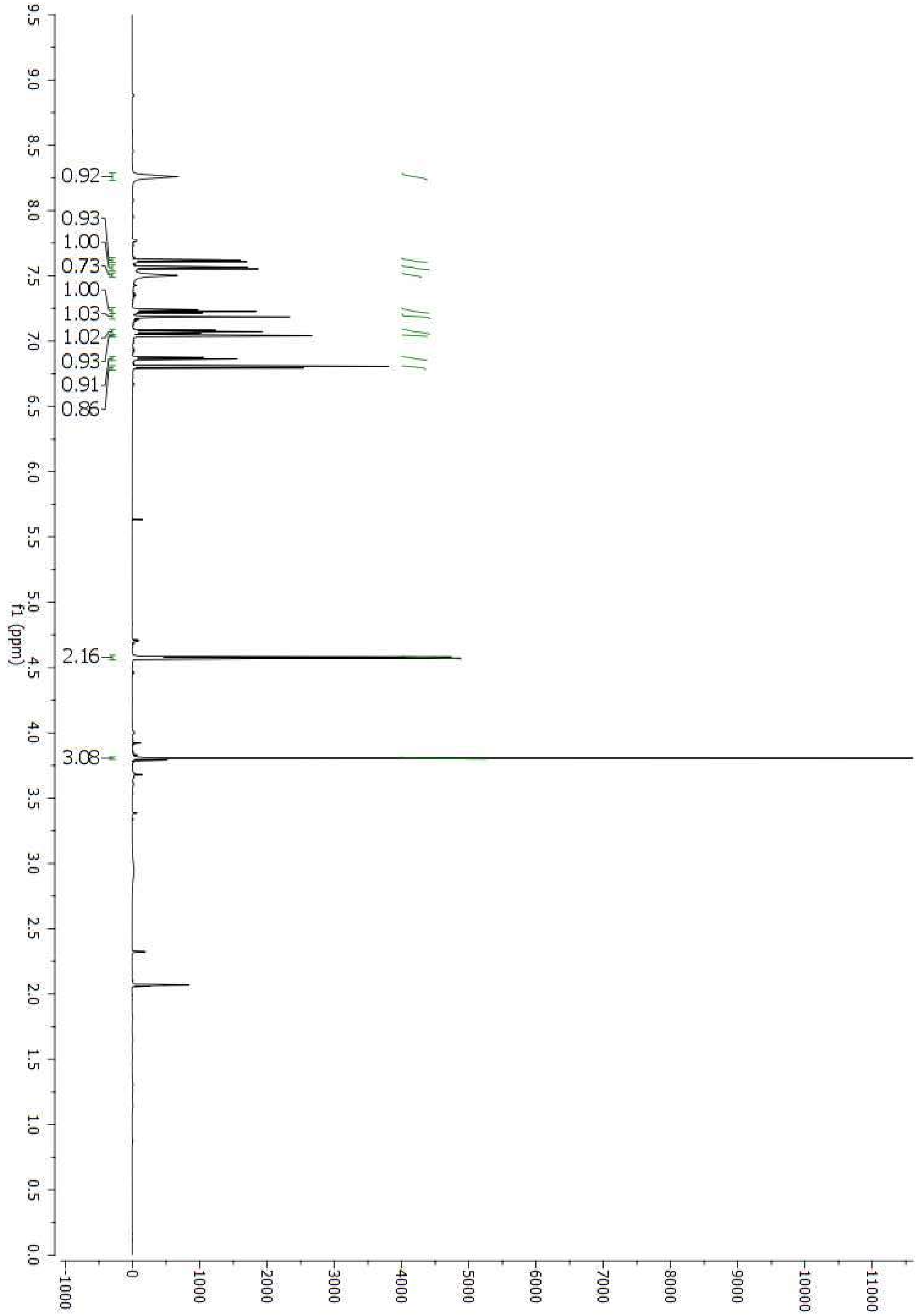

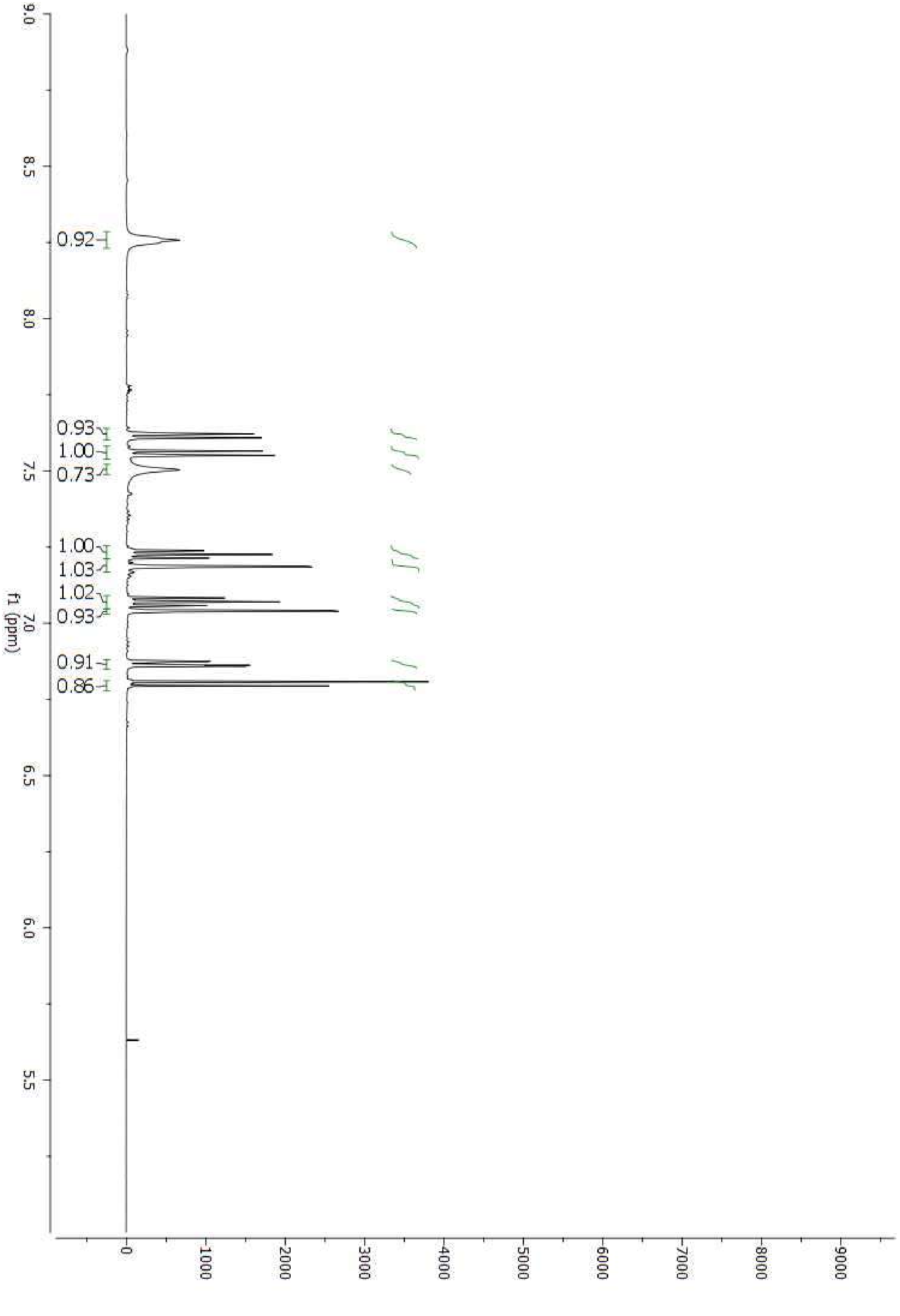

0.92
0.93
1.00
0.73
1.00
1.03
1.02
0.93
0.91
0.86
f1 (ppm)
9.0
8.5
8.0
7.5
7.0
6.5
6.0
5.5
0
1000
2000
3000
4000
5000
6000
7000
8000
9000

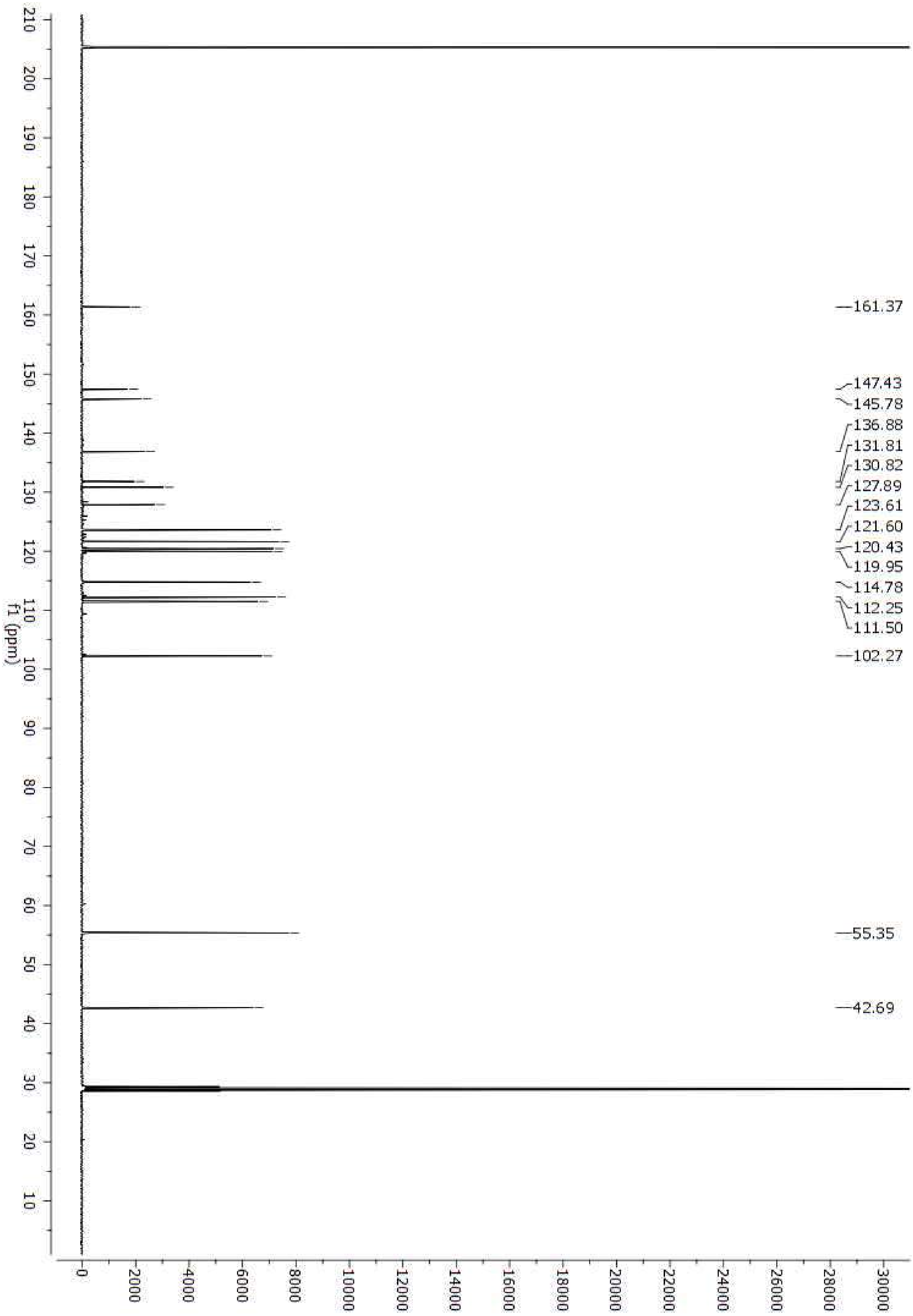

161.37
147.43
145.78
136.88
131.81
130.82
127.89
123.61
121.60
120.43
119.95
114.78
112.25
111.50
102.27
55.35
42.69
f1 (ppm)

Openlynx Report - Page 17
Sample: 8 Vial:1:A,9 File:I-0416572-001
Date:29-Aug-2018 Time:13:30:57

Printed: Thu Aug 30 10:30:57 2018

Sample Report (continued):

Sample 8 Vial 1:A,9 ID File I-0416572-001 Date 29-Aug-2018 Time 13:30:57 Description Met Gen

3: UV Detector: TIC

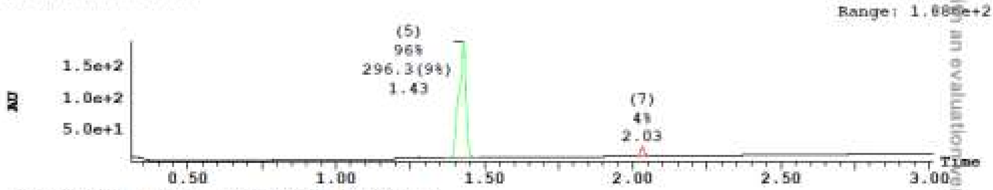

Peak Number	Time	Area %Total	Mass Found
5	1.43	95.78	296.32
7	2.03	4.22	

1: MS ES+ :TIC

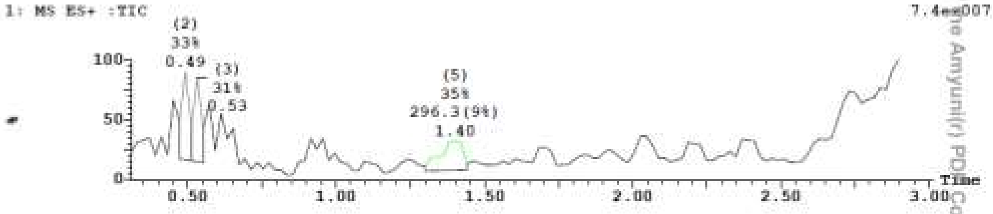

2: MS ES- :TIC

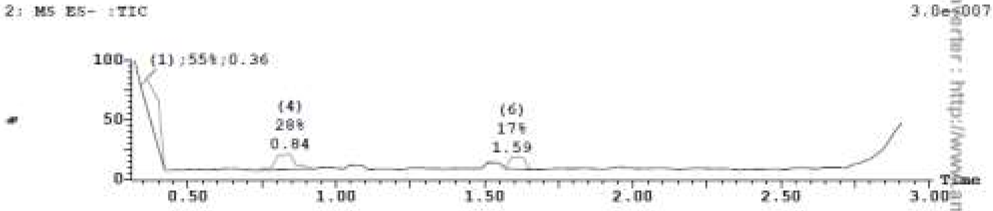

Peak ID	Time	Mass Found
5	1.40	

5: (Time: 1.43) 1:MS ES+ 3.4e+005

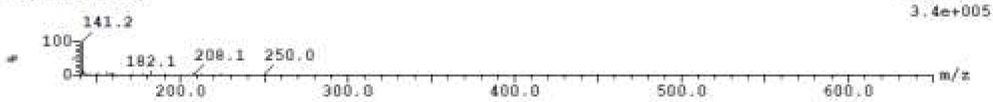

Openlynx Report - Page 18
Sample: 8 Vial:1:A,9 File:I-0416572-001
Date:29-Aug-2018 Time:13:30:57

Printed: Thu Aug 30 10:30:57 2018

Sample Report (continued):

Peak ID	Time	Mass Found
7	2.03	

7: (Time: 2.03) 1:MS ES+ 2.8e+005

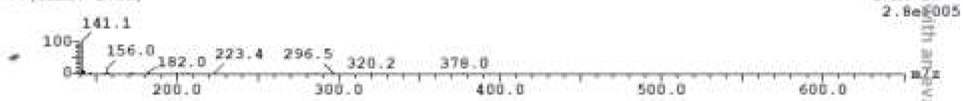

Peak ID	Time	Mass Found
5	1.40	295.32

5: (Time: 1.43) 2:MS ES- 1.4e+003

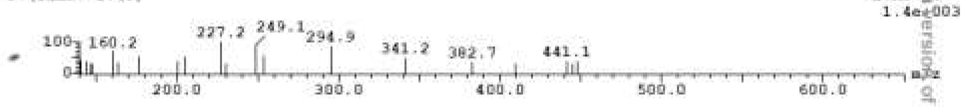

Peak ID	Time	Mass Found
7	2.03	

7: (Time: 2.03) 2:MS ES- 3.1e+003

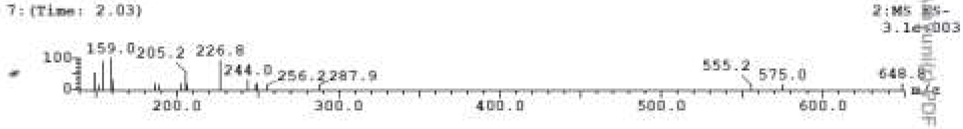

This file was generated with an evaluation version of the Amyuni(r) PDF Converter : http://www.amyuni.com

N-(3,4-Dichlorobenzyl)-1H-indole-2-carboxamide (5g)

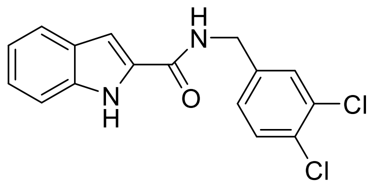

$C_{16}H_{12}Cl_2N_2O$ **MW 319.18**

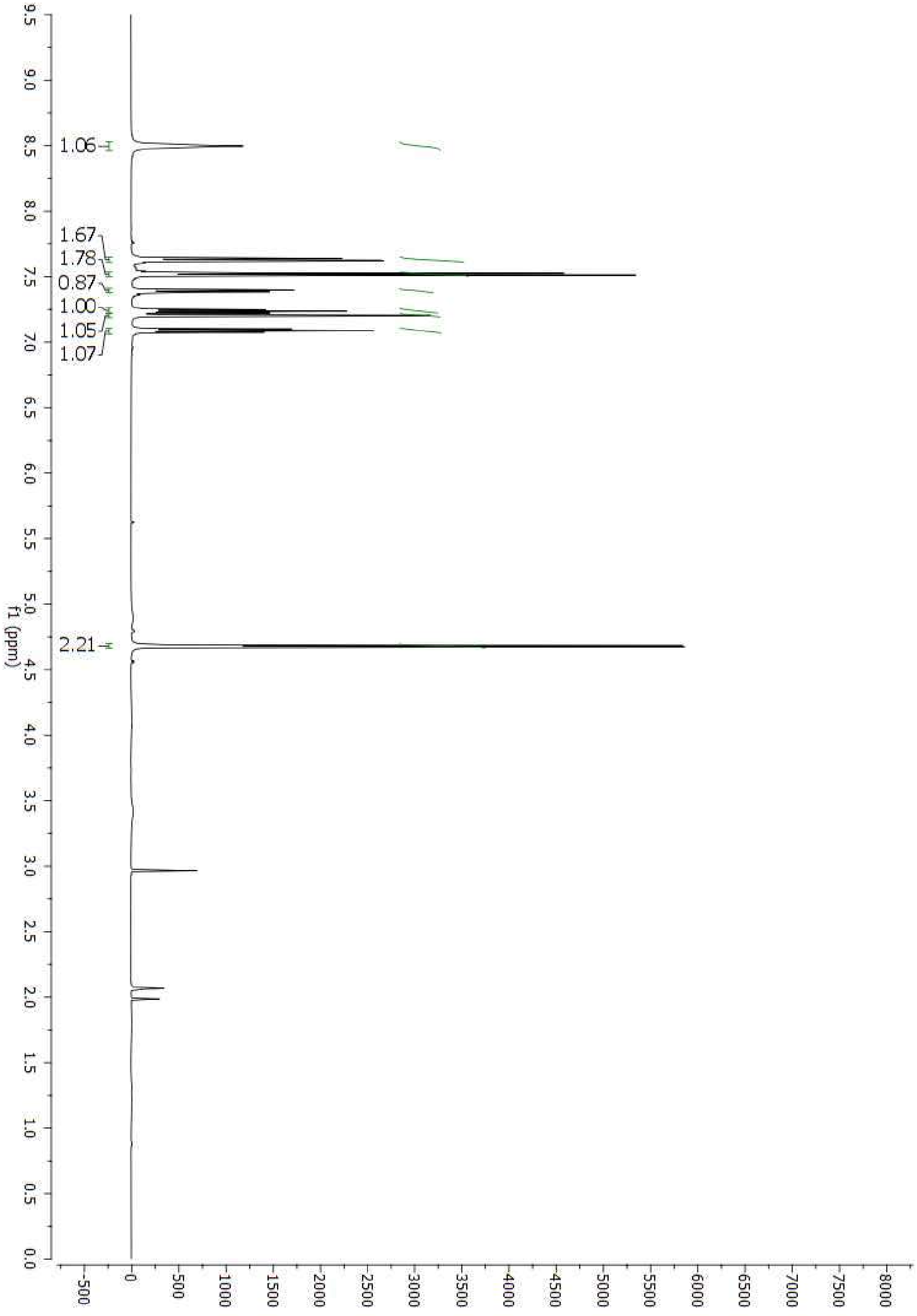

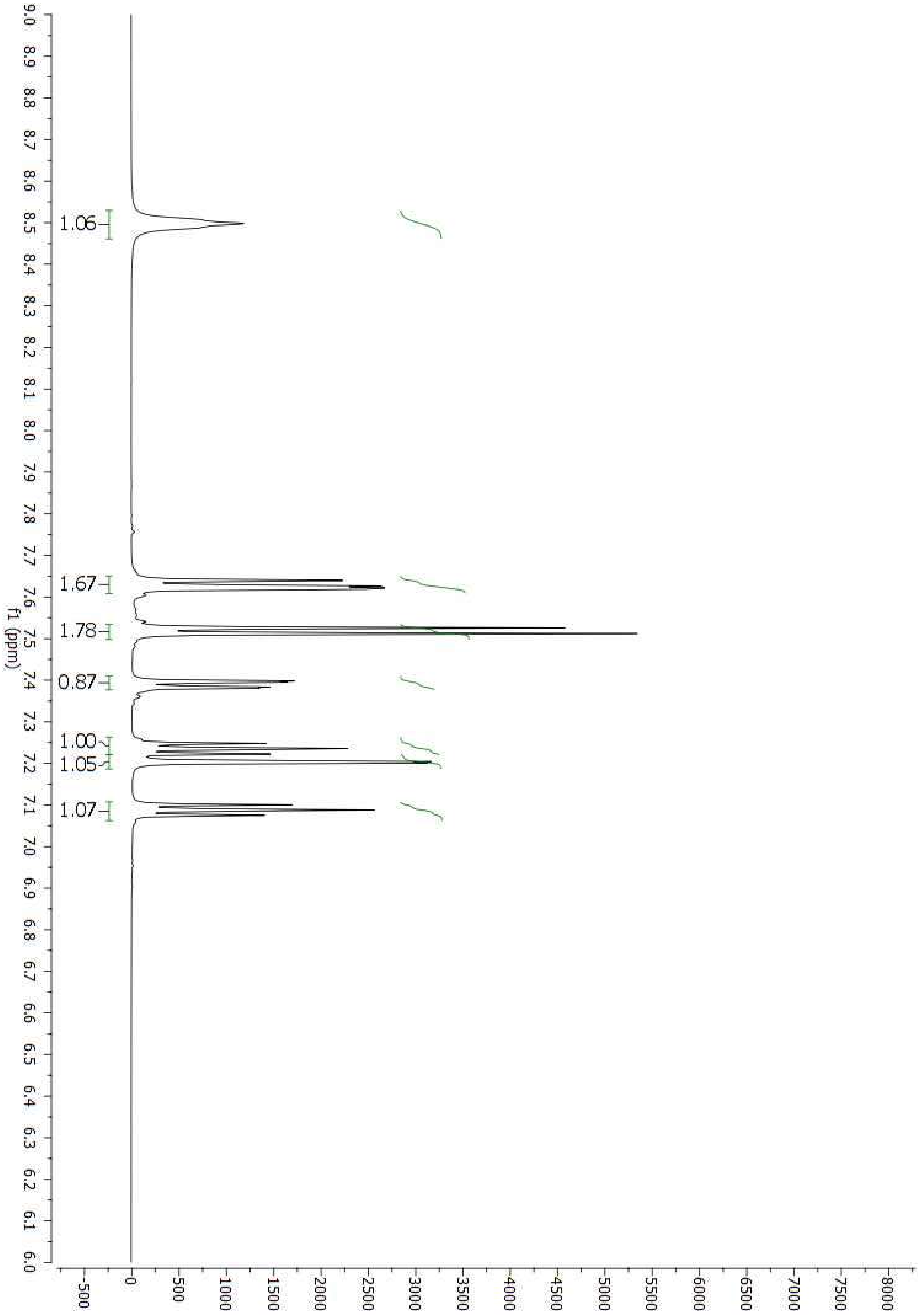
1.06
1.67
1.78
0.87
1.00
1.05
1.07
f1 (ppm)

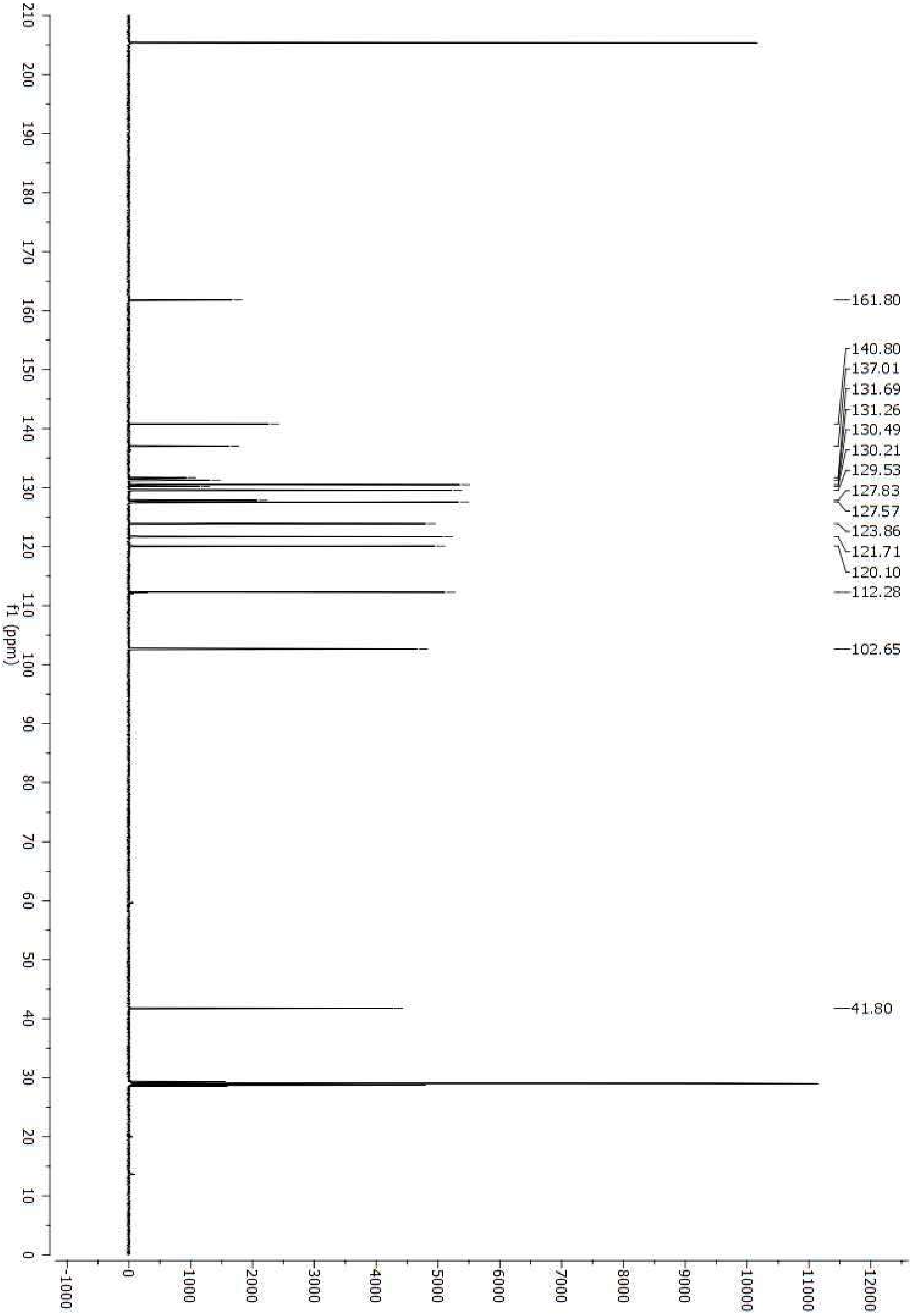
161.80
140.80
137.01
131.69
131.26
130.49
130.21
129.53
127.83
127.57
123.86
121.71
120.10
112.28
102.65
41.80
f1 (ppm)

Analysis Info

Method	MS_LC_POS_TimsOFF.m
Sample Name	MSP26

Acquisition Parameter

Source Type	ESI	Ion Polarity	Positive	Set Nebulizer	2.2 Bar
Focus	Active	Set Capillary	4000 V	Set Dry Heater	220 °C
Scan Begin	50 m/z	Set End Plate Offset	-500 V	Set Dry Gas	10.0 l/min
Scan End	2500 m/z	Set Collision Cell RF	1800.0 Vpp	Set Divert Valve	Waste

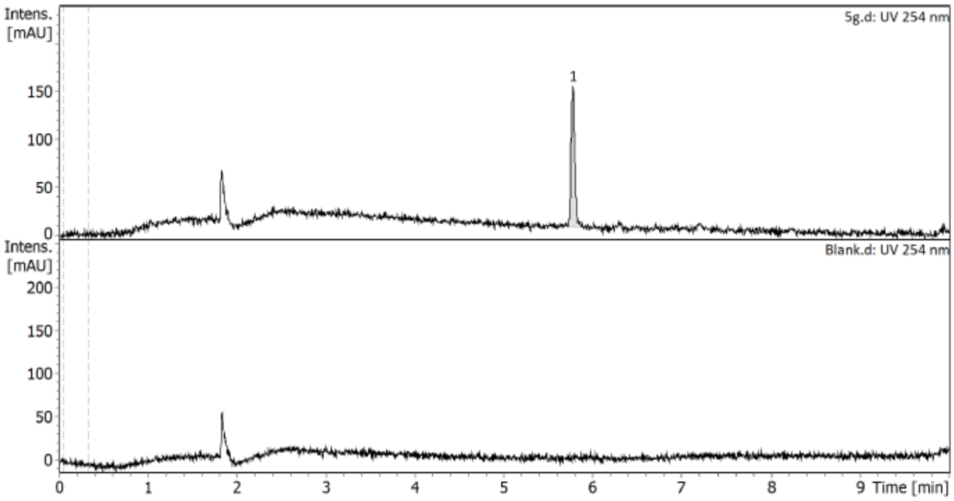

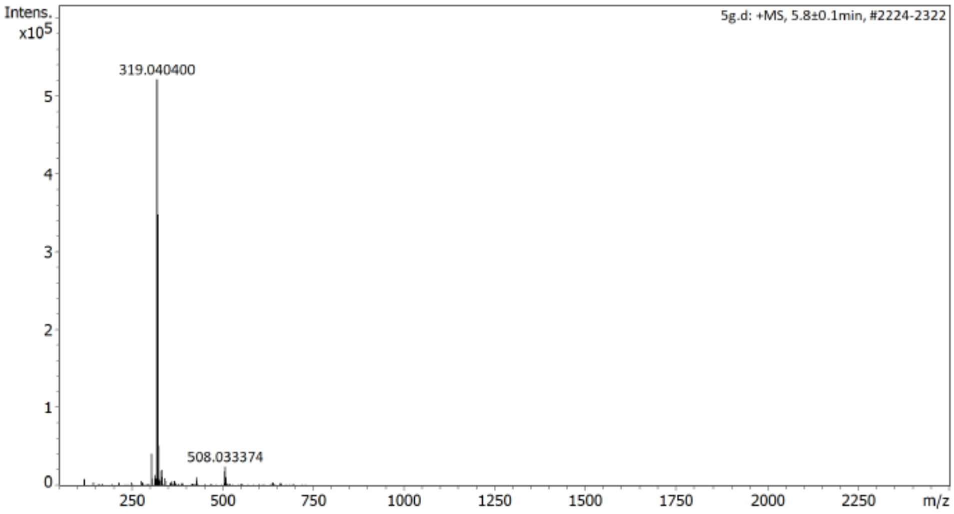

#	RT [min]	Area	Area Frac. %	I	S/N
1	5.8	400.50	100.00	156	63.6

N-(3,4-Difluorobenzyl)-1H-indole-2-carboxamide (5h)

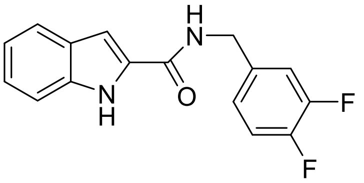

$C_{16}H_{12}F_2N_2O$ **MW 286.28**

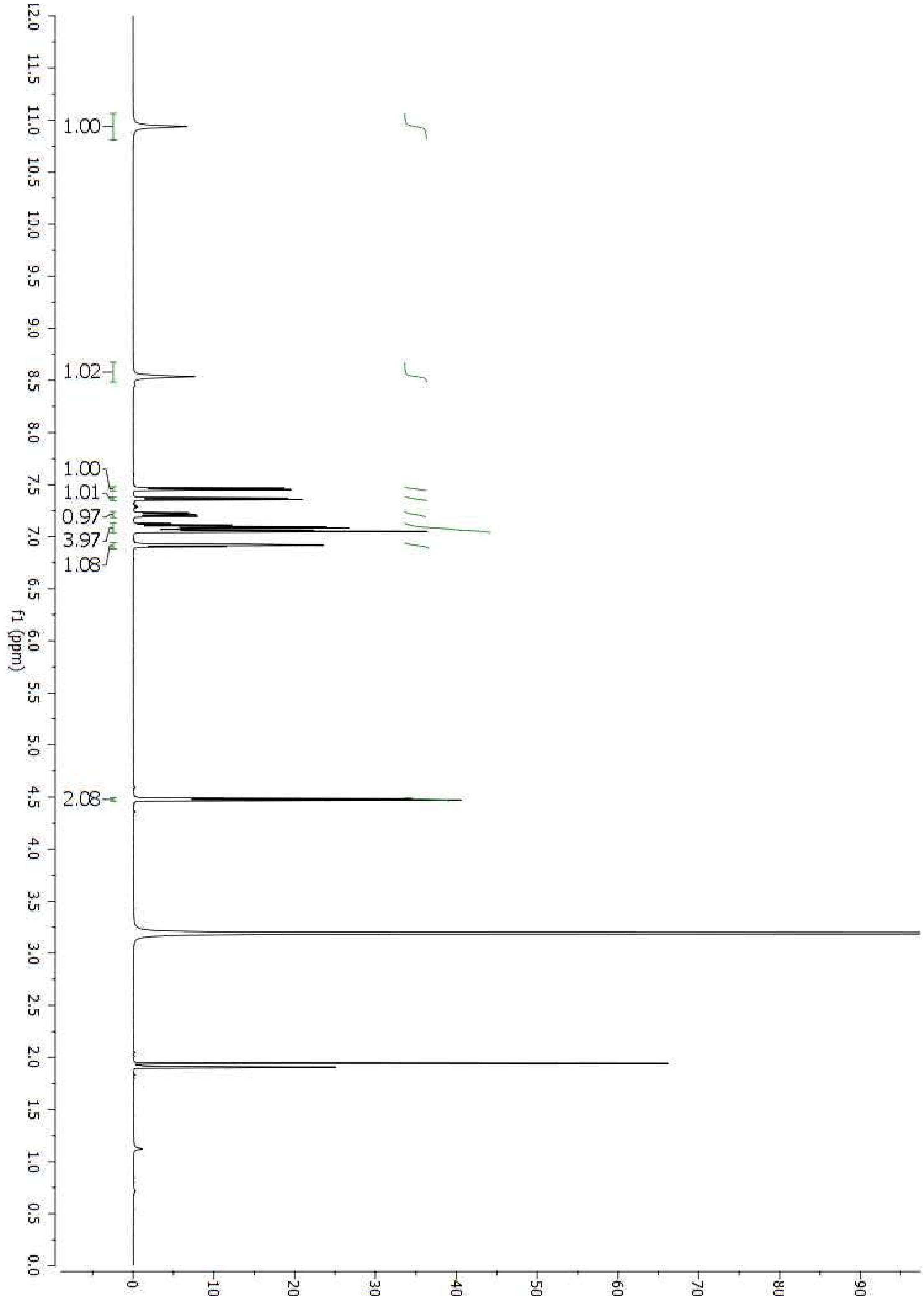

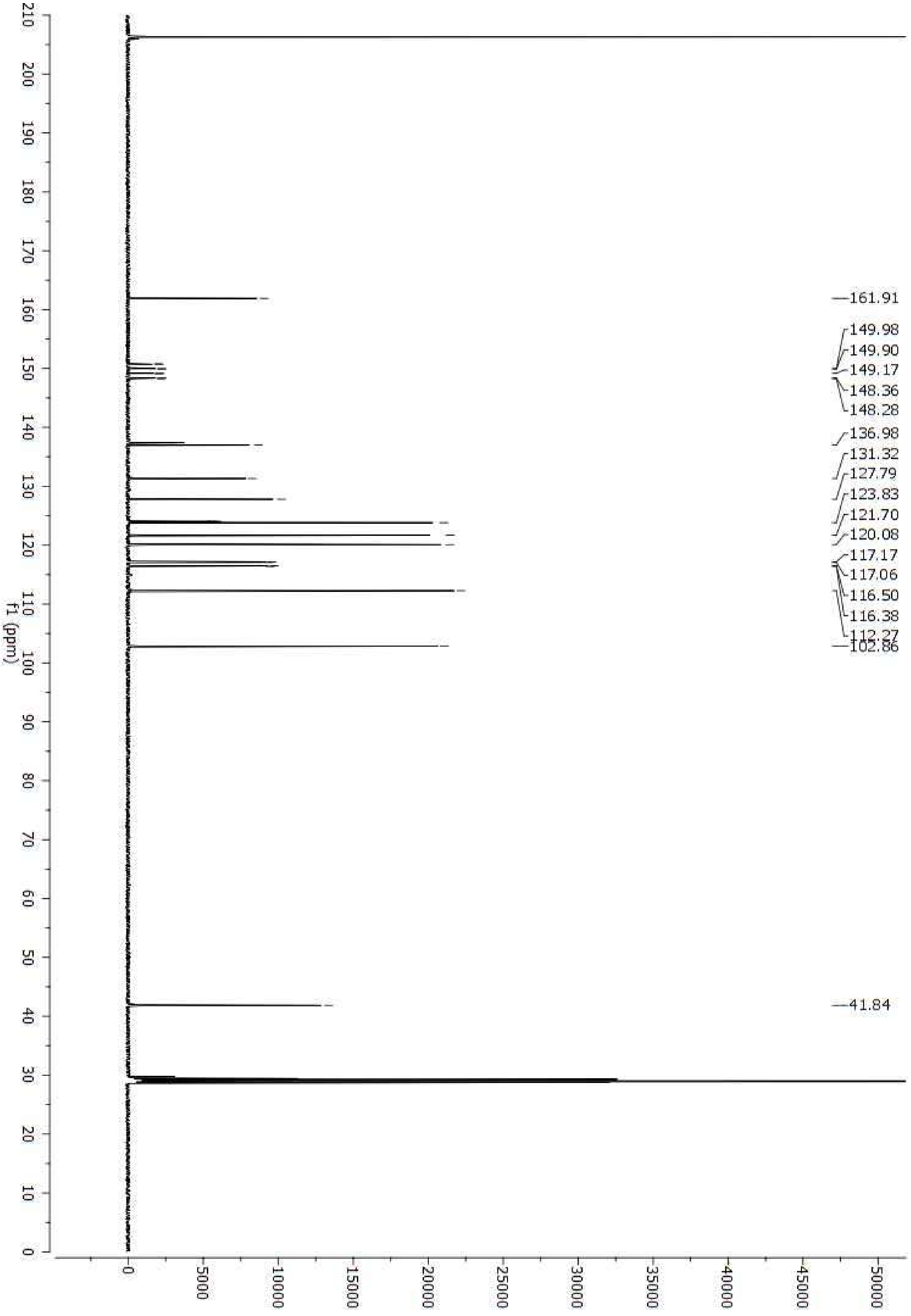
161.91
149.98
149.90
149.17
148.36
148.28
136.98
131.32
127.79
123.83
121.70
120.08
117.17
117.06
116.50
116.38
112.27
102.86
41.84
f1 (ppm)

Analysis Info

Method	MS_LC_POS_TimsOFF.m
Sample Name	MSP138

Acquisition Parameter

Source Type	ESI	Ion Polarity	Positive	Set Nebulizer	2.2 Bar
Focus	Active	Set Capillary	4000 V	Set Dry Heater	220 °C
Scan Begin	50 m/z	Set End Plate Offset	-500 V	Set Dry Gas	10.0 l/min
Scan End	2500 m/z	Set Collision Cell RF	1800.0 Vpp	Set Divert Valve	Waste

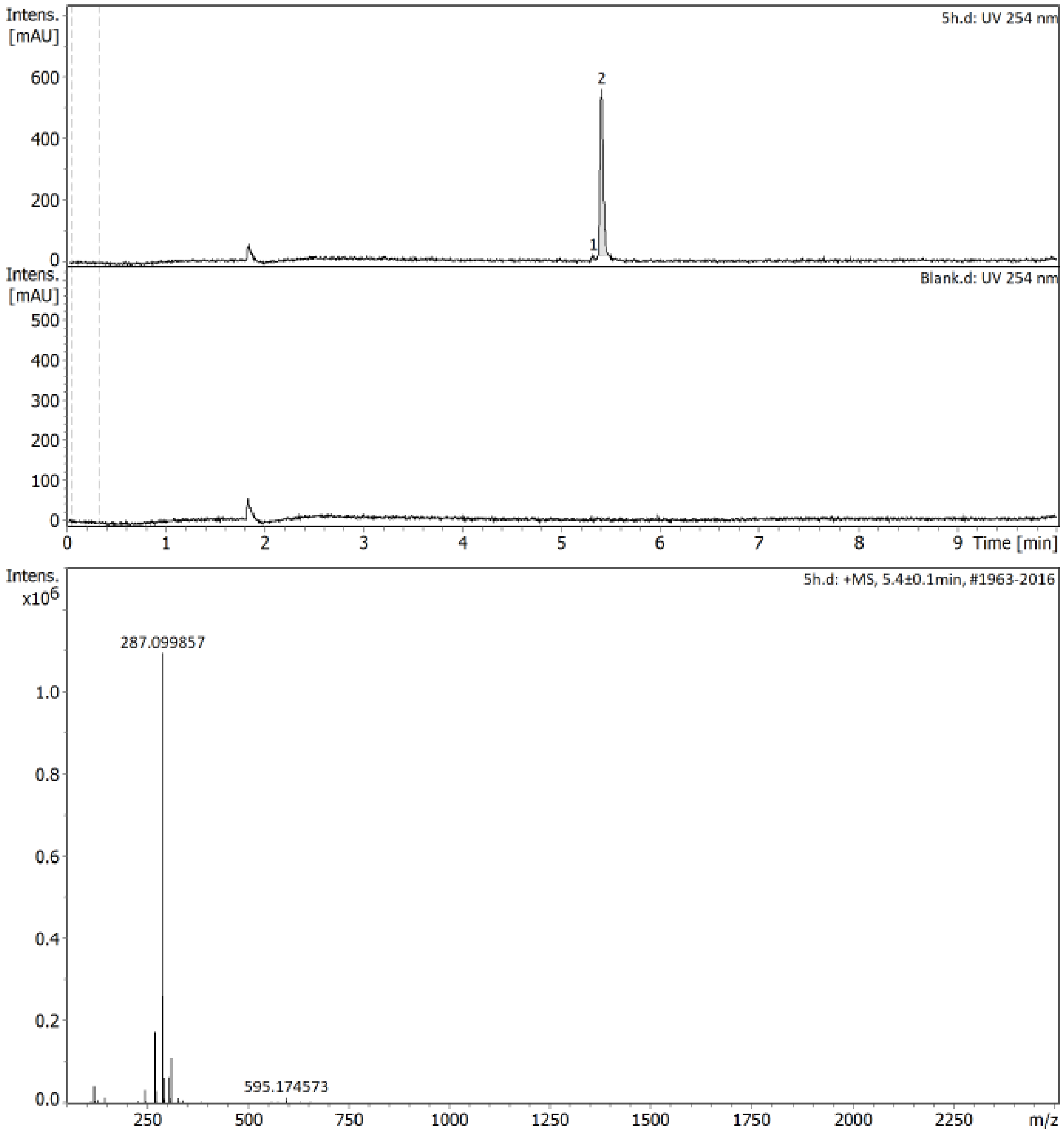

#	RT [min]	Area	Area Frac. %	I	S/N
1	5.3	24.080	1.79	23	5.3
2	5.4	1322.066	98.21	563	180.8

N-(3,4-Dihydroxyphenethyl)-1H-indole-2-carboxamide (5i)

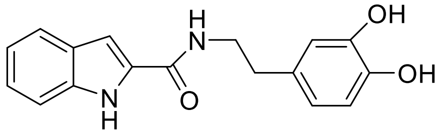

$C_{17}H_{16}N_2O_3$ **MW 296.33**

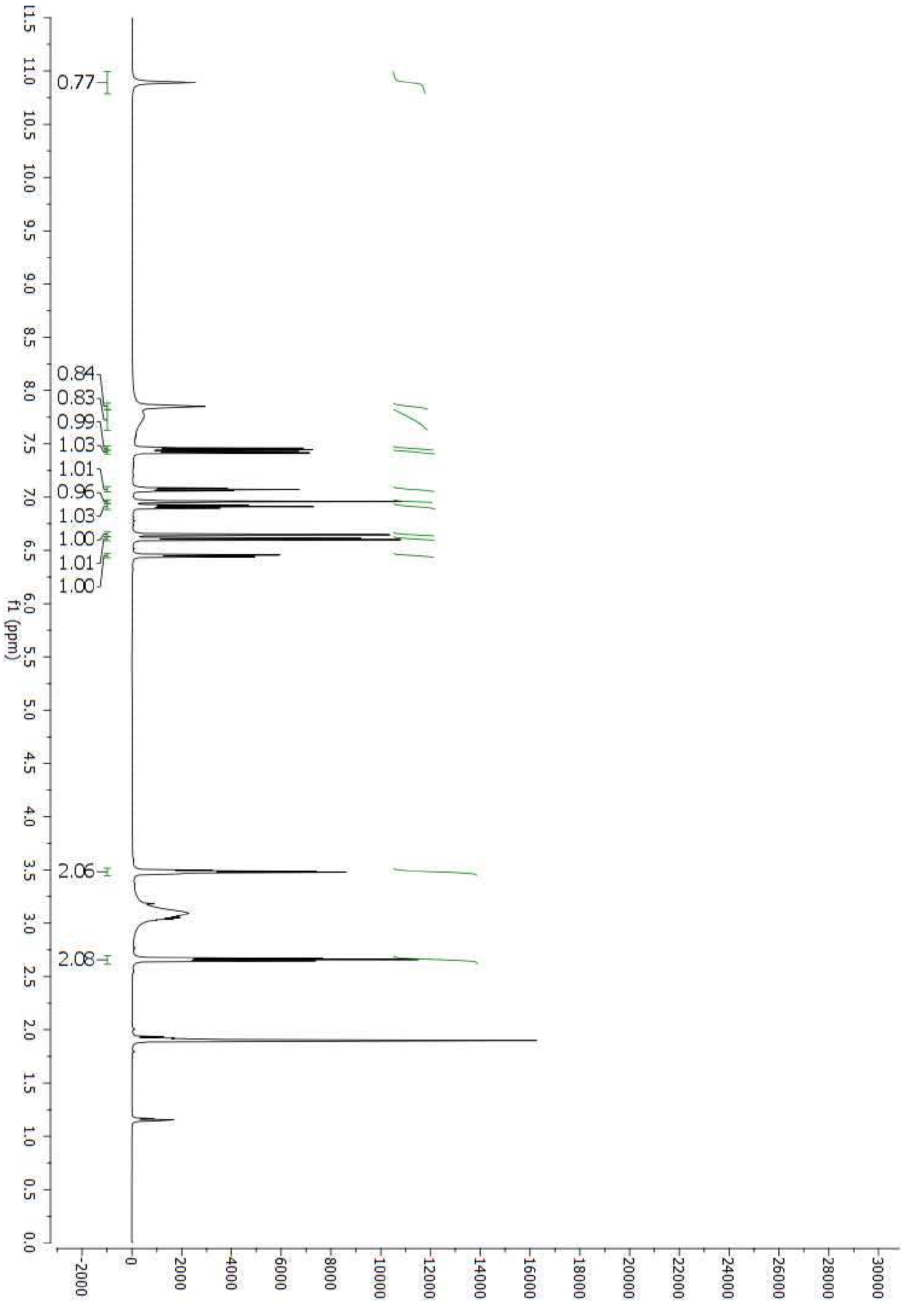

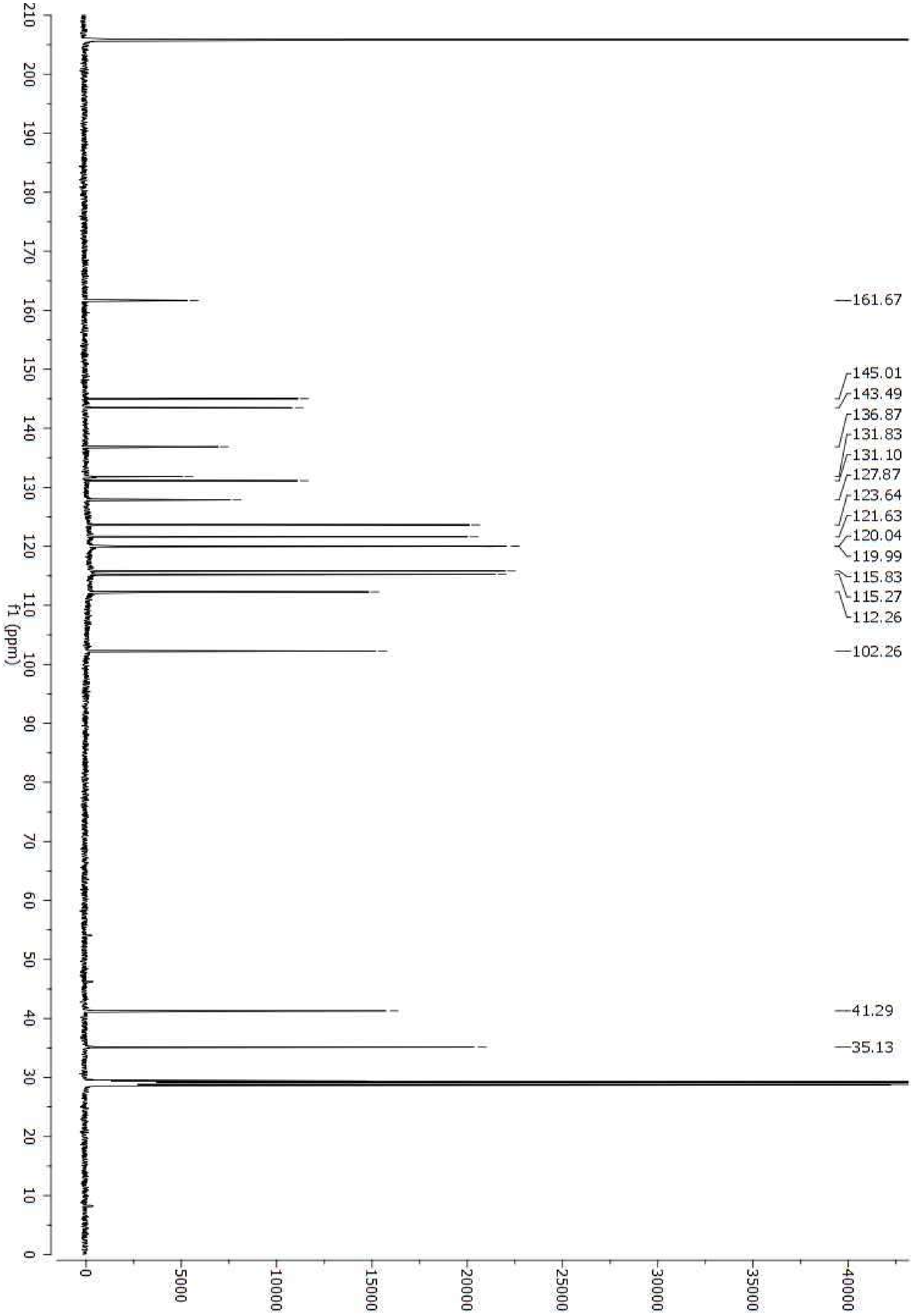
161.67
145.01
143.49
136.87
131.83
131.10
127.87
123.64
121.63
120.04
119.99
115.83
115.27
112.26
102.26
41.29
35.13
f1 (ppm)

Analysis Info

Method	MS_LC_POS_TimsOFF.m
Sample Name	MSP28

Acquisition Parameter

Source Type	ESI	Ion Polarity	Positive	Set Nebulizer	2.2 Bar
Focus	Active	Set Capillary	4000 V	Set Dry Heater	220 °C
Scan Begin	50 m/z	Set End Plate Offset	-500 V	Set Dry Gas	10.0 l/min
Scan End	2500 m/z	Set Collision Cell RF	1800.0 Vpp	Set Divert Valve	Waste

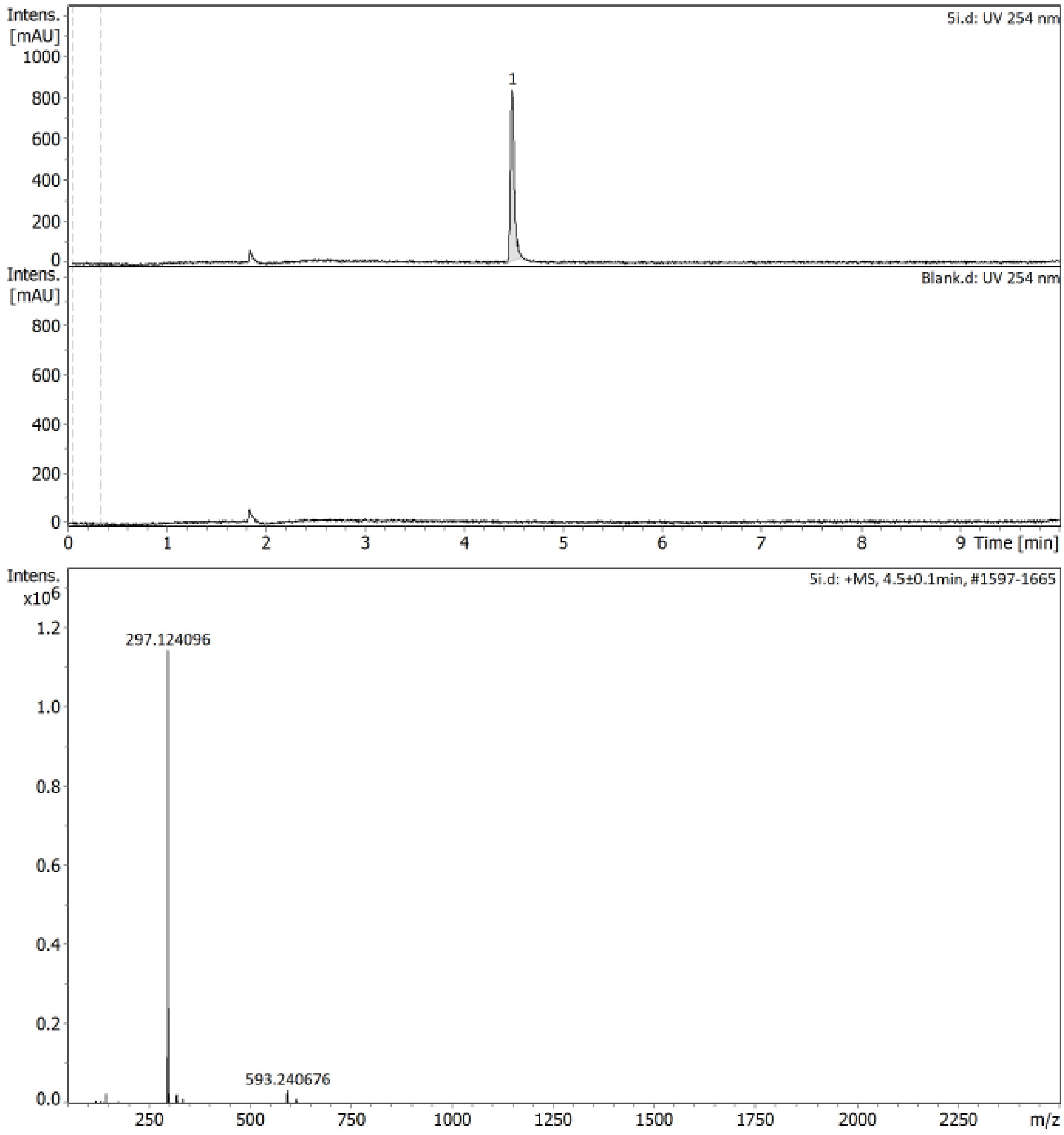

#	RT [min]	Area	Area Frac. %	I	S/N
1	4.5	2220.1	100.00	840	277.2

N-(4-Hydroxyphenyl)-1-methyl-1*H*-indole-2-carboxamide (6a)

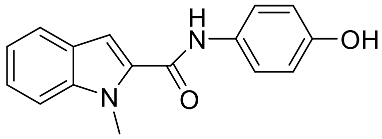

$C_{16}H_{14}N_2O_2$ **MW 266.30**

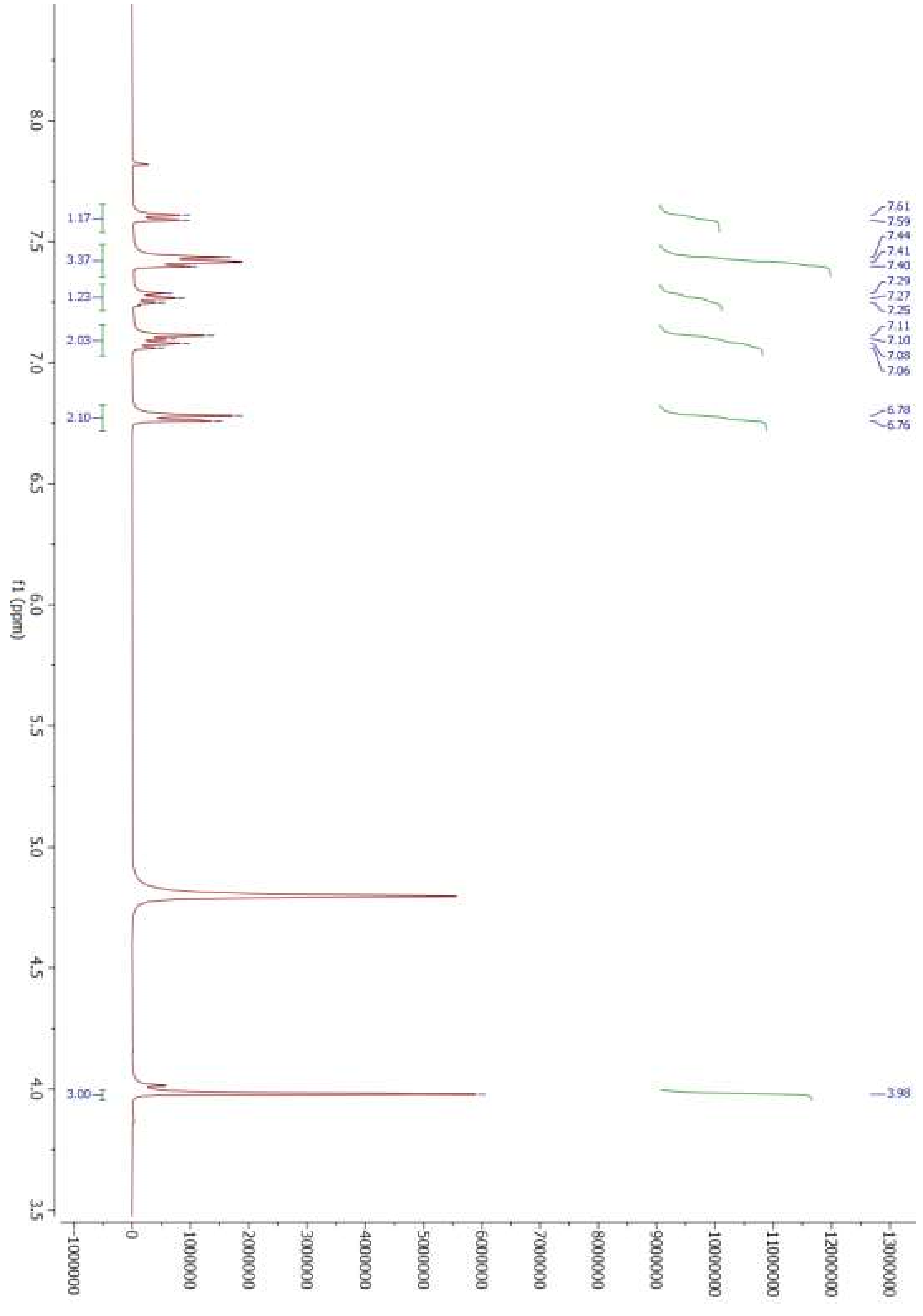

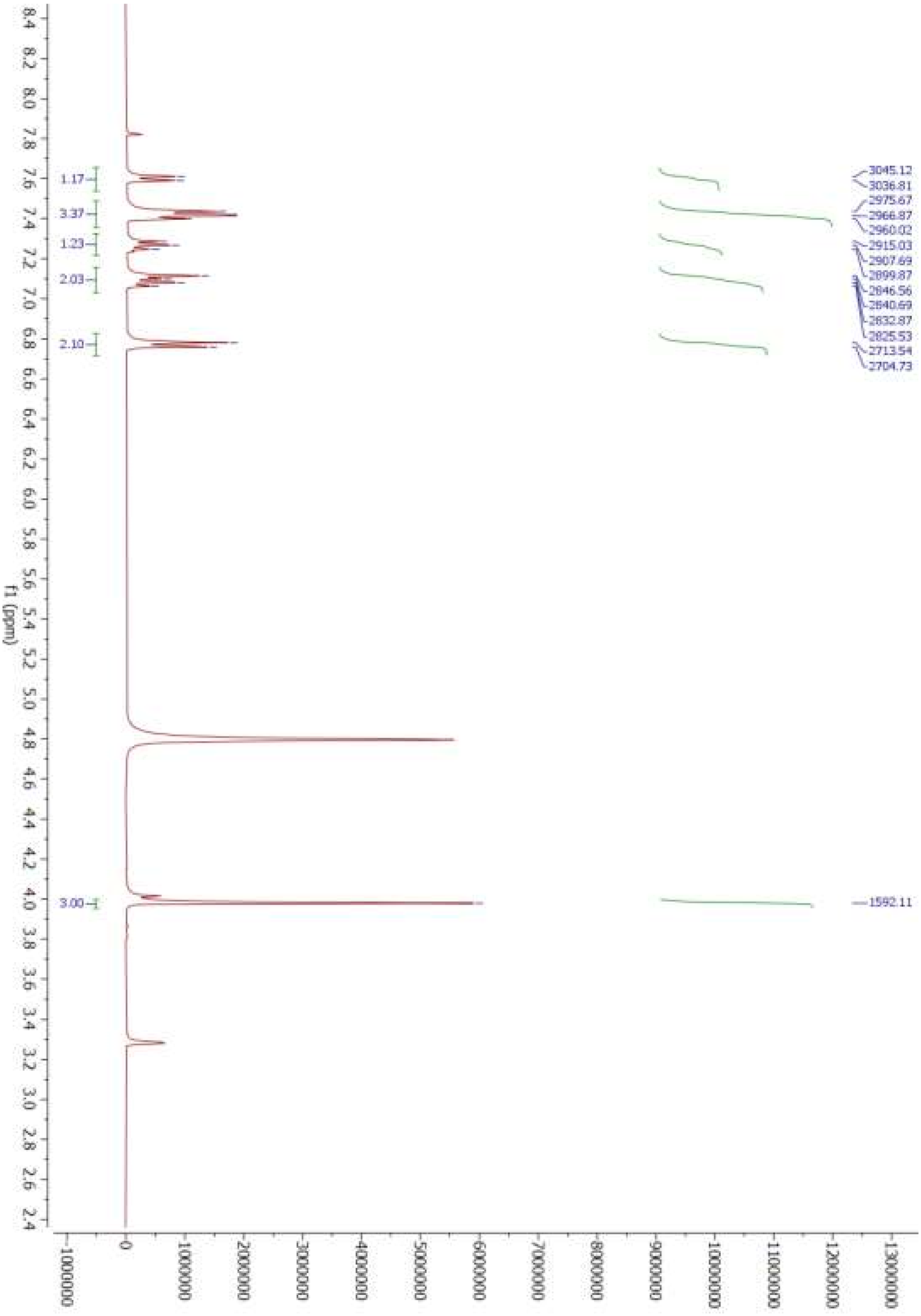
3045.12
3036.81
2975.67
2966.87
2960.02
2915.03
2907.69
2899.87
2846.56
2840.69
2832.87
2825.53
2713.54
2704.73
1592.11
1.17
3.37
1.23
2.03
2.10
3.00
f1 (ppm)

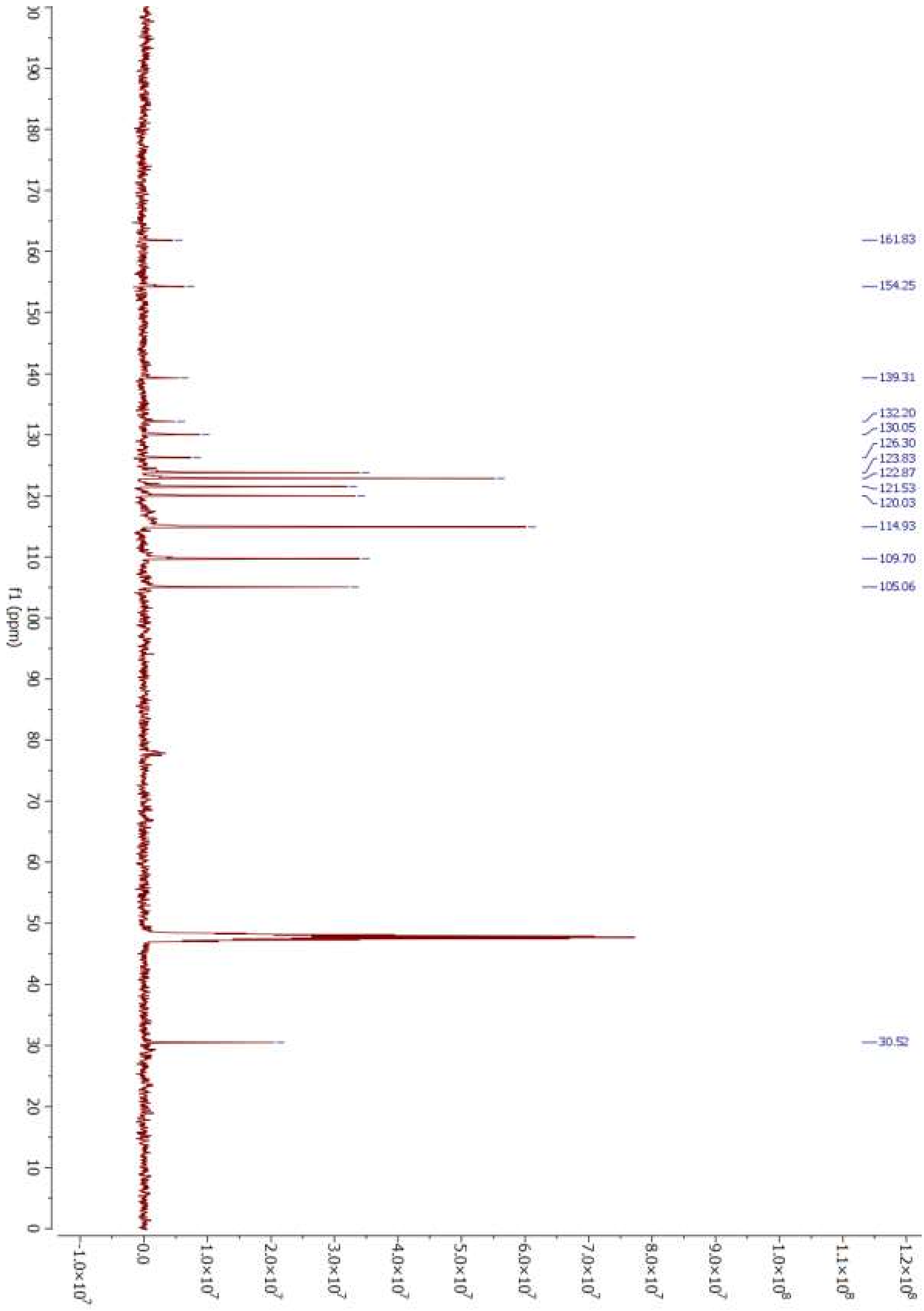
161.83
154.25
139.31
132.20
130.05
126.30
123.83
122.87
121.53
120.03
114.93
109.70
105.06
30.52
f1 (ppm)

Sample Report (continued):

Sample 28 Vial 1:C,9 ID File I-0416592-001 Date 29-Aug-2018 Time 14:59:16 Description Met Gen

3: UV Detector: TIC 2.86e+2
Range: 2.86e+2

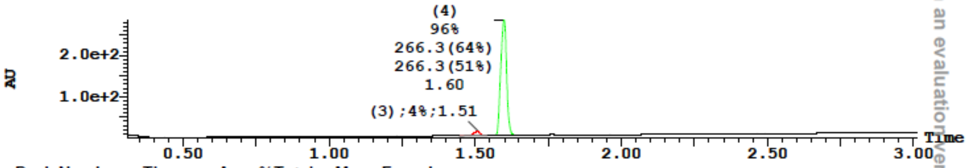

Peak Number	Time	Area %Total	Mass Found
3	1.51	3.66	
4	1.60	96.34	266.29, 266.29

1: MS ES+ :TIC 3.3e+009

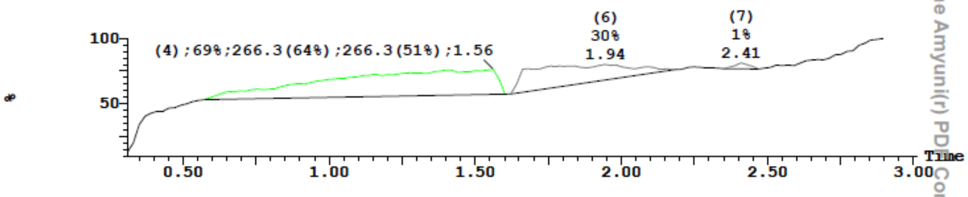

2: MS ES- :TIC 9.7e+007

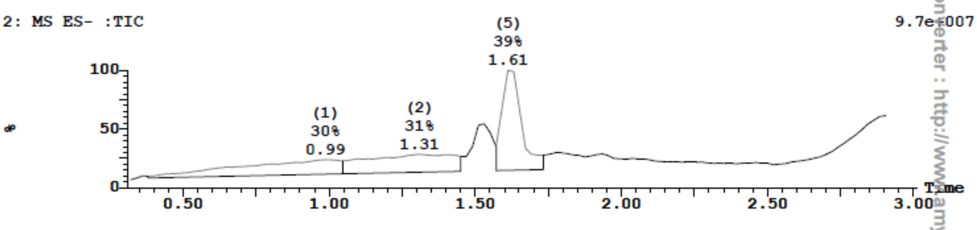

Peak ID	Time	Mass Found
3	1.51	

3: (Time: 1.51) 1:MS ES+
2.9e+006

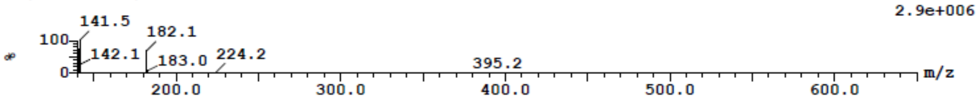

Sample Report (continued):

Peak ID	Time	Mass Found
4	1.56	267.29

4: (Time: 1.60) 1:MS ES+
6.1e+006

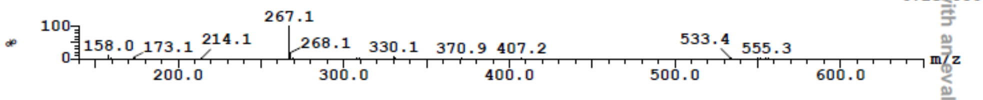

Peak ID	Time	Mass Found
3	1.51	

3: (Time: 1.51) 2:MS ES-
2.8e+005

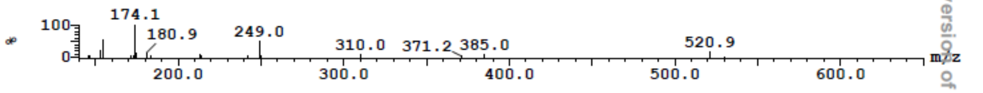

Peak ID	Time	Mass Found
4	1.56	265.29

4: (Time: 1.60) 2:MS ES-
1.2e+006

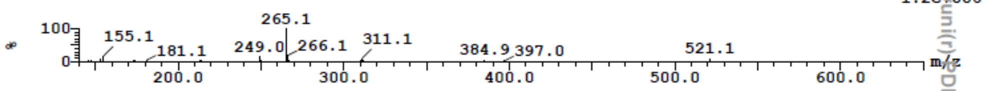

This file was generated with an evaluation version of the Amyuni(r) PDF Converter : http://www.amyuni.com

N-(4-Fluorophenyl)-1-methyl-1H-indole-2-carboxamide (6b)

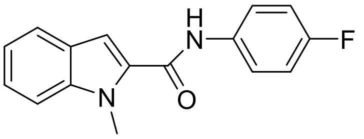

$C_{16}H_{13}FN_2O$ **MW 268.29**

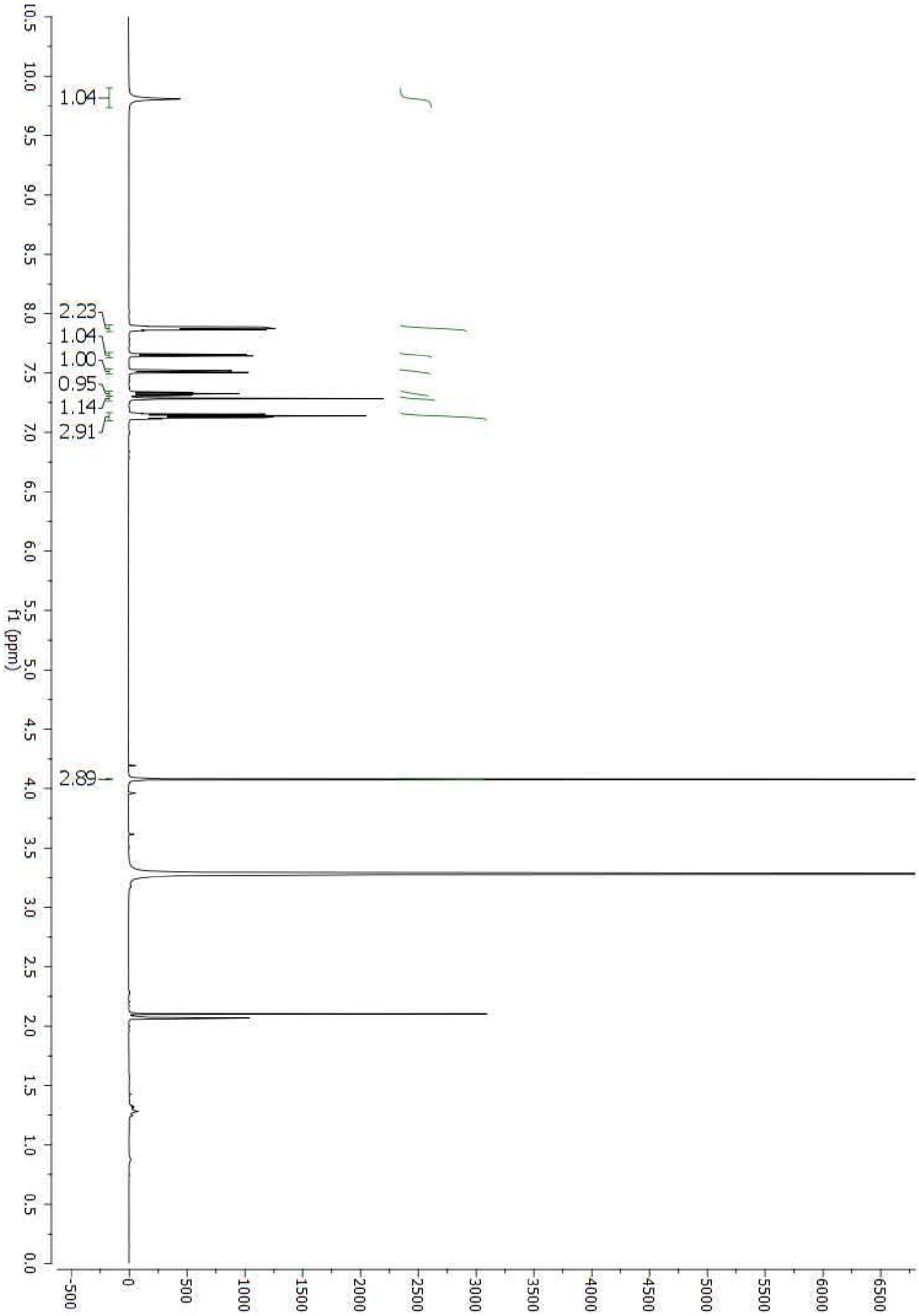

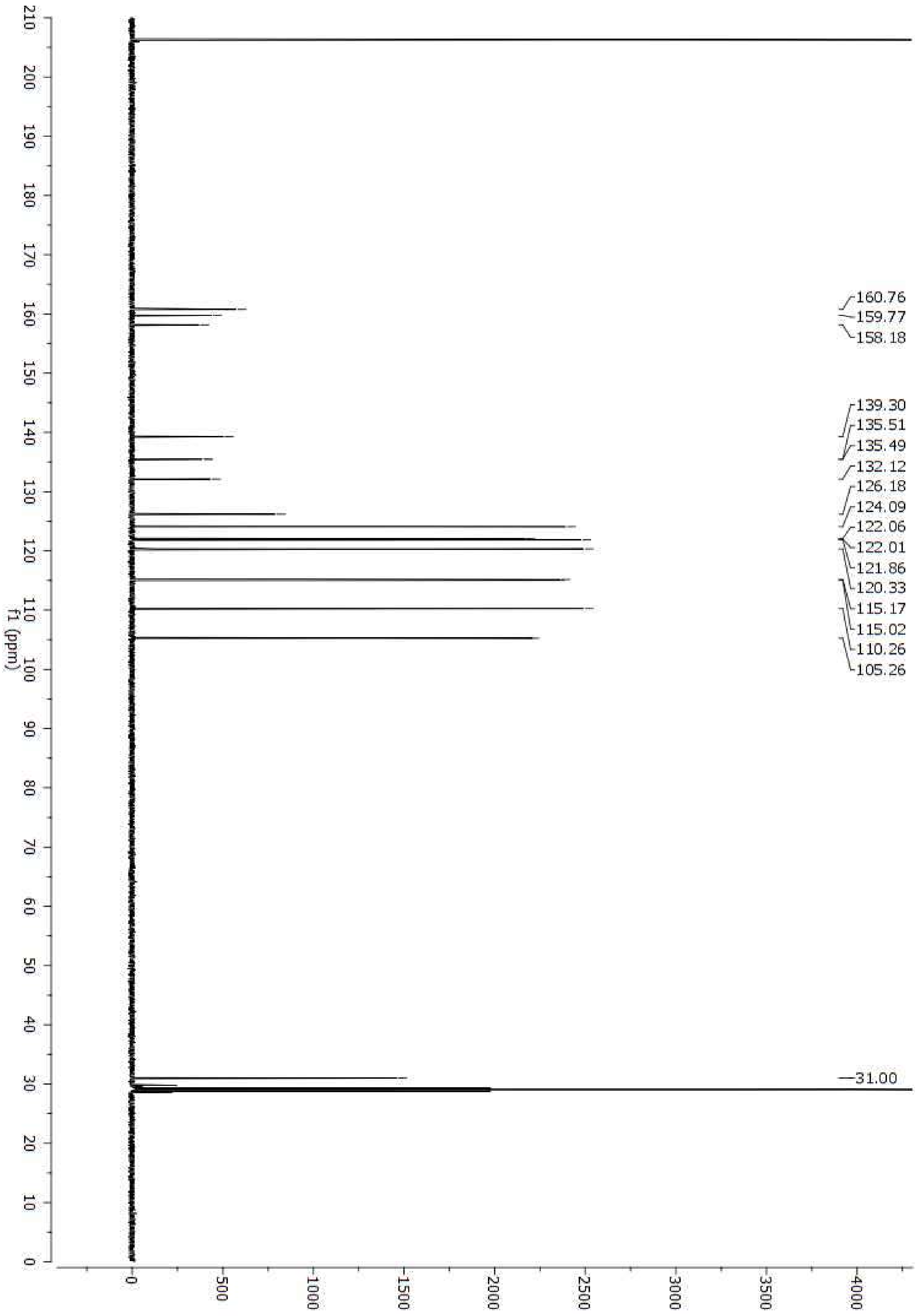
160.76
159.77
158.18
139.30
135.51
135.49
132.12
126.18
124.09
122.06
122.01
121.86
120.33
115.17
115.02
110.26
105.26
31.00
f1 (ppm)

Analysis Info

Method	MS_LC_POS_TimsOFF.m
Sample Name	MSP141

Acquisition Parameter

Source Type	ESI	Ion Polarity	Positive	Set Nebulizer	2.2 Bar
Focus	Active	Set Capillary	4000 V	Set Dry Heater	220 °C
Scan Begin	50 m/z	Set End Plate Offset	-500 V	Set Dry Gas	10.0 l/min
Scan End	2500 m/z	Set Collision Cell RF	1800.0 Vpp	Set Divert Valve	Waste

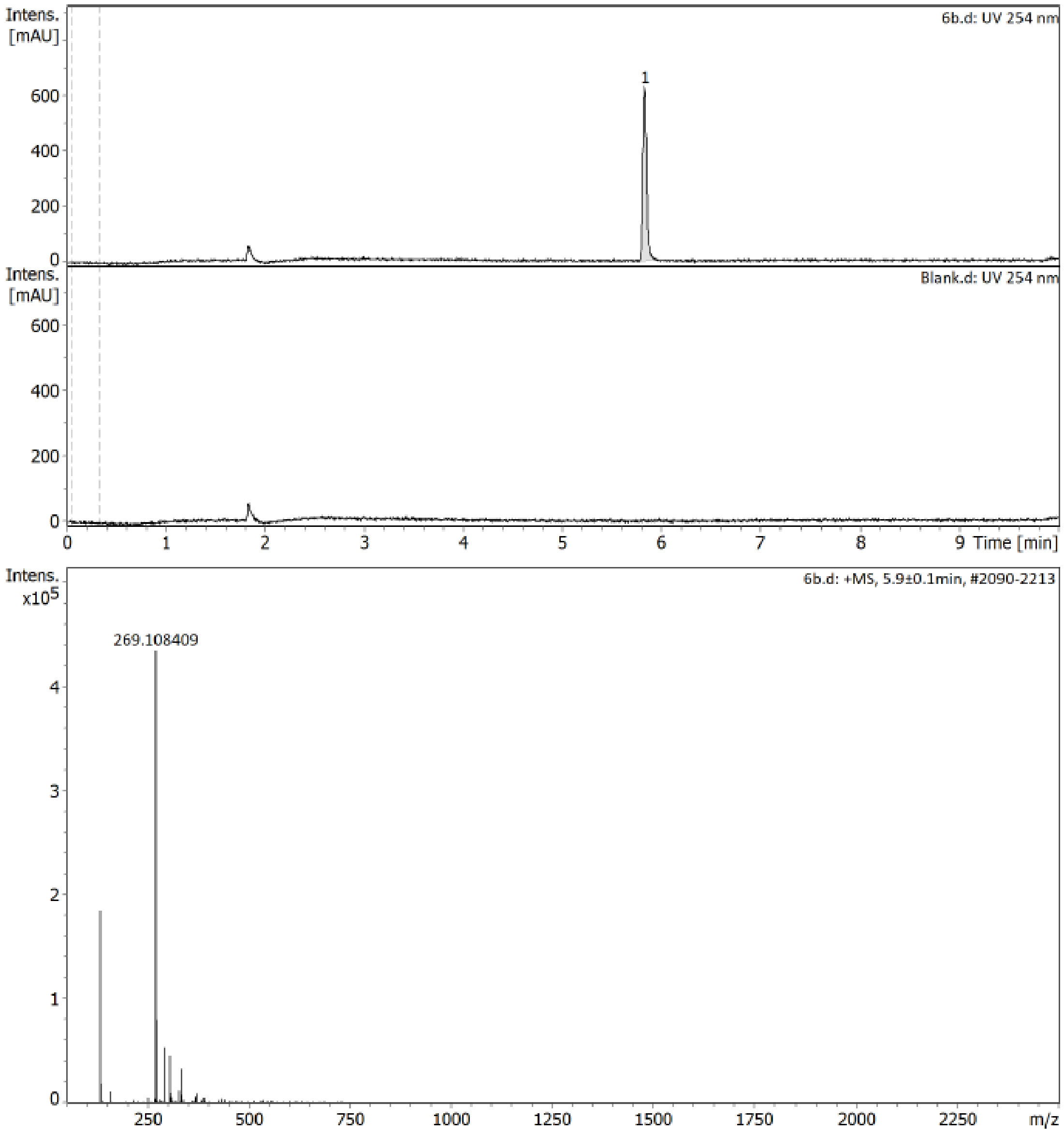

#	RT [min]	Area	Area Frac. %	I	S/N
1	5.8	1734.0	100.00	626	264.9

N-Benzyl-1-methyl-1H-indole-2-carboxamide (6c)

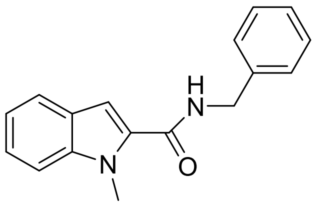

$C_{17}H_{16}N_2O$ **MW 264.33**

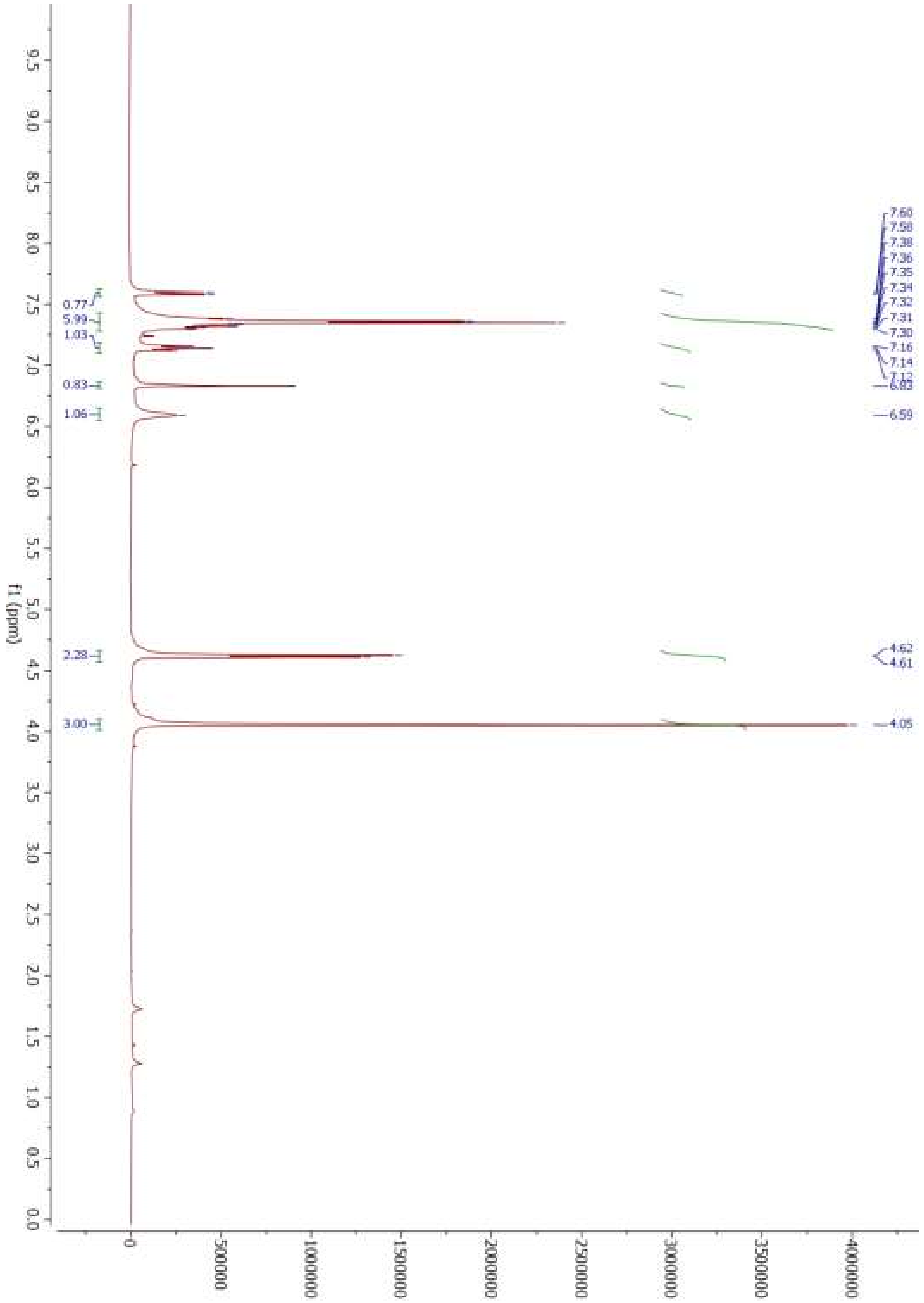

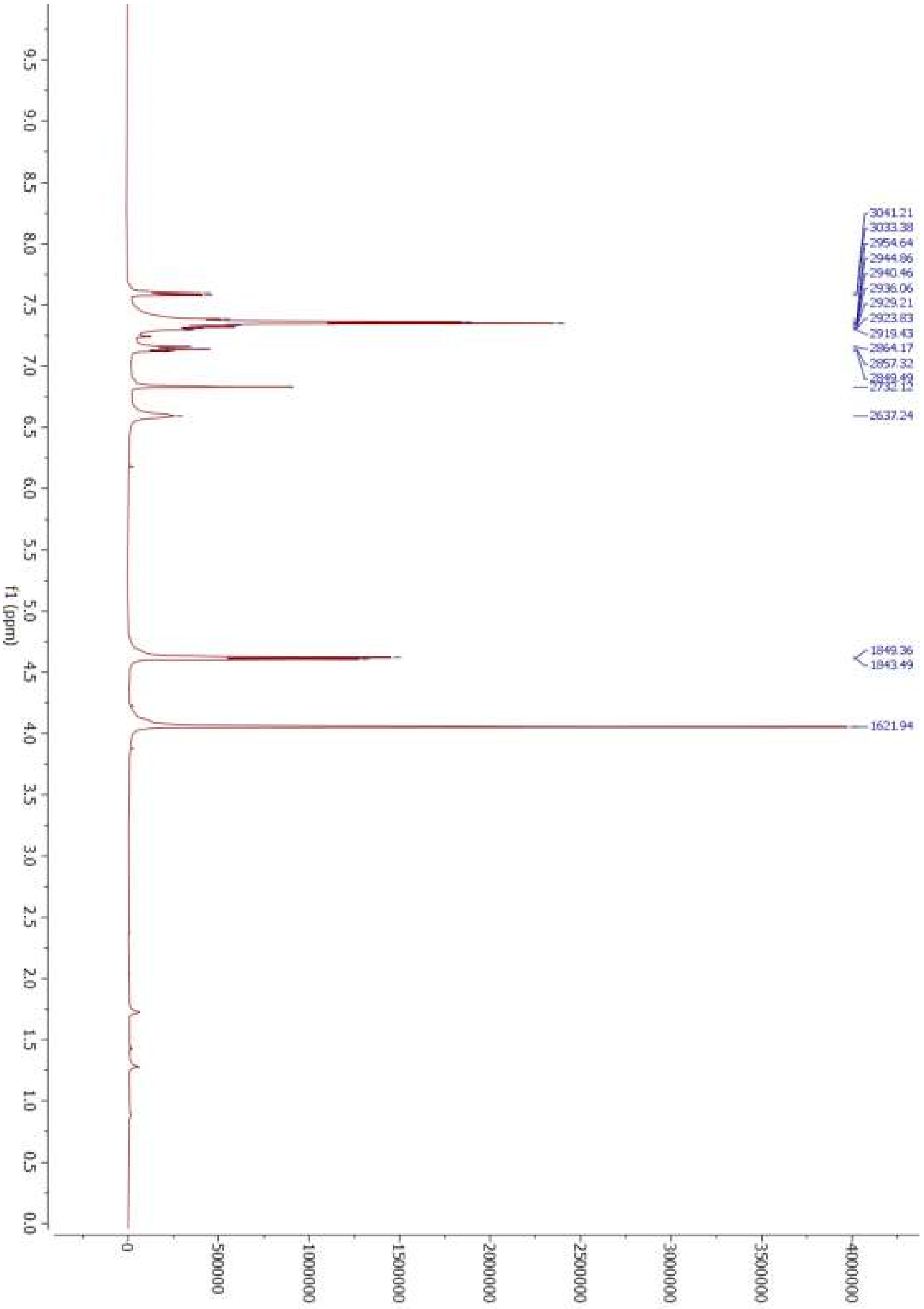
3041.21
3033.38
2954.64
2944.86
2940.46
2936.06
2929.21
2923.83
2919.43
2864.17
2857.32
2732.12
2637.24
1849.36
1843.49
1621.94
f1 (ppm)

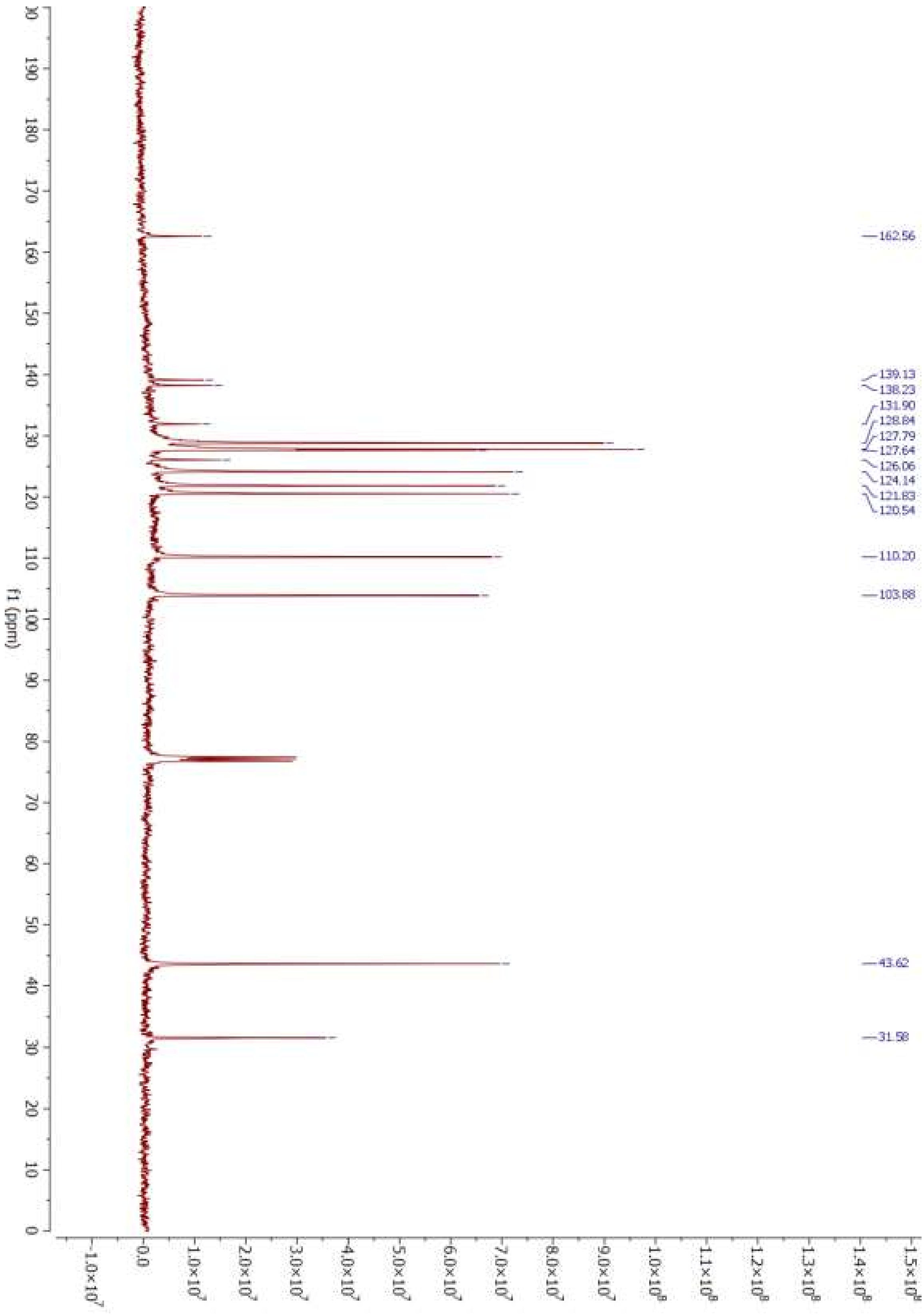
162.56
139.13
138.23
131.90
128.84
127.79
127.64
126.06
124.14
121.83
120.54
110.20
103.88
43.62
31.58
f1 (ppm)

Sample: 65 Vial:1:G,6 File:I-0416627-001
Date:29-Aug-2018 Time:17:43:20

Printed: Thu Aug 30 10:30:57 2018

Sample Report (continued):

Sample 65 Vial 1:G,6 ID File I-0416627-001 Date 29-Aug-2018 Time 17:43:20 Description Met Gen

3: UV Detector: TIC 2.332e+2
Range: 2.328e+2

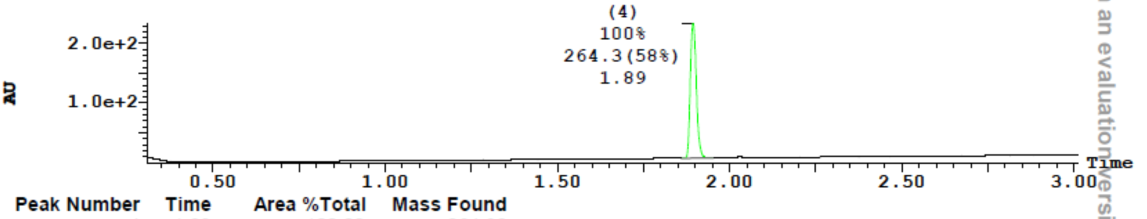

Peak Number	Time	Area %Total	Mass Found
4	1.89	100.00	264.32

1: MS ES+ :TIC 1.5e+009

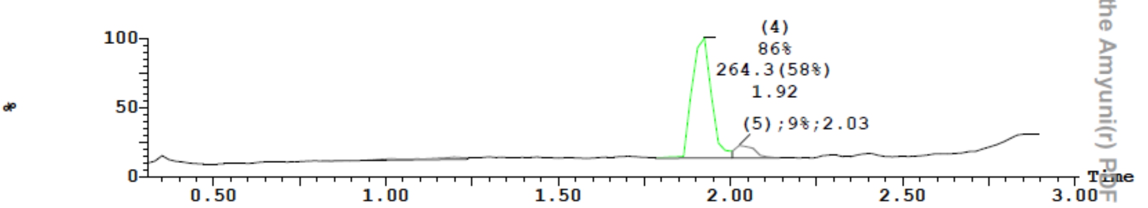

2: MS ES- :TIC 6.2e+007

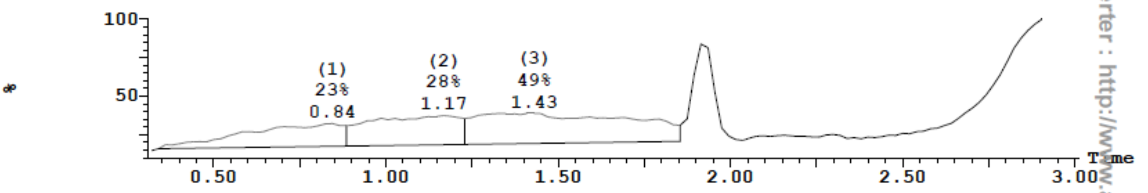

Peak ID	Time	Mass Found
4	1.92	265.32

4:(Time: 1.89) 1:MS ES+
2.3e+007

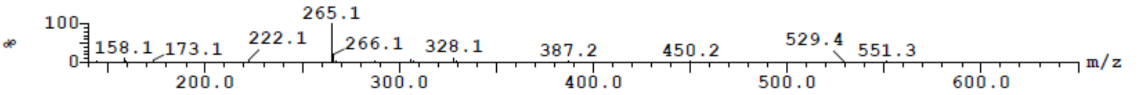

Sample: 65 Vial:1:G,6 File:I-0416627-001
Date:29-Aug-2018 Time:17:43:20

Printed: Thu Aug 30 10:30:57 2018

Sample Report (continued):

Peak ID	Time	Mass Found
4	1.92	

4:(Time: 1.89) 2:MS ES-
3.8e+005

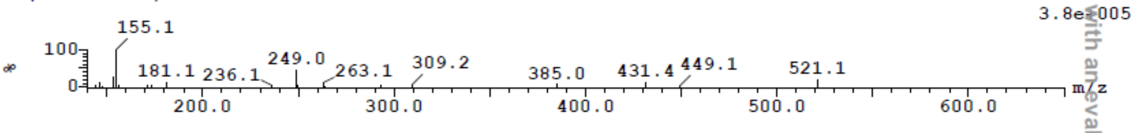

This file was generated with an evaluation version of the Amyuni(r) PDF Converter : http://www.amyuni.com

This file was generated with an eval...

N-(4-Hydroxybenzyl)-1-methyl-1H-indole-2-carboxamide (6d)

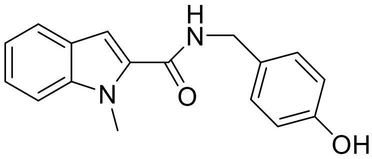

$C_{17}H_{16}N_2O_2$ **MW 280.33**

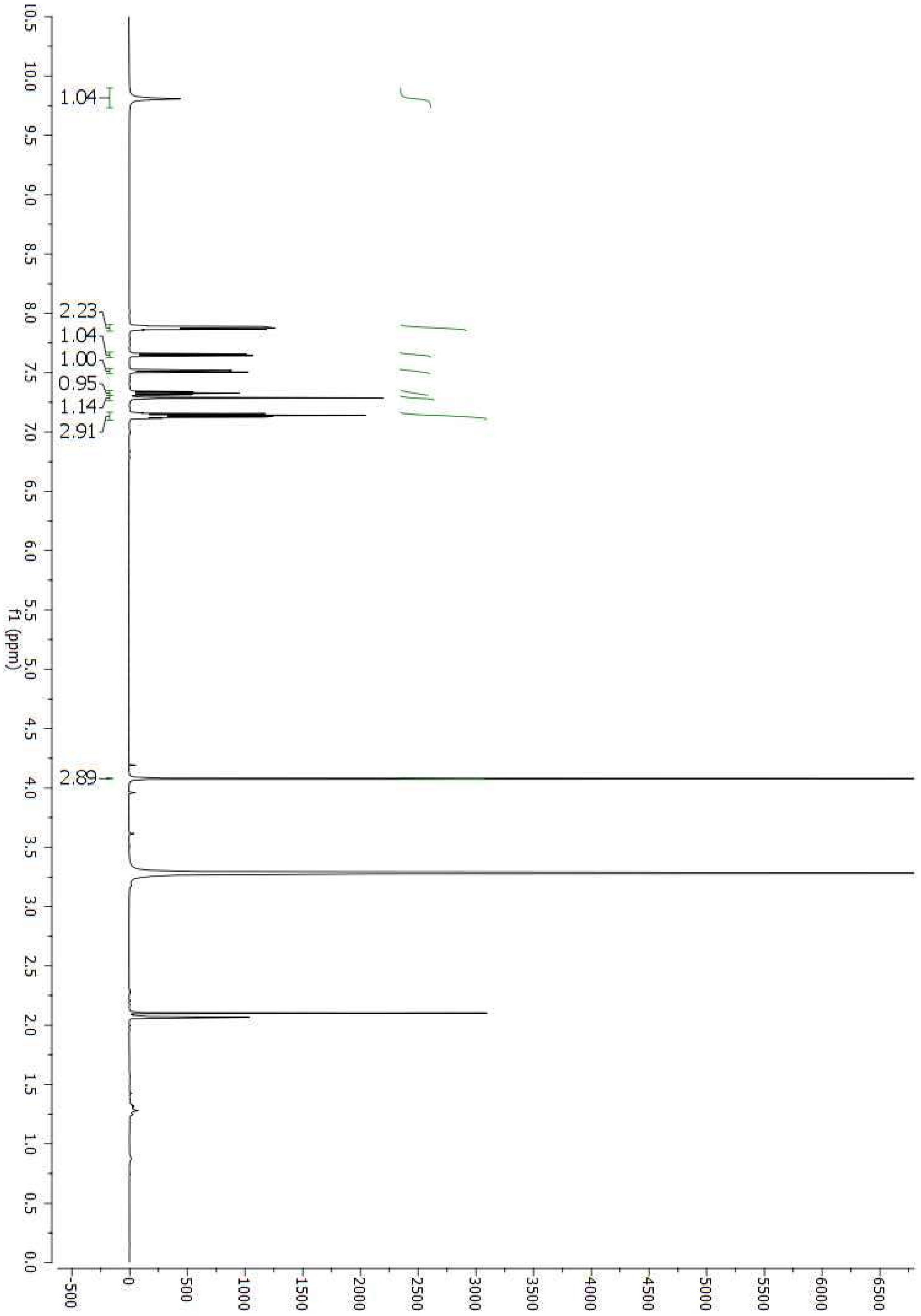

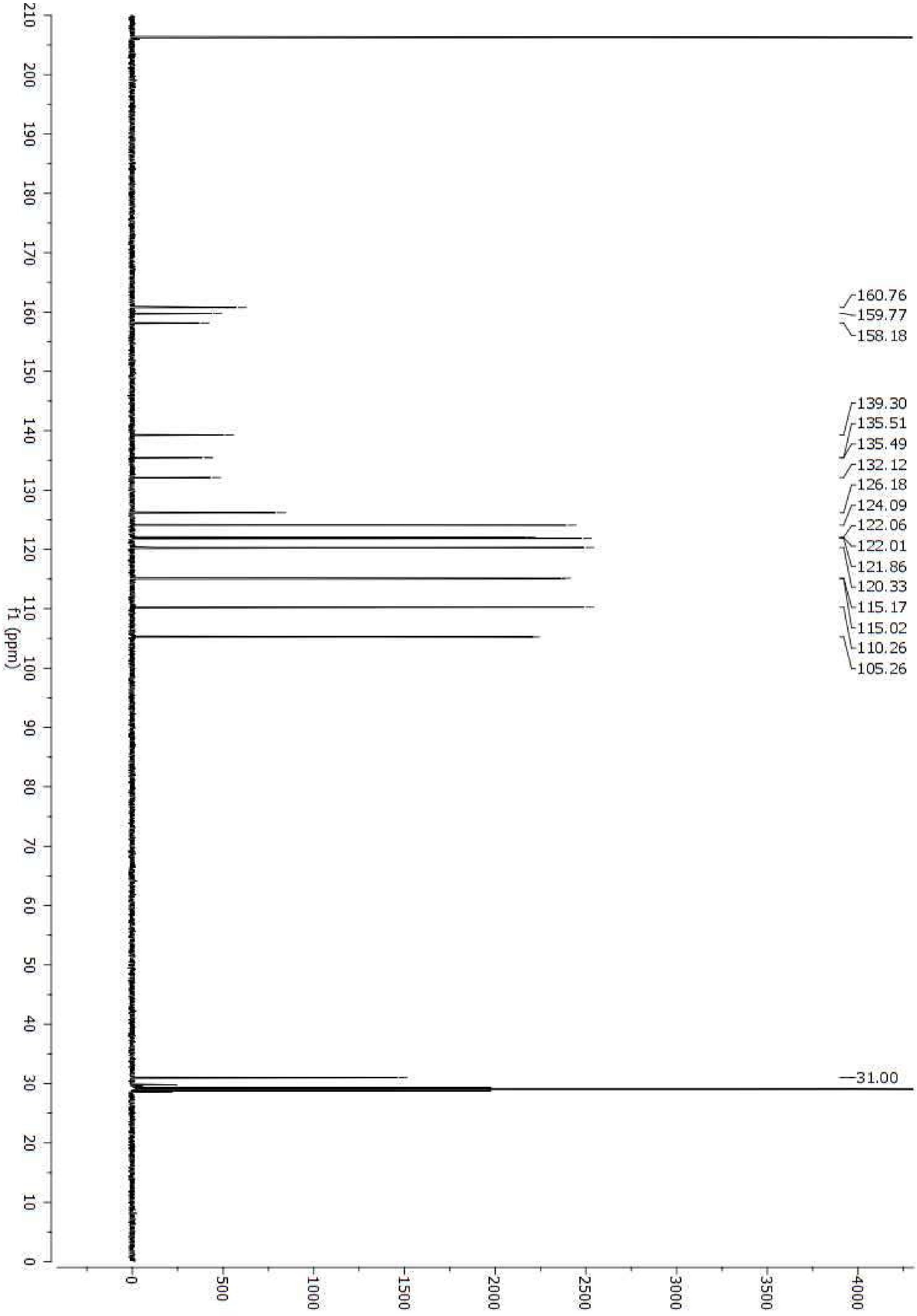
160.76
159.77
158.18
139.30
135.51
135.49
132.12
126.18
124.09
122.06
122.01
121.86
120.33
115.17
115.02
110.26
105.26
31.00
f1 (ppm)

Openlynx Report - Page 117
Sample: 58 Vial:1:F,9 File:I-0416621-001
Date:29-Aug-2018 Time:17:12:14

Printed: Thu Aug 30 10:30:57 2018

Sample Report (continued):

Sample 58 Vial 1:F,9 ID File I-0416621-001 Date 29-Aug-2018 Time 17:12:14 Description Met Gen

3: UV Detector: TIC 2.341e+2
Range: 2.341e+2

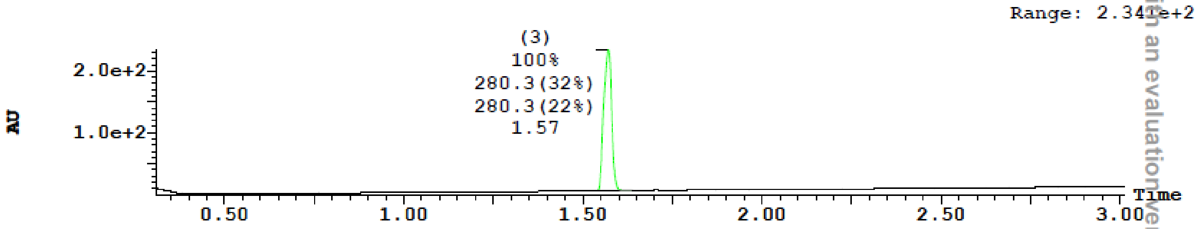

Peak Number	Time	Area %Total	Mass Found
3	1.57	100.00	280.32, 280.32

1: MS ES+ :TIC 1.2e+009

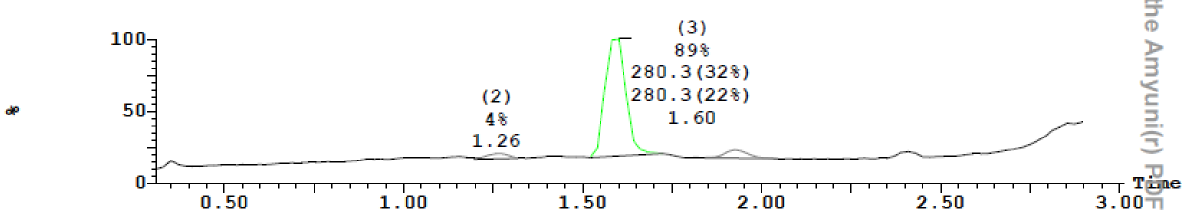

2: MS ES- :TIC 2.2e+008

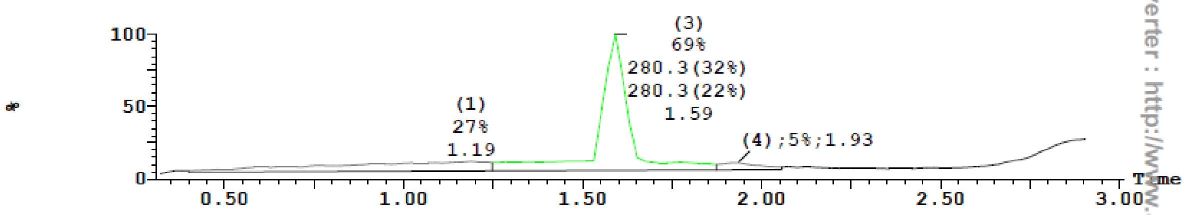

Peak ID	Time	Mass Found
3	1.60	281.32

3: (Time: 1.57) 1:MS ES+
1.1e+007

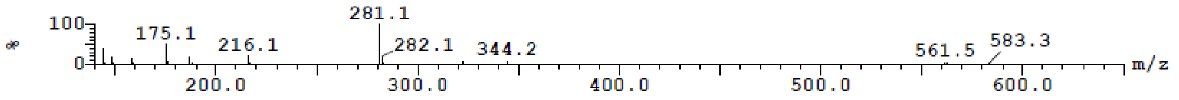

Openlynx Report - Page 118
Sample: 58 Vial:1:F,9 File:I-0416621-001
Date:29-Aug-2018 Time:17:12:14

Printed: Thu Aug 30 10:30:57 2018

Sample Report (continued):

Peak ID	Time	Mass Found
3	1.60	279.32

3: (Time: 1.57) 2:MS ES-
3.5e+006

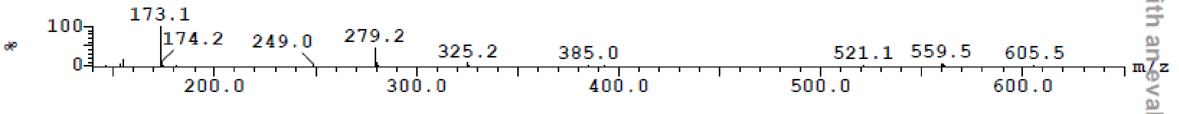

This file was generated with an evaluation version of the Amyuni(r) PDF Converter : http://www.amyuni.com
This file was generated with an eval

N-(4-Methoxybenzyl)-1-methyl-1*H*-indole-2-carboxamide (6e)

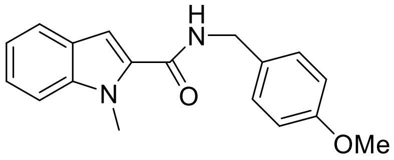

$C_{18}H_{18}N_2O_2$ **MW 294.35**

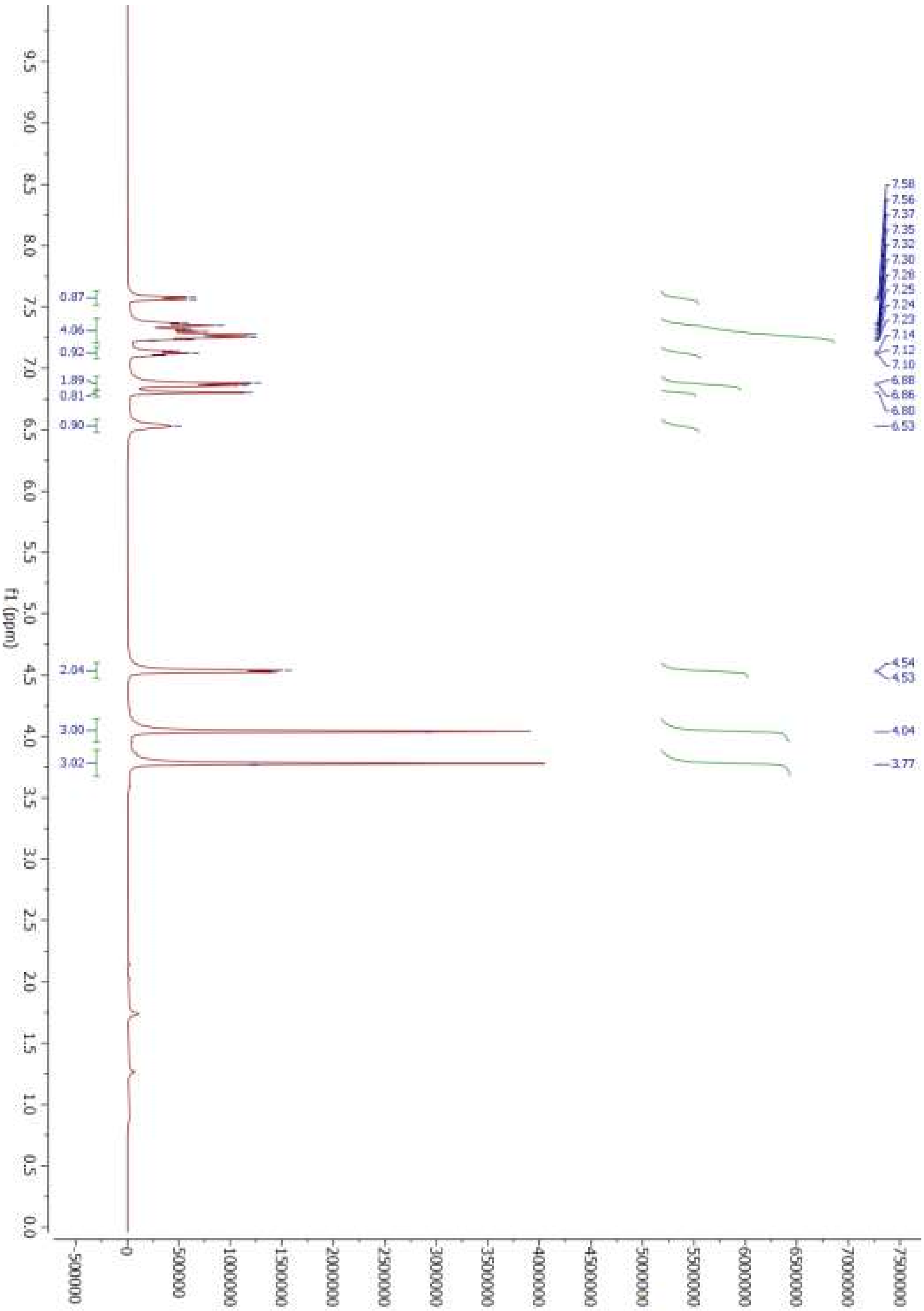

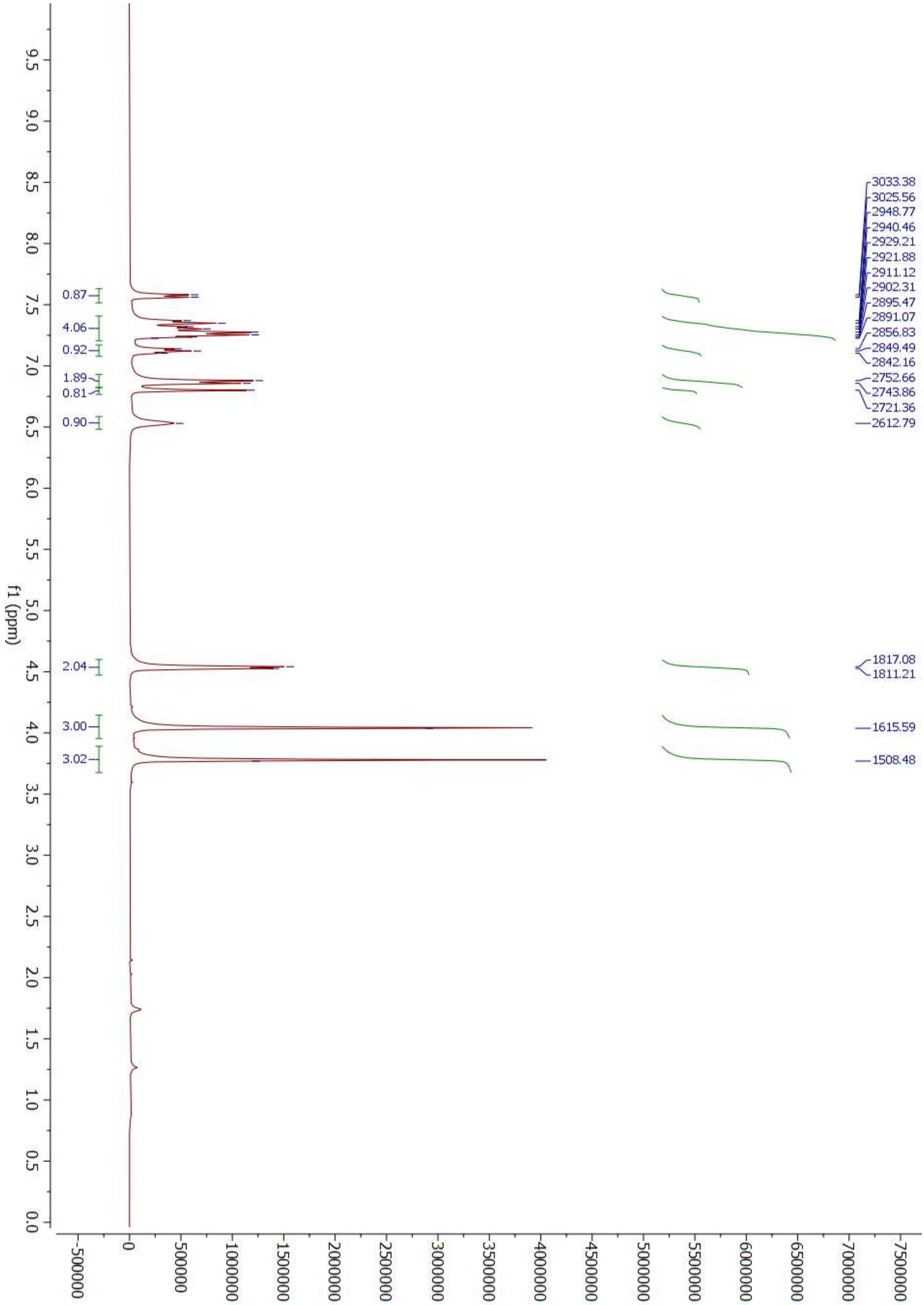
3033.38
3025.56
2948.77
2940.46
2929.21
2921.88
2911.12
2902.31
2895.47
2891.07
2856.83
2849.49
2842.16
2752.66
2743.86
2721.36
2612.79
1817.08
1811.21
1615.59
1508.48
0.87
4.06
0.92
1.89
0.81
0.90
2.04
3.00
3.02
f1 (ppm)

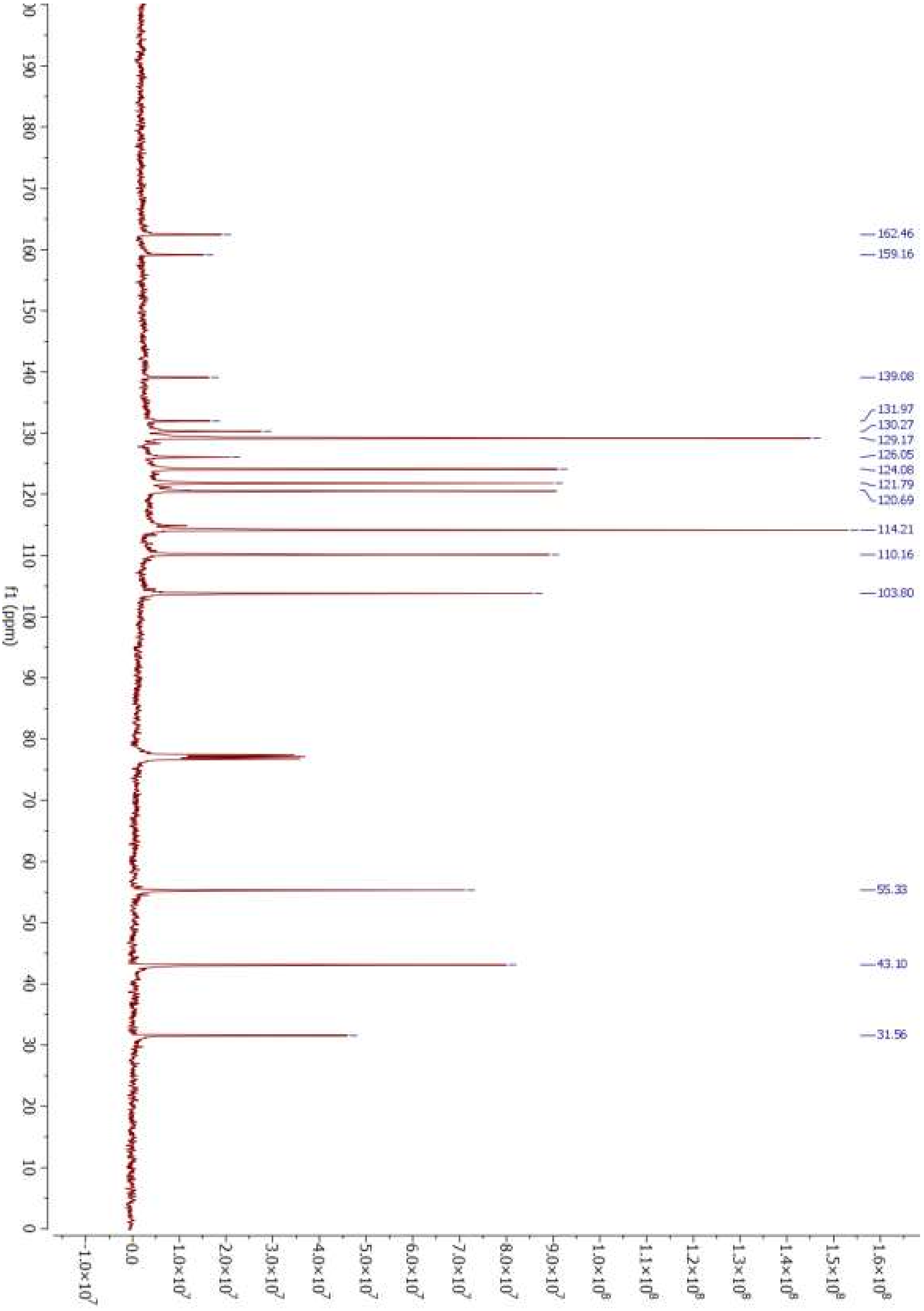
162.46
159.16
139.08
131.97
130.27
129.17
126.05
124.08
121.79
120.69
114.21
110.16
103.80
55.33
43.10
31.56
f1 (ppm)

Sample Report (continued):

Sample 29 Vial 1:C,10 ID File I-0416593-001 Date 29-Aug-2018 Time 15:03:44 Description Met Gen

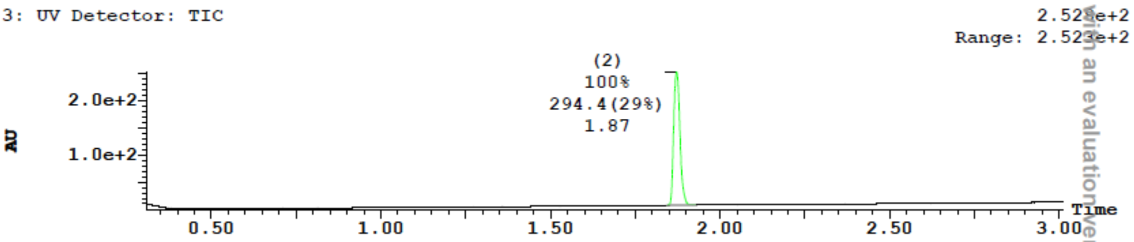

Peak Number	Time	Area %Total	Mass Found
2	1.87	100.00	294.35

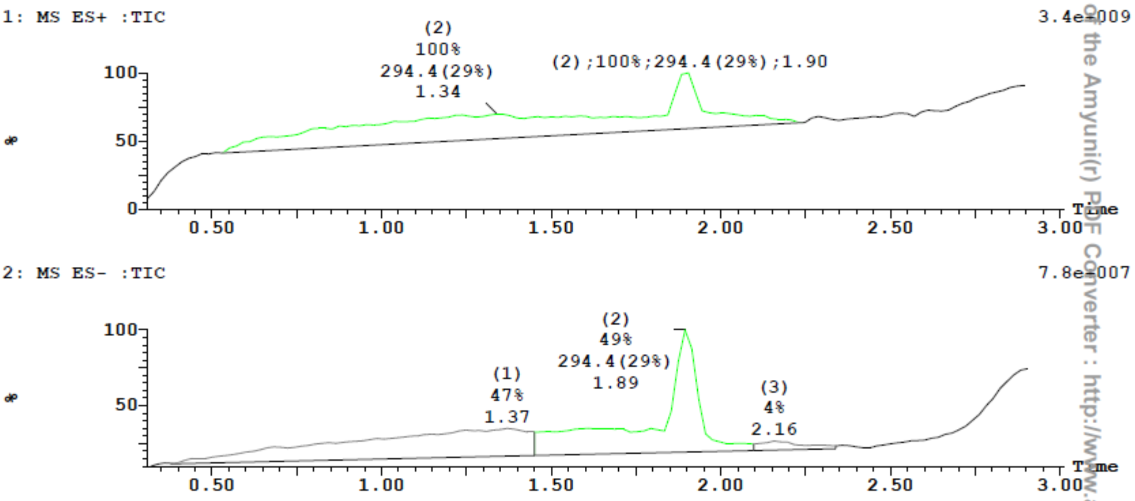

Peak ID	Time	Mass Found
2	1.90	295.35

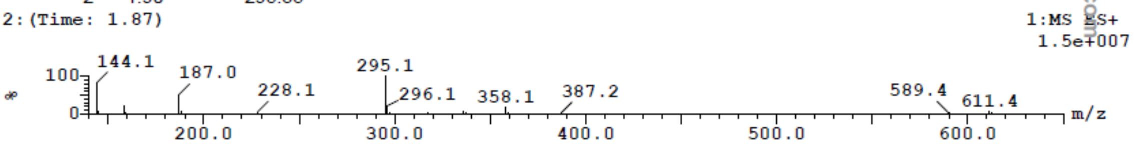

Sample Report (continued):

Peak ID	Time	Mass Found
2	1.90	

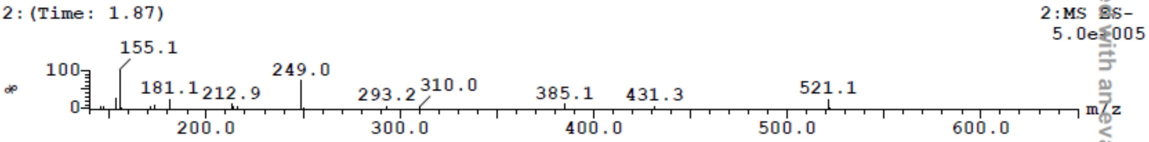

This file was generated with an evaluation version of the Amyuni(r) PDF Converter : http://www.amyuni.com

N-(4-Fluorobenzyl)-1-methyl-1H-indole-2-carboxamide (6f)

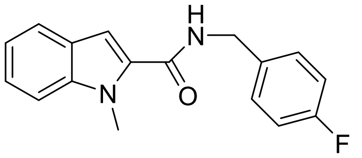

$C_{17}H_{15}FN_2O$ **MW 282.32**

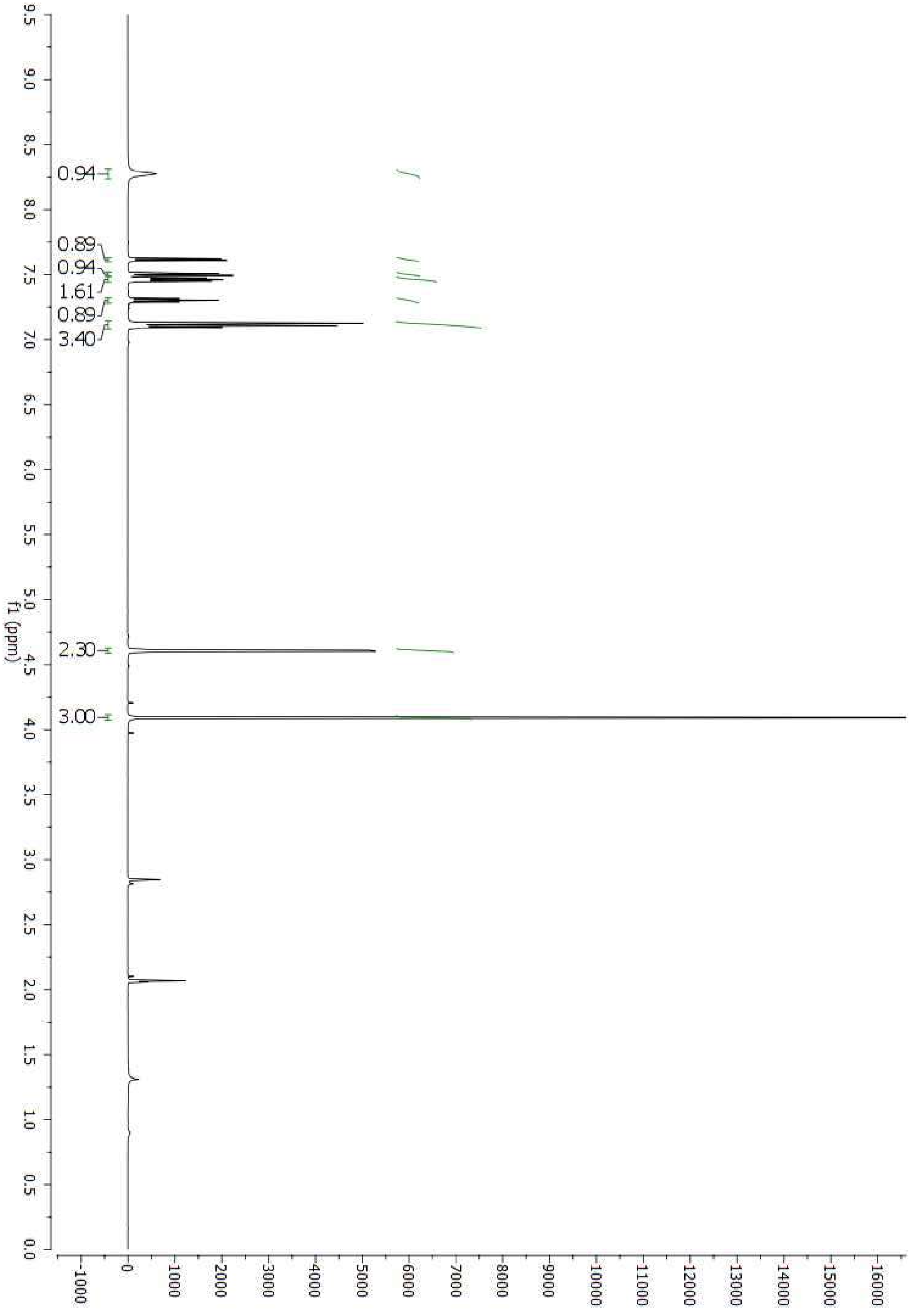

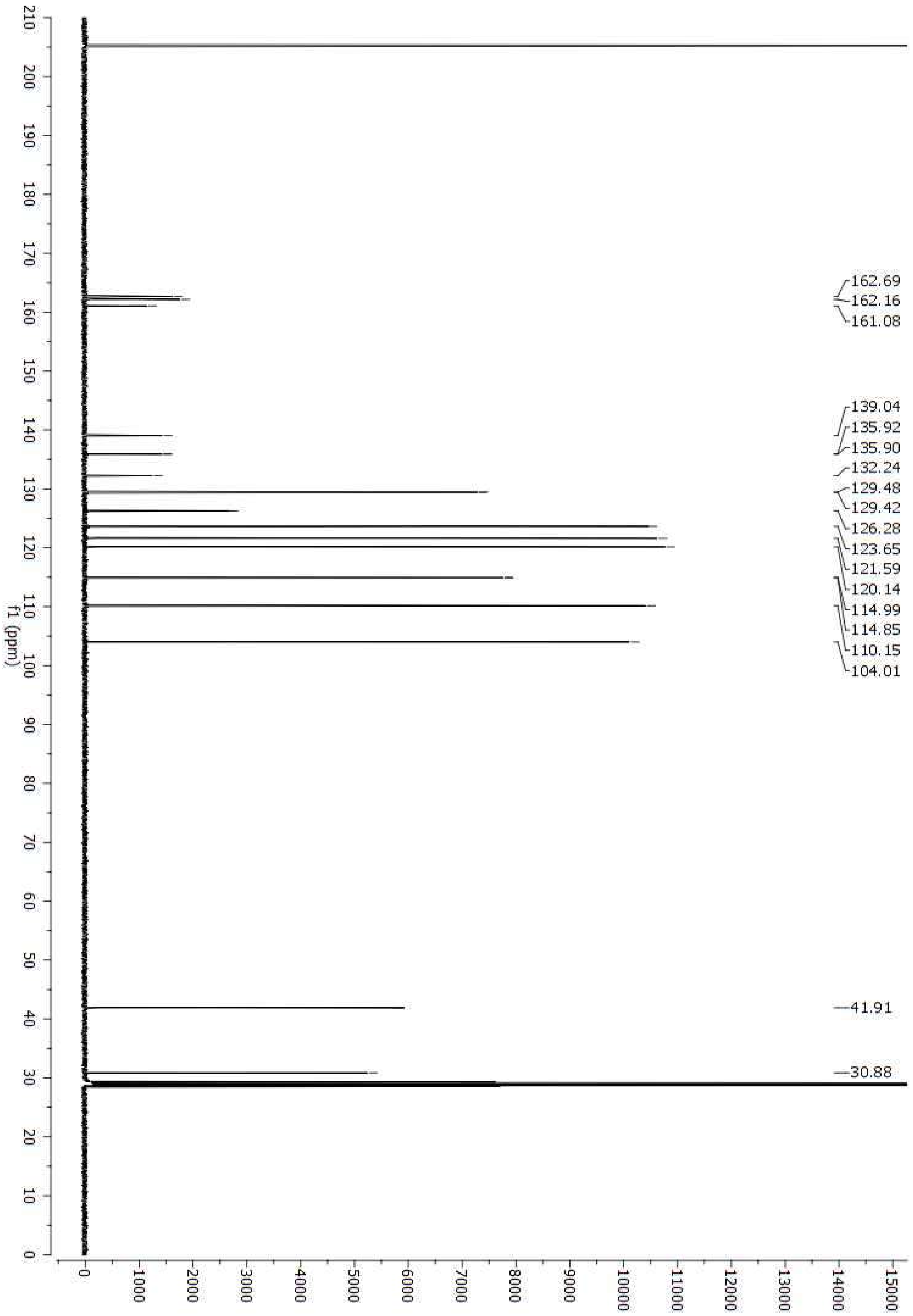
162.69
162.16
161.08
139.04
135.92
135.90
132.24
129.48
129.42
126.28
123.65
121.59
120.14
114.99
114.85
110.15
104.01
41.91
30.88
f1 (ppm)

Analysis Info

Method	MS_LC_POS_TimsOFF.m
Sample Name	MSP139

Acquisition Parameter

Source Type	ESI	Ion Polarity	Positive	Set Nebulizer	2.2 Bar
Focus	Active	Set Capillary	4000 V	Set Dry Heater	220 °C
Scan Begin	50 m/z	Set End Plate Offset	-500 V	Set Dry Gas	10.0 l/min
Scan End	2500 m/z	Set Collision Cell RF	1800.0 Vpp	Set Divert Valve	Waste

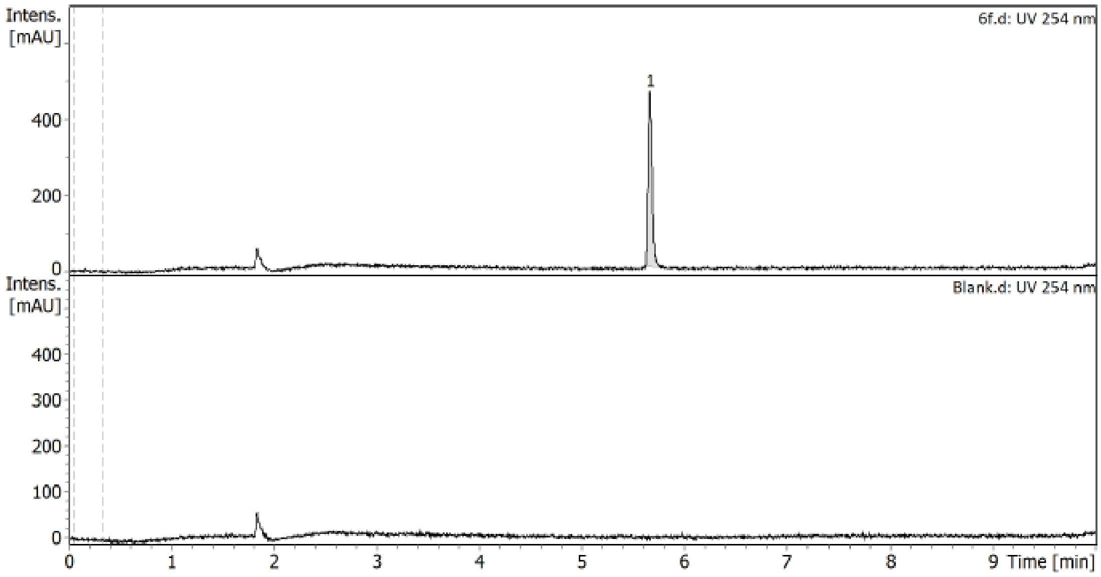

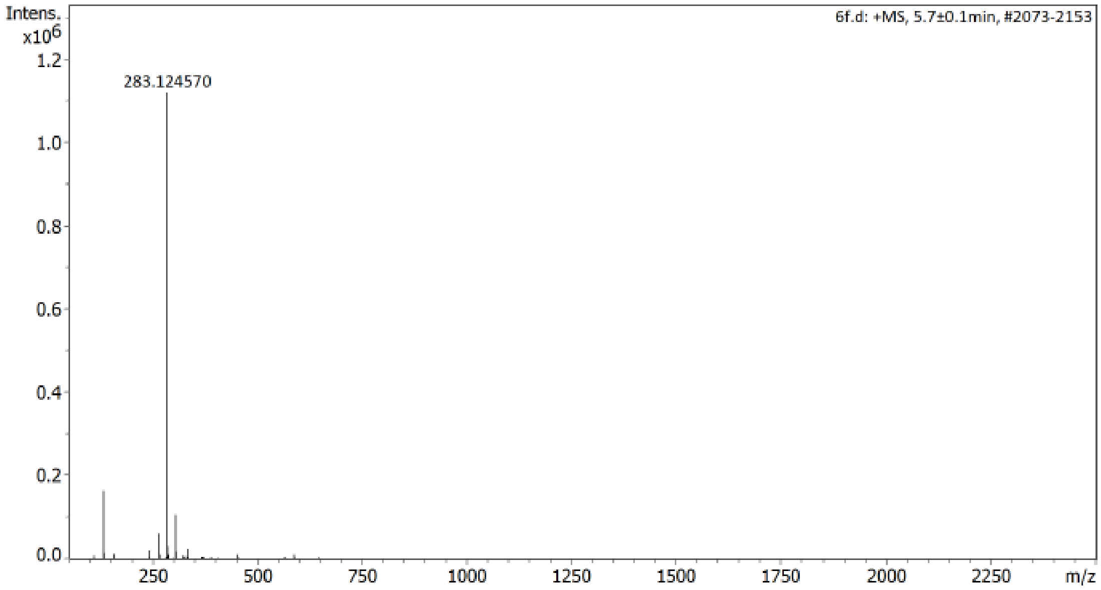

#	RT [min]	Area	Area Frac. %	I	S/N
1	5.7	1265.4	100.00	473	196.6

N-(4-Hydroxy-3-methoxybenzyl)-1-methyl-1H-indole-2-carboxamide (6g)

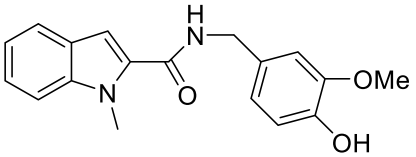

$C_{18}H_{18}N_2O_3$ **MW 310.35**

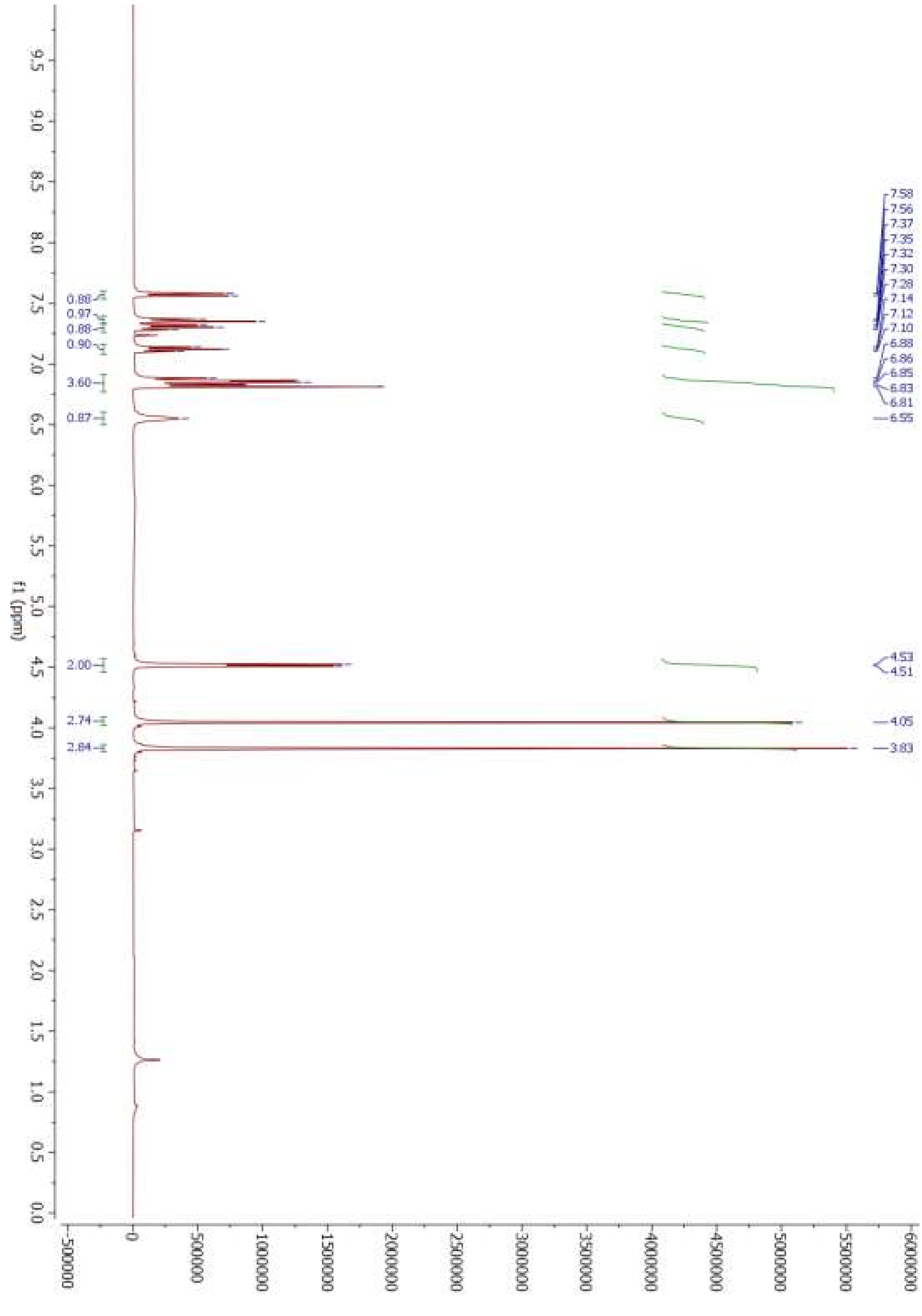

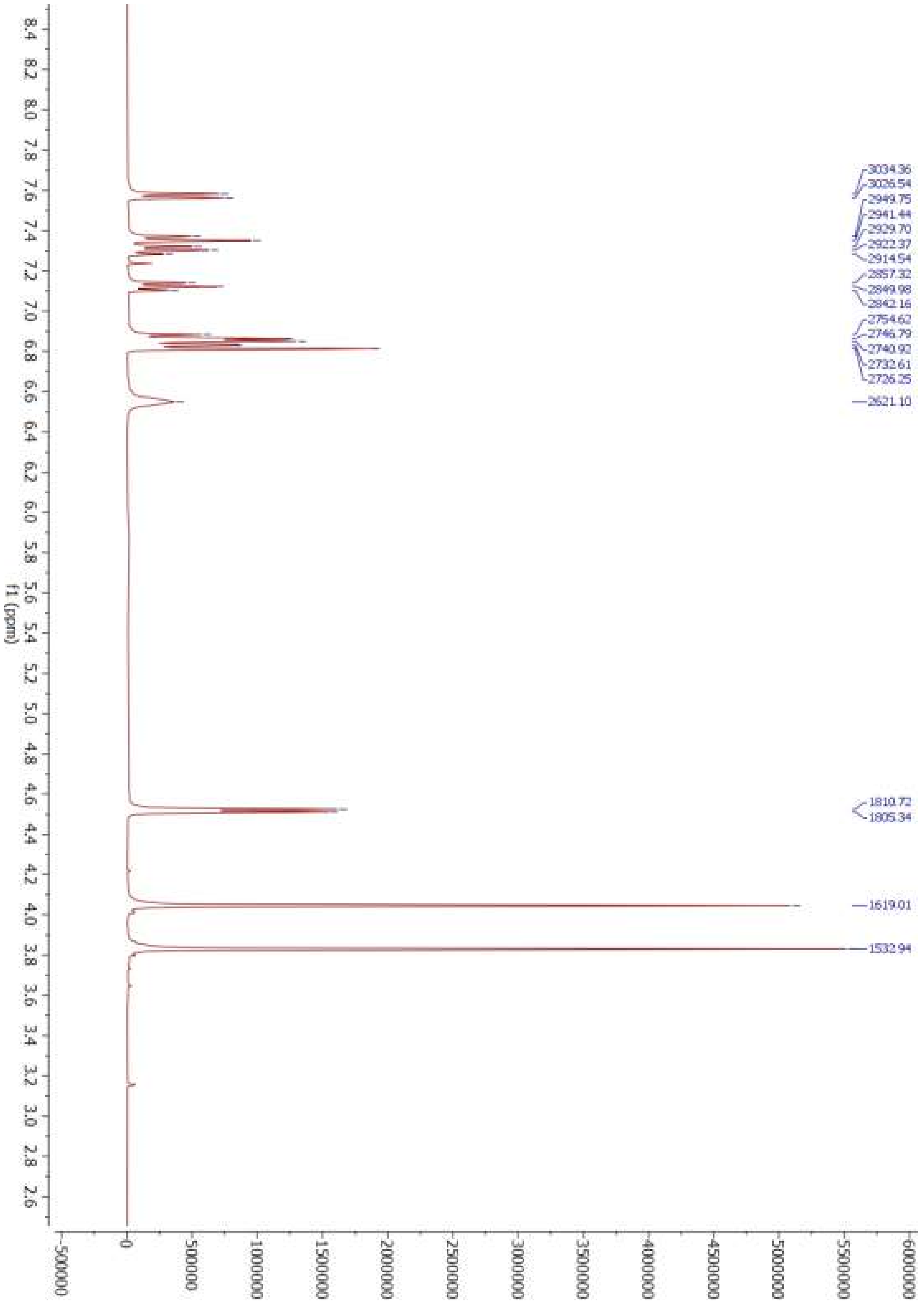
3034.36
3026.54
2949.75
2941.44
2929.70
2922.37
2914.54
2857.32
2849.98
2842.16
2754.62
2746.79
2740.92
2732.61
2726.25
2621.10
1810.72
1805.34
1619.01
1532.94
f1 (ppm)

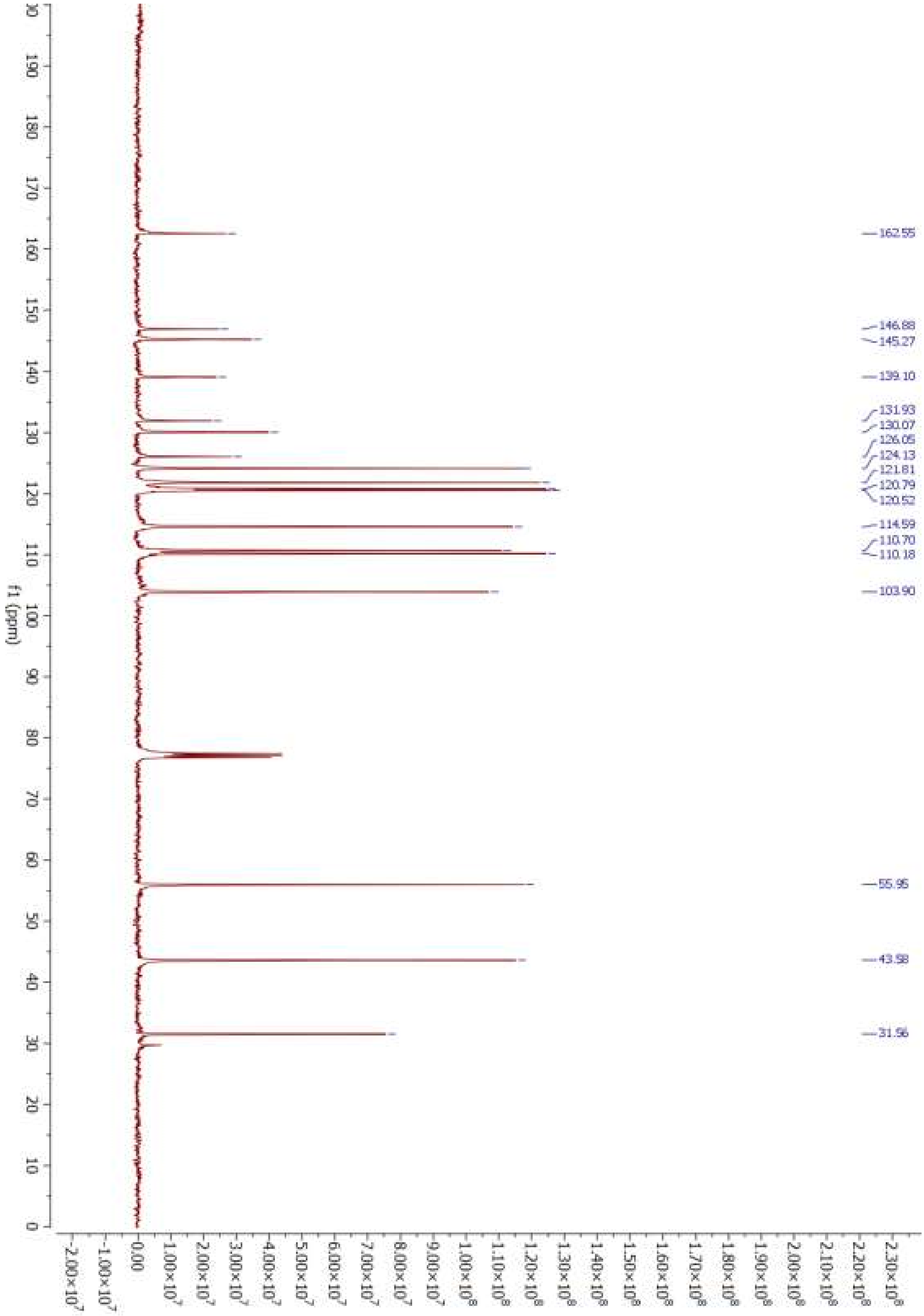
162.55
146.88
145.27
139.10
131.93
130.07
126.05
124.13
121.81
120.79
120.52
114.59
110.70
110.18
103.90
55.95
43.58
31.56
f1 (ppm)

Sample Report (continued):

Sample 27 Vial 1:C,8 ID File I-0416591-001 Date 29-Aug-2018 Time 14:54:50 Description Met Gen

3: UV Detector: TIC 2.41e+2
Range: 2.40e+2

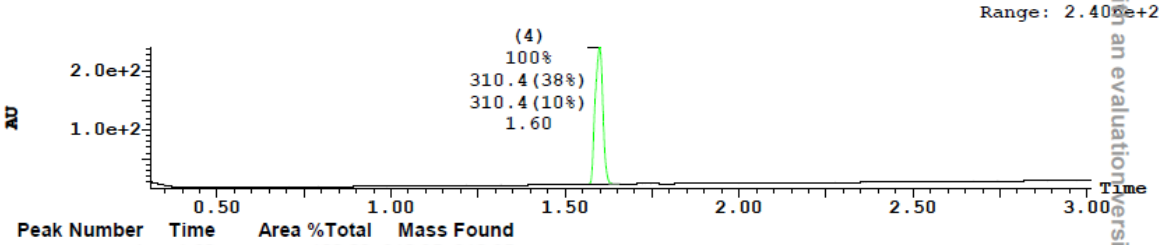

Peak Number	Time	Area %Total	Mass Found
4	1.60	100.00	310.35, 310.35

1: MS ES+ :TIC 3.6e009

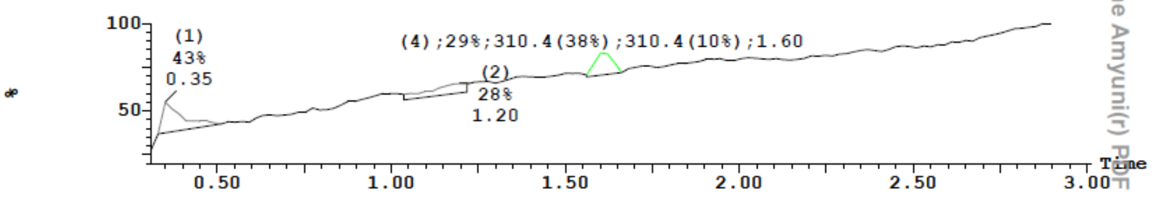

2: MS ES- :TIC 1.1e008

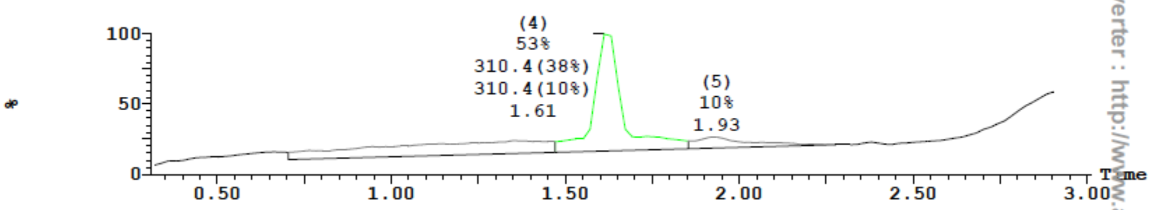

Peak ID	Time	Mass Found
4	1.60	311.35

4:(Time: 1.60) 1:MS ES+
1.9e+007

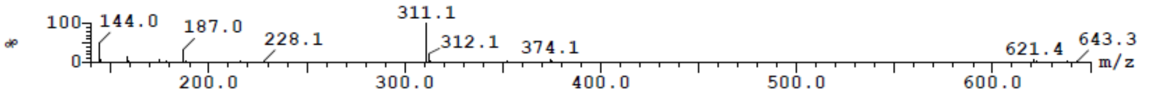

Openlynx Report -
Sample: 27 Vial:1:C,8 File:I-0416591-001 Page 56
Date:29-Aug-2018 Time:14:54:50

Printed: Thu Aug 30 10:30:57 2018

Sample Report (continued):

Peak ID	Time	Mass Found
4	1.60	309.35

4:(Time: 1.60) 2:MS ES-
1.1e006

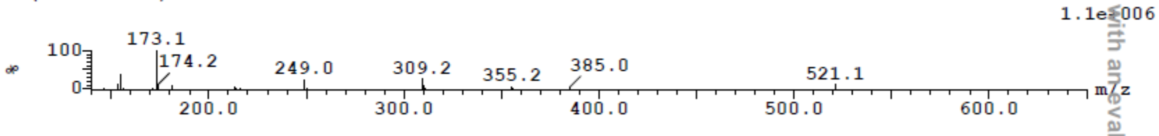

This file was generated with an evaluation version of the Amyuni(r) PDF Converter : http://www.amyuni.com
This file was generated with an evalu

N-(3,4-Dichlorobenzyl)-1-methyl-1H-indole-2-carboxamide (6h)

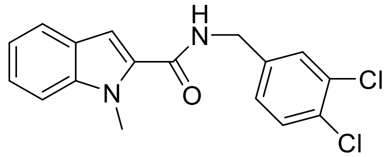

$C_{17}H_{14}Cl_2N_2O$ **MW 333.21**

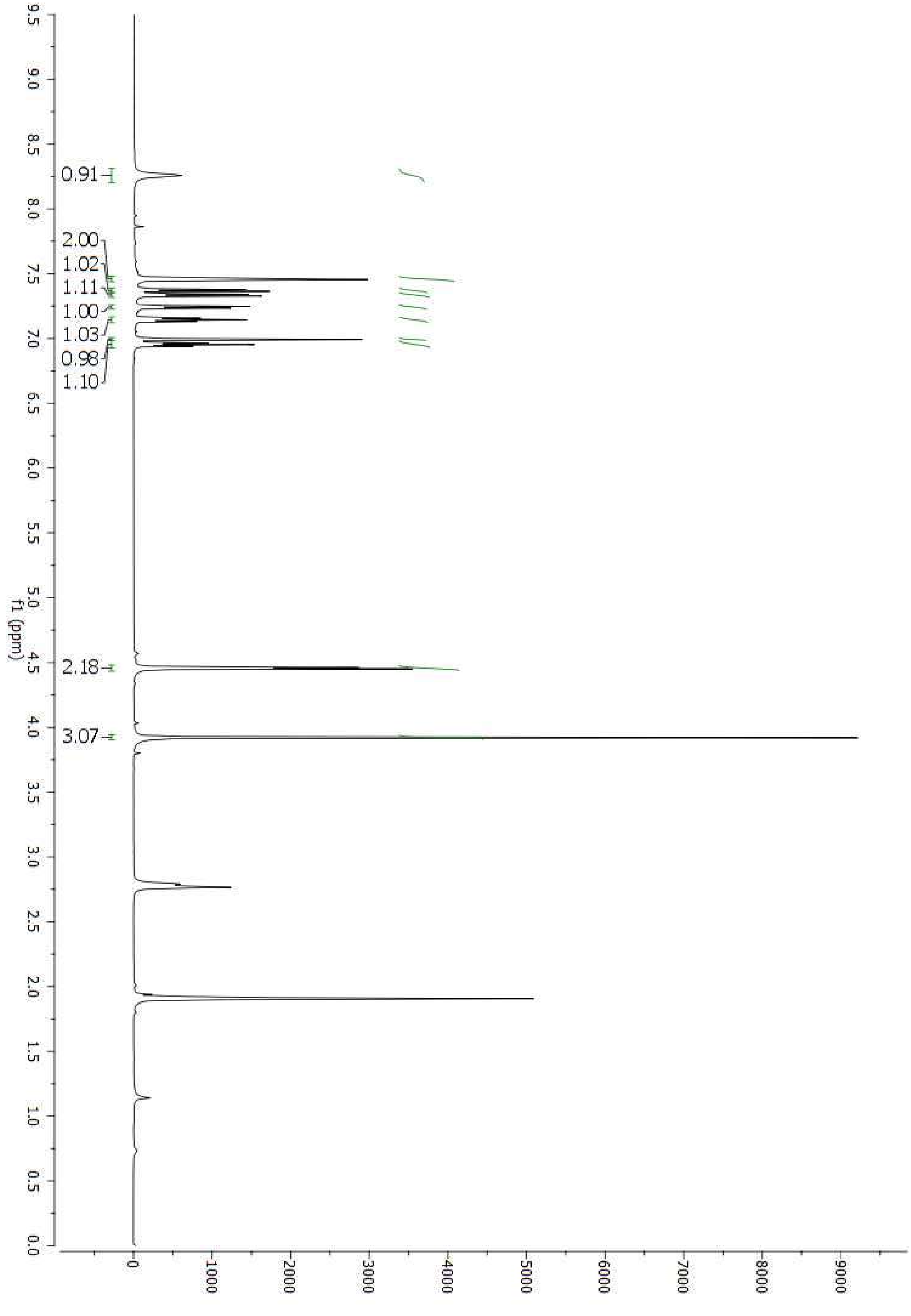

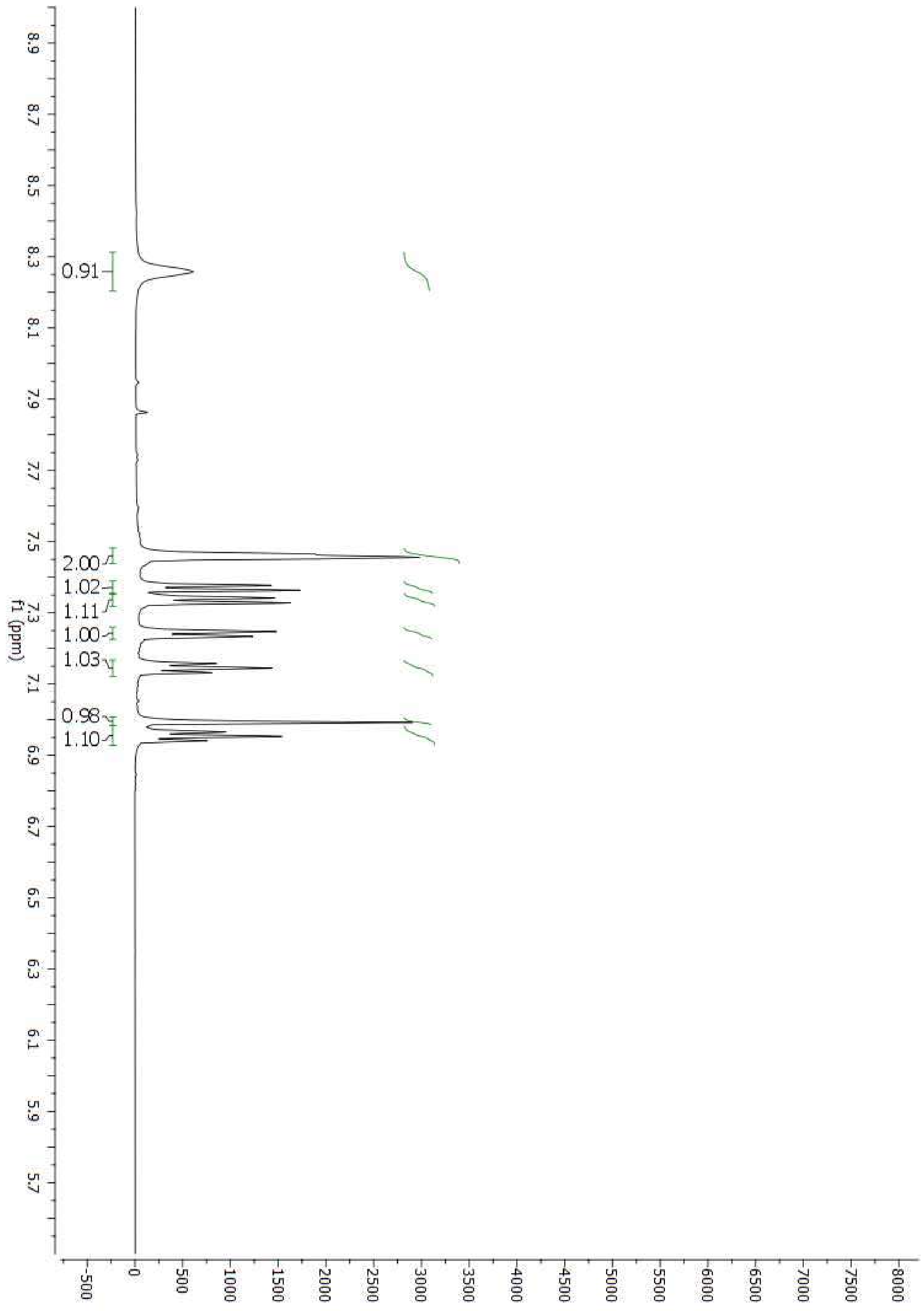
f1 (ppm)
0.91
2.00
1.02
1.11
1.00
1.03
0.98
1.10

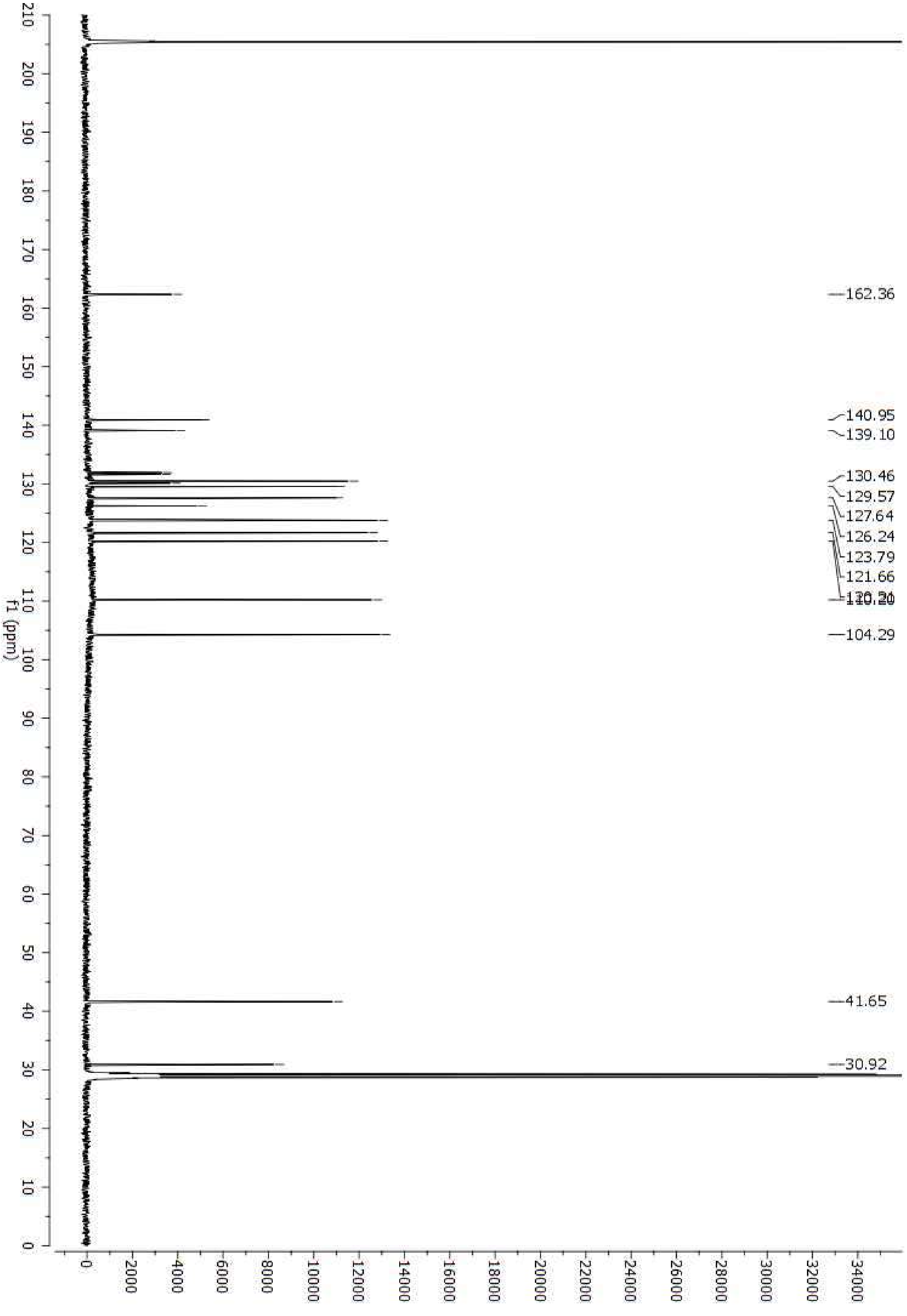
162.36
140.95
139.10
130.46
129.57
127.64
126.24
123.79
121.66
110.21
110.20
104.29
41.65
30.92
f1 (ppm)

Sample Report (continued):

Sample 26 Vial 1:C,7 ID File I-0416590-001 Date 29-Aug-2018 Time 14:50:24 Description Met Gen

3: UV Detector: TIC 2.48e+2
Range: 2.476e+2

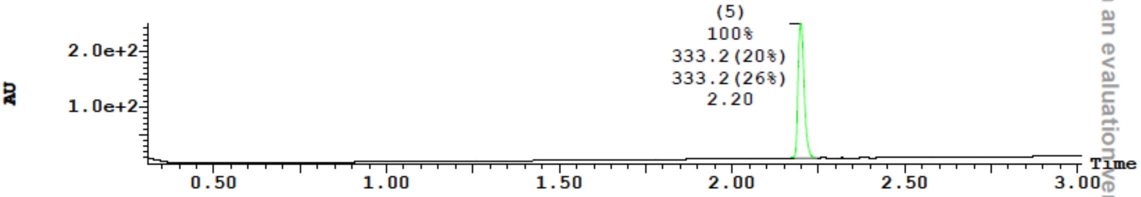

Peak Number	Time	Area %Total	Mass Found
5	2.20	100.00	333.21, 333.21

1: MS ES+ :TIC 3.0e+009

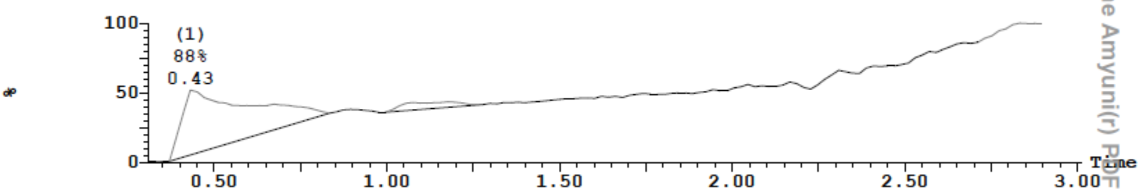

2: MS ES- :TIC 2.6e+008

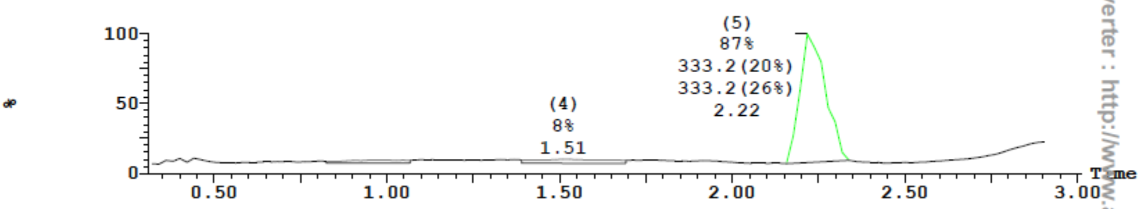

Peak ID	Time	Mass Found
5	2.22	334.21

5:(Time: 2.20) 1:MS ES+
1.0e+006

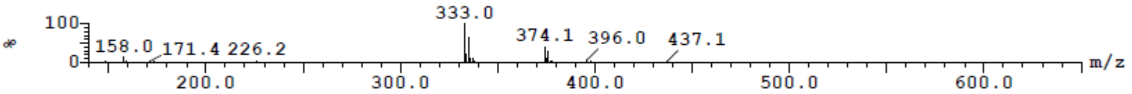

Sample Report (continued):

Peak ID	Time	Mass Found
5	2.22	332.21

5:(Time: 2.20) 2:MS ES-
3.1e+006

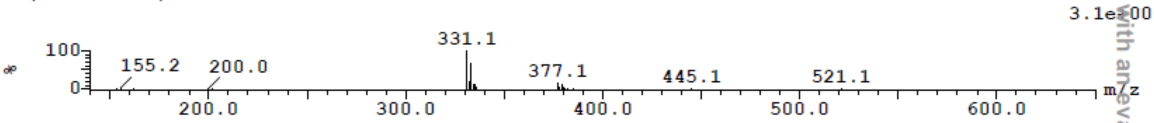

This file was generated with an evaluation version of the Amyuni(r) PDF Converter : http://www.amyuni.com
This file was generated with an evalu

N-(3,4-Difluorobenzyl)-1-methyl-1H-indole-2-carboxamide (6i)

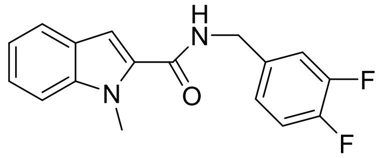

$C_{17}H_{14}F_2N_2O$ **MW 300.31**

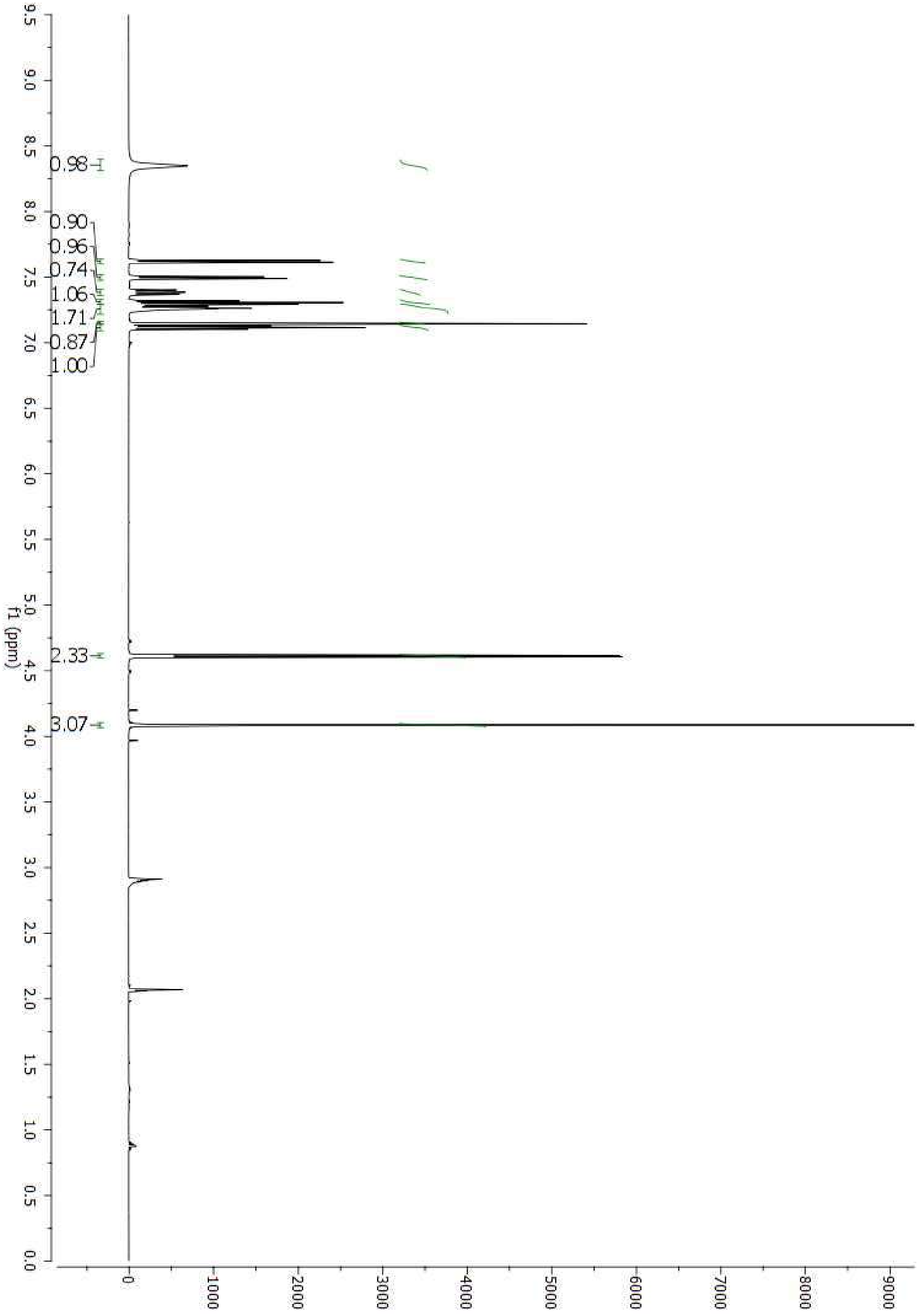

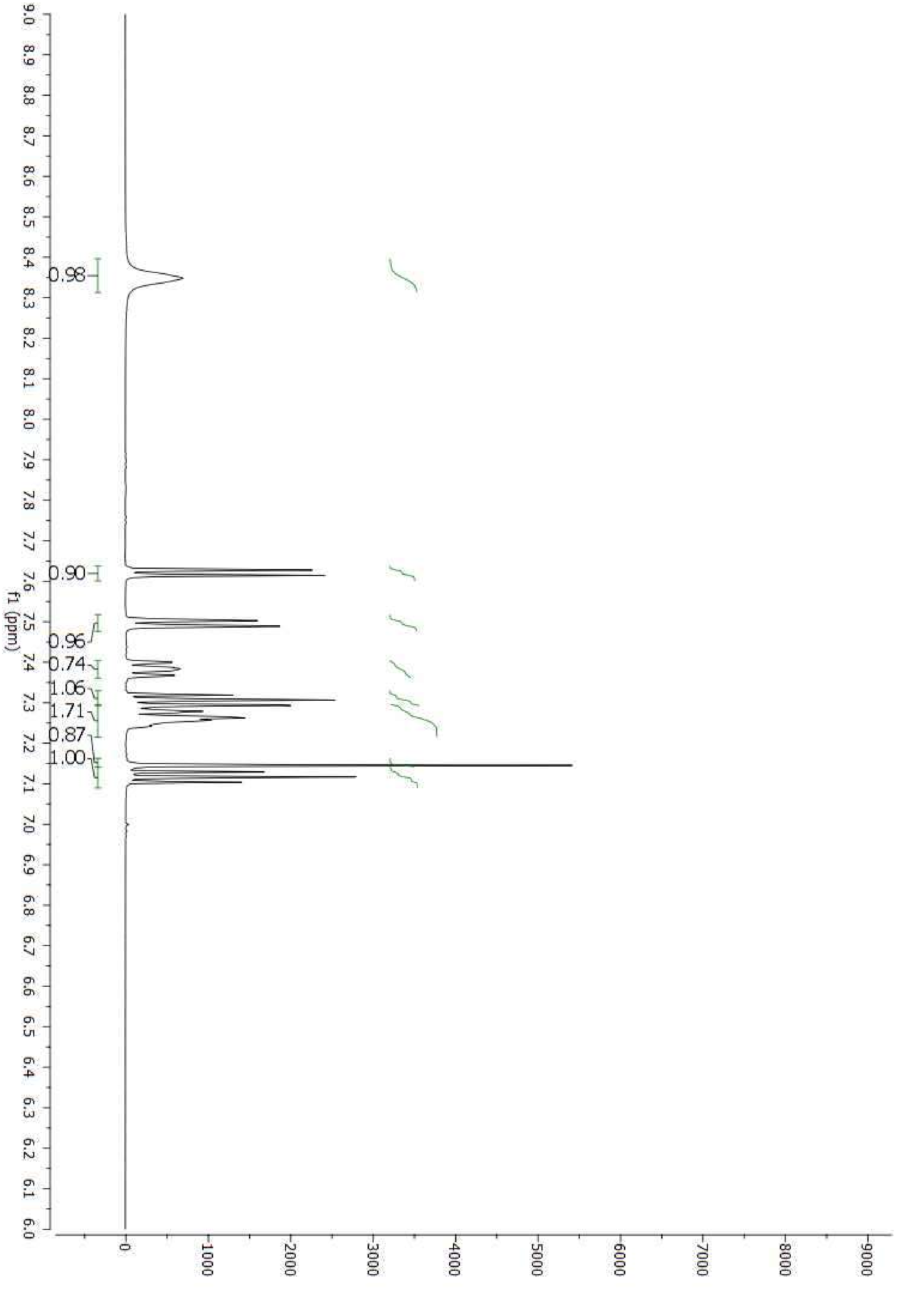
0.98
0.90
0.96
0.74
1.06
1.71
0.87
1.00
f1 (ppm)

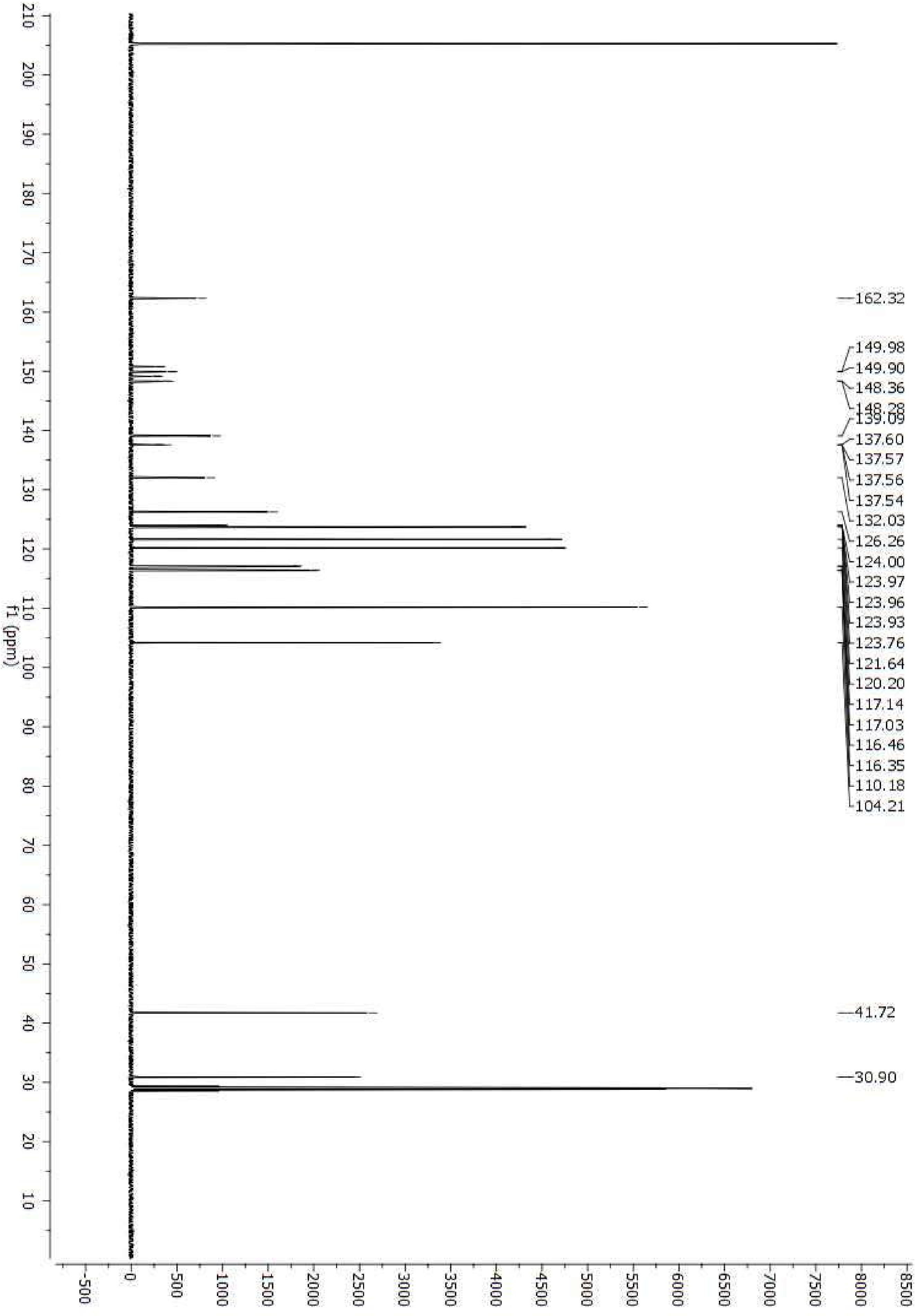

162.32
149.98
149.90
148.36
148.28
139.09
137.60
137.57
137.56
137.54
132.03
126.26
124.00
123.97
123.96
123.93
123.76
121.64
120.20
117.14
117.03
116.46
116.35
110.18
104.21
41.72
30.90
f1 (ppm)

Analysis Info

Method	MS_LC_POS_TimsOFF.m
Sample Name	MSP140

Acquisition Parameter

Source Type	ESI	Ion Polarity	Positive	Set Nebulizer	2.2 Bar
Focus	Active	Set Capillary	4000 V	Set Dry Heater	220 °C
Scan Begin	50 m/z	Set End Plate Offset	-500 V	Set Dry Gas	10.0 l/min
Scan End	2500 m/z	Set Collision Cell RF	1800.0 Vpp	Set Divert Valve	Waste

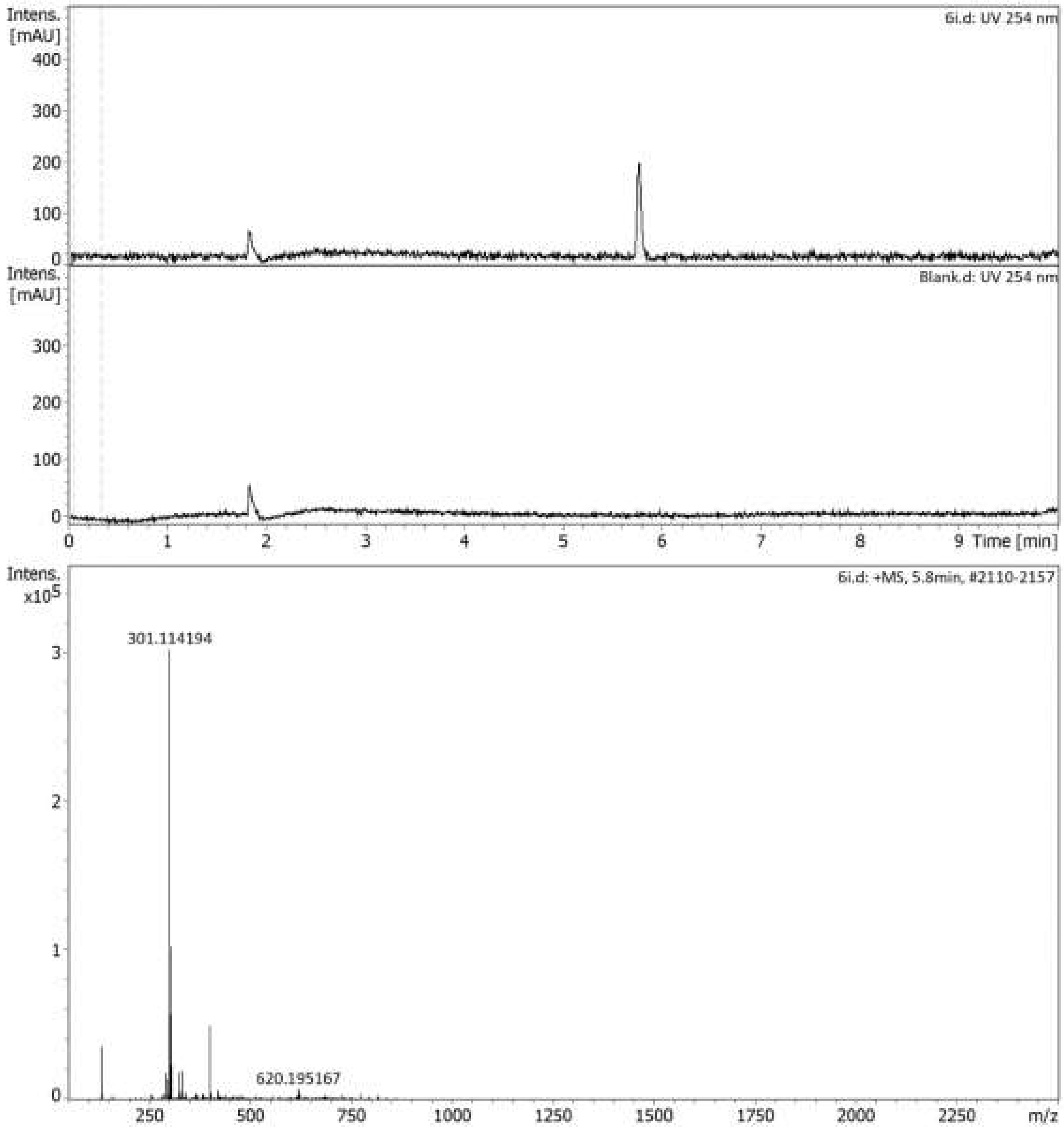

#	RT [min]	Area	Area Frac. %	I	S/N
2	5.8	508.34	100.00	199	24.7

N-(3,4-Dihydroxyphenethyl)-1-methyl-1H-indole-2-carboxamide (6j)

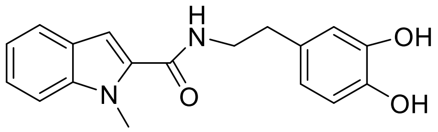

$C_{18}H_{18}N_2O_3$ **MW 310.35**

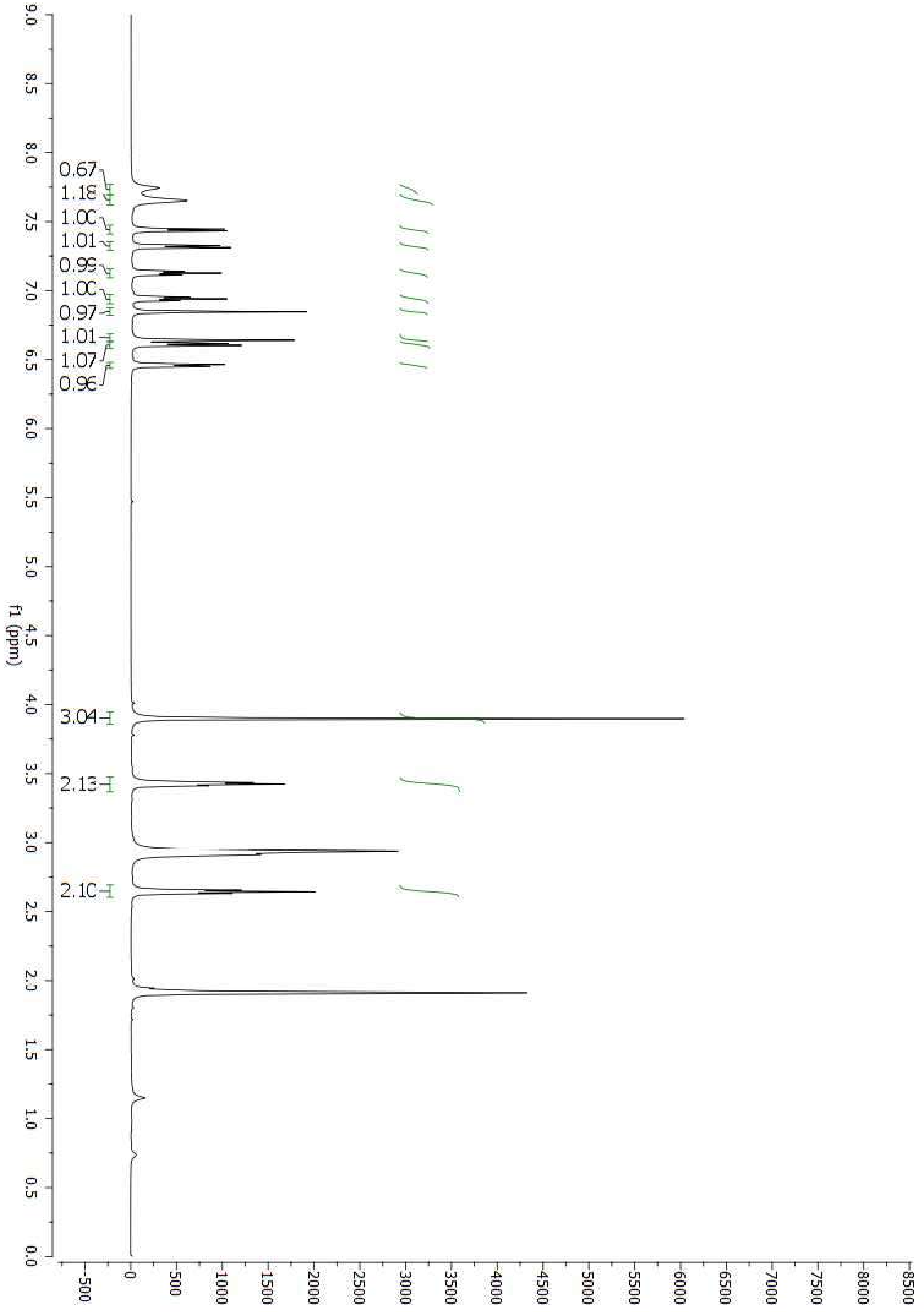

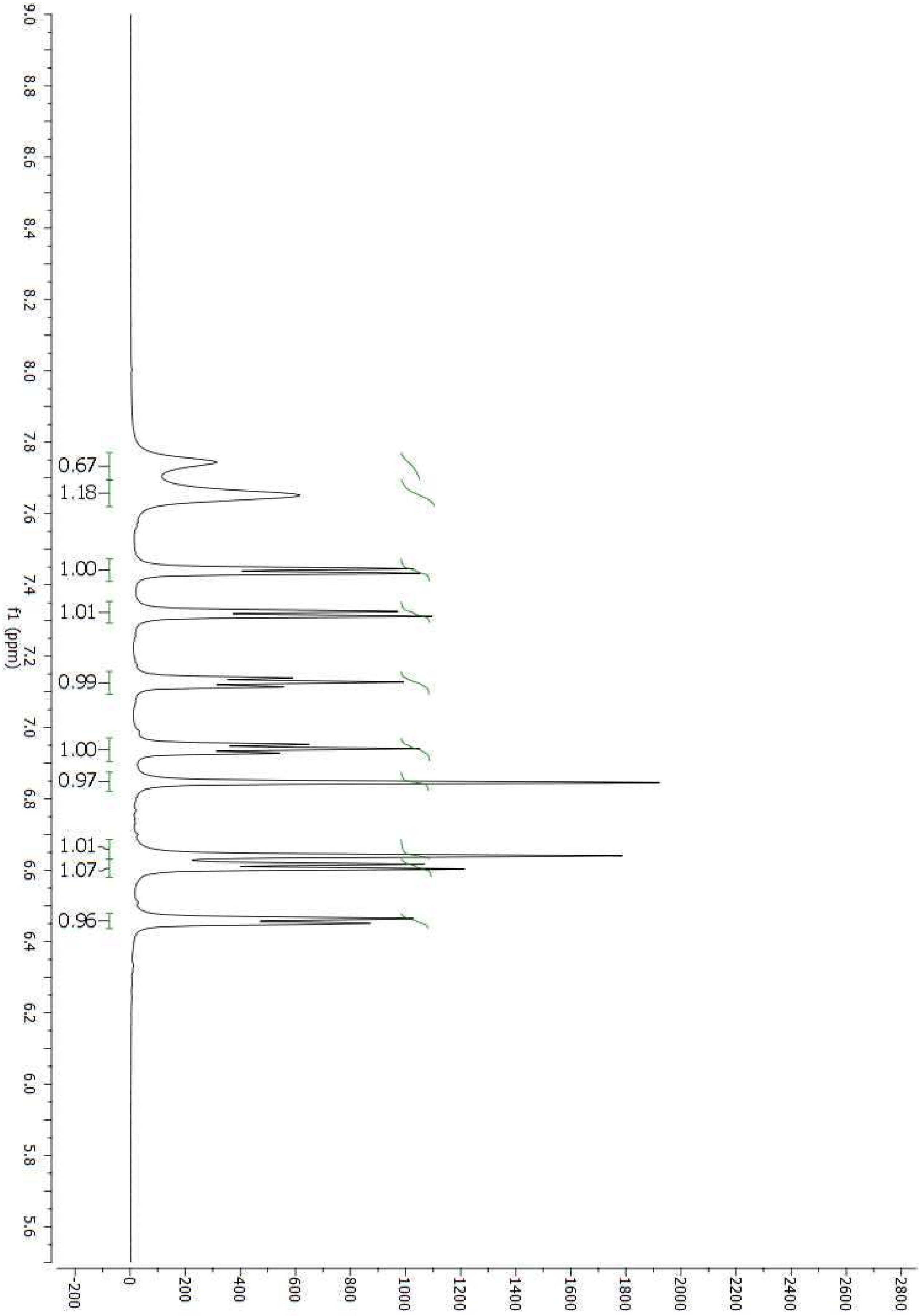
f1 (ppm)
0.67
1.18
1.00
1.01
0.99
1.00
0.97
1.01
1.07
0.96

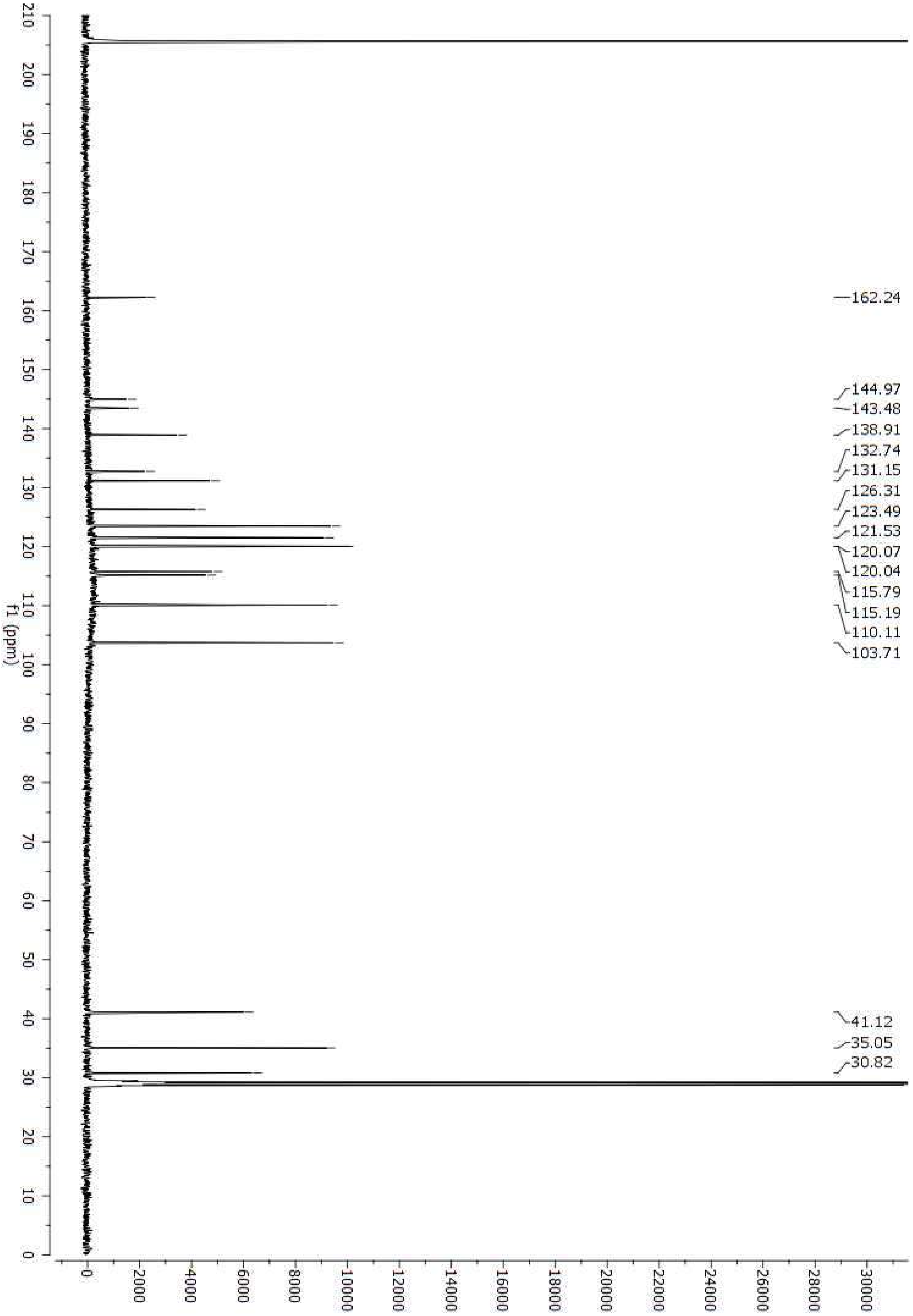
162.24
144.97
143.48
138.91
132.74
131.15
126.31
123.49
121.53
120.07
120.04
115.79
115.19
110.11
103.71
41.12
35.05
30.82
f1 (ppm)

Analysis Info

Method	MS_LC_POS_TimsOFF.m
Sample Name	MSP68 sol.madre

Acquisition Parameter

Source Type	ESI	Ion Polarity	Positive	Set Nebulizer	2.2 Bar
Focus	Active	Set Capillary	4000 V	Set Dry Heater	220 °C
Scan Begin	50 m/z	Set End Plate Offset	-500 V	Set Dry Gas	10.0 l/min
Scan End	2500 m/z	Set Collision Cell RF	1800.0 Vpp	Set Divert Valve	Waste

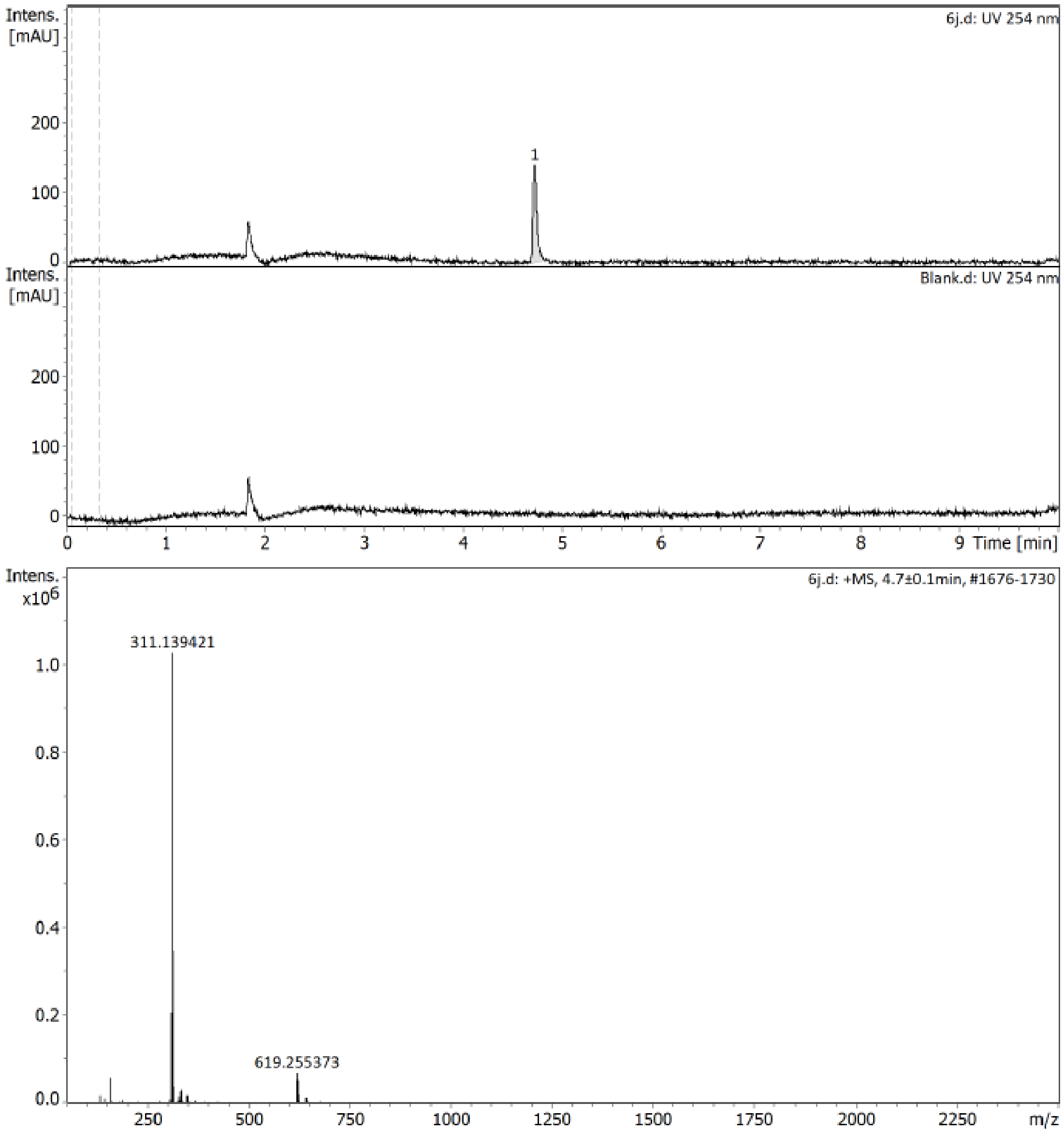

#	RT [min]	Area	Area Frac. %	I	S/N
1	4.7	394.59	100.00	139	57.2
